# Supplementary material for: International patient preferences for physician attire: results from cross-sectional studies in four countries across three continents
Source: BMJ Open. 2022 Oct 3;12(10):e061092. doi: 10.1136/bmjopen-2022-061092 (PMC9535197; doi:10.1136/bmjopen-2022-061092)
Supplement: Supplementary data [file bmjopen-2022-061092supp001.pdf]

Appendix A: Survey Photographs by Country

	Casual	Casual with white coat	Scrubs	Scrubs with white coat	Formal	Formal with white coat	Business suit
**Italy and the United States**	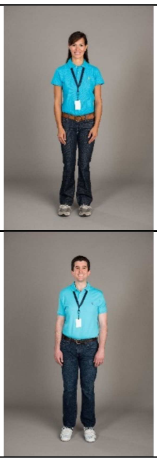	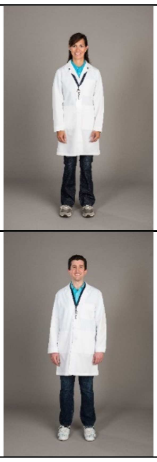	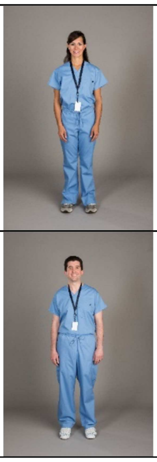	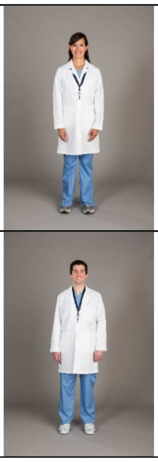	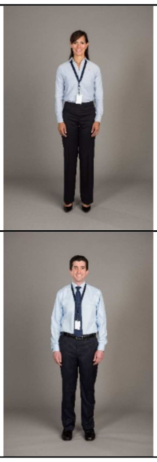	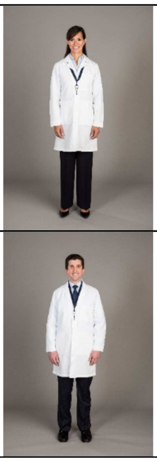	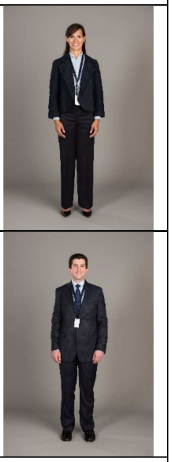
**Switzerland**	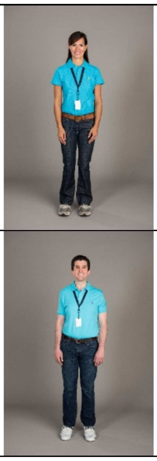	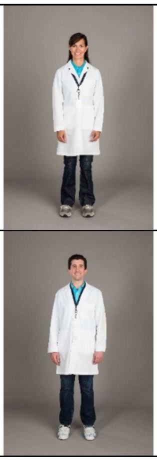	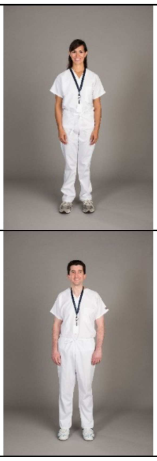	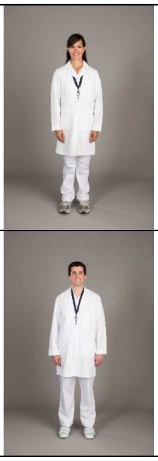	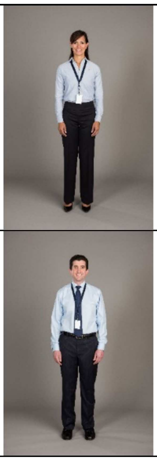	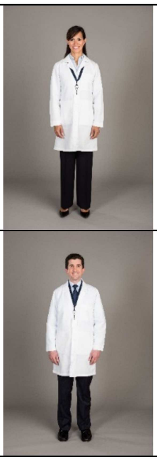	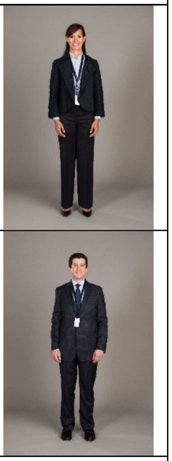
**Japan**	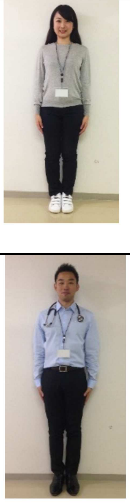	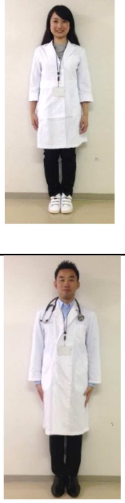	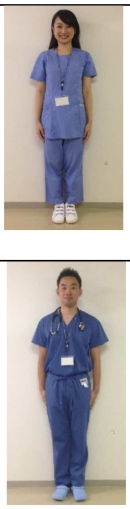	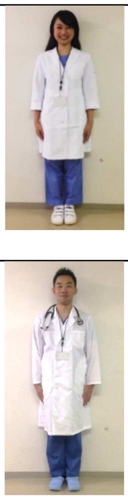	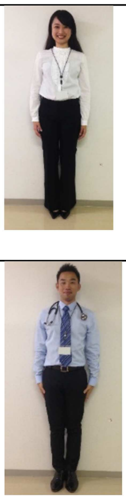	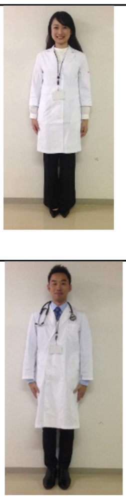	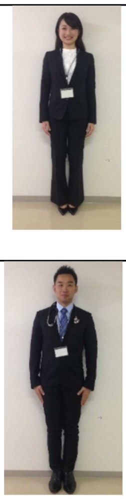
