# Supplementary material for: International patient preferences for physician attire: results from cross-sectional studies in four countries across three continents
Source: BMJ Open. 2022 Oct 3;12(10):e061092. doi: 10.1136/bmjopen-2022-061092 (PMC9535197; doi:10.1136/bmjopen-2022-061092)
Supplement: Supplementary data [file bmjopen-2022-061092supp002.pdf]

Appendix B: Survey Instrument

Section A – Physician Attire - Ratings

Please rate the doctor for each of the following questions by circling the number that corresponds to your answer.

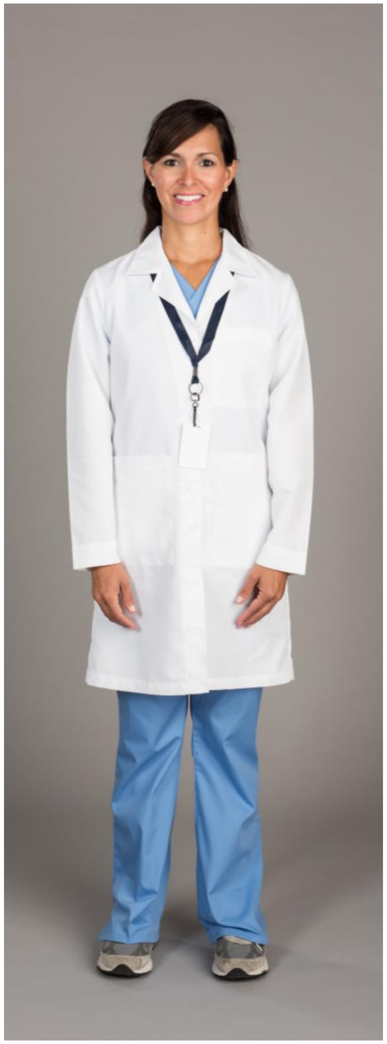	1) How **knowledgeable** does this doctor appear?	1 2 3 4 5 6 7 8 9 10<br>Somewhat Extremely
	2) How **trustworthy** does this doctor appear?	1 2 3 4 5 6 7 8 9 10<br>Somewhat Extremely
	3) How **caring** does this doctor appear?	1 2 3 4 5 6 7 8 9 10<br>Somewhat Extremely
	4) How **approachable** does this doctor appear?	1 2 3 4 5 6 7 8 9 10<br>Somewhat Extremely
	5) How **comfortable** does this doctor make you feel?	1 2 3 4 5 6 7 8 9 10<br>Somewhat Extremely

Houchens N, *et al. BMJ Open* 2022; 12:e061092. doi: 10.1136/bmjopen-2022-061092

D

Section B – Physician Attire - Preferences

Please provide your ONE best answer to each of the following questions

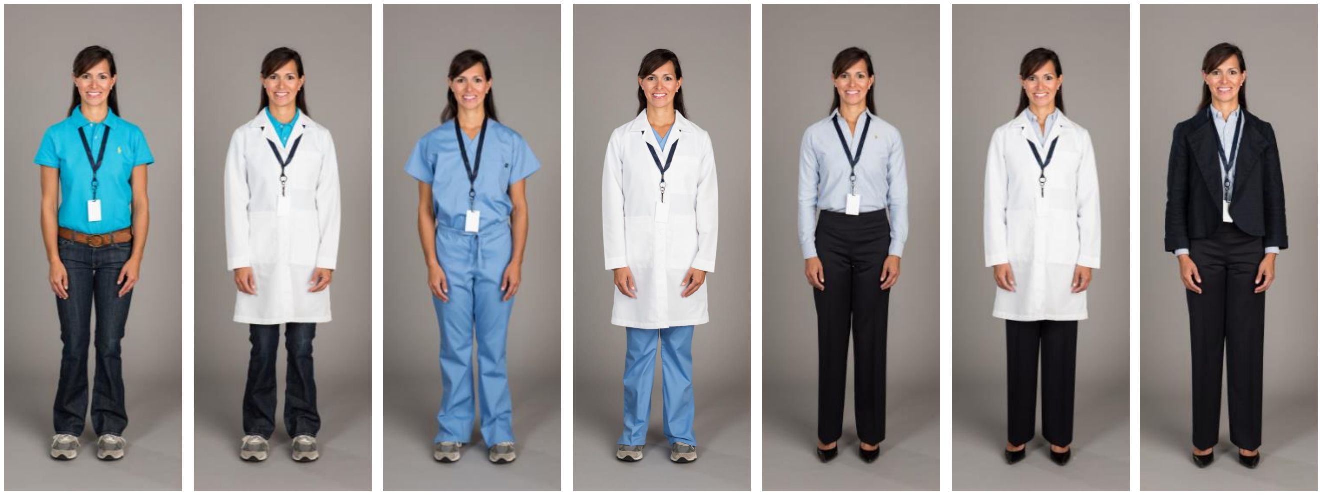

A	B	C	D	E	F	G

6) Which doctor would you prefer for your **primary care doctor**? (Please select only ONE option)

☐	☐	☐	☐	☐	☐	☐
**A**	**B**	**C**	**D**	**E**	**F**	**G**

7) Which doctor would you prefer to see when visiting the **emergency room**? (Please select only ONE option)

☐	☐	☐	☐	☐	☐	☐
**A**	**B**	**C**	**D**	**E**	**F**	**G**

8) Which doctor would you prefer to see when **in the hospital**? (Please select only ONE option)

☐	☐	☐	☐	☐	☐	☐
**A**	**B**	**C**	**D**	**E**	**F**	**G**

9) Which doctor would you prefer for your **surgeon**? (Please select only ONE option)

☐	☐	☐	☐	☐	☐	☐
**A**	**B**	**C**	**D**	**E**	**F**	**G**

10) **Overall**, which clothes do you feel doctors should wear? (Please select only ONE option)

☐	☐	☐	☐	☐	☐	☐
**A**	**B**	**C**	**D**	**E**	**F**	**G**

Houchens N, *et al. BMJ Open* 2022; 12:e061092. doi: 10.1136/bmjopen-2022-061092

D

Section C – General Physician Attire

Please indicate your level of agreement with the following statements by checking ONE box to the left of your answer.

11) How my doctor dresses is important to me.

☐ Strongly Disagree ☐ Disagree ☐ Neither Agree nor Disagree ☐ Agree ☐ Strongly Agree

12) How my doctor dresses influences how happy I am with the care I receive.

☐ Strongly Disagree ☐ Disagree ☐ Neither Agree nor Disagree ☐ Agree ☐ Strongly Agree

13) It is appropriate for a doctor to dress casually when seeing patients **over the weekend.**

☐ Strongly Disagree ☐ Disagree ☐ Neither Agree nor Disagree ☐ Agree ☐ Strongly Agree

14) Doctors should wear a white coat when seeing patients **in their office or clinic**.

☐ Strongly Disagree ☐ Disagree ☐ Neither Agree nor Disagree ☐ Agree ☐ Strongly Agree

15) Doctors should wear a white coat when seeing patients in the **emergency room**.

☐ Strongly Disagree ☐ Disagree ☐ Neither Agree nor Disagree ☐ Agree ☐ Strongly Agree

16) Doctors should wear a white coat when seeing patients **in the hospital**.

☐ Strongly Disagree ☐ Disagree ☐ Neither Agree nor Disagree ☐ Agree ☐ Strongly Agree

17) Doctors should always wear a white coat when seeing patients **in any setting**.

☐ Strongly Disagree ☐ Disagree ☐ Neither Agree nor Disagree ☐ Agree ☐ Strongly Agree

Houchens N, *et al. BMJ Open* 2022; 12:e061092. doi: 10.1136/bmjopen-2022-061092

Section D – Demographics

Please remember that all of your answers will be kept confidential.

18) How old are you?

☐ 18-25 ☐ 26-34 ☐ 35-54 ☐ 55-64 ☐ 65 or older

19) What is your gender?

☐ Male ☐ Female

20) What is the highest level of education you have completed?

☐ Less than High School ☐ High School ☐ Some College ☐ College ☐ Graduate Degree

21) What is your race?

☐ American Indian/Alaska Native ☐ Asian ☐ Native Hawaiian or Other Pacific Islander
☐ Black or African American ☐ White ☐ Hispanic
☐ Other (Please specify) ________________________________

22) How many different doctors have you seen in the past year?

☐ 0 ☐ 1 ☐ 2 ☐ 3 ☐ 4 ☐ 5 ☐ 6 or more

Thank you for taking the time to fill out our survey.
Your input is greatly appreciated.

Houchens N, *et al. BMJ Open* 2022; 12:e061092. doi: 10.1136/bmjopen-2022-061092
