# Supplementary material for: International patient preferences for physician attire: results from cross-sectional studies in four countries across three continents
Source: BMJ Open. 2022 Oct 3;12(10):e061092. doi: 10.1136/bmjopen-2022-061092 (PMC9535197; doi:10.1136/bmjopen-2022-061092)
Supplement: Supplementary data [file bmjopen-2022-061092supp003.pdf]

Appendix C. **Composite ratings of physician attire by domain**

Attire	Domain	Italy			Japan			Switzerland			United States		
		n	Mean	SD	n	Mean	SD	n	Mean	SD	n	Mean	SD
Casual	knowledgeable	137	5.2	2.5	285	5.3	2.4	118	5.6	2.4	752	5.4	2.7
	trustworthy	136	5.5	2.6	286	5.4	2.4	117	6.0	2.4	752	6.0	2.7
	caring	134	6.2	2.4	286	6.2	2.3	119	6.4	2.4	751	6.4	2.6
	approachable	137	6.7	2.3	286	6.5	2.3	119	7.1	2.2	752	6.7	2.6
	comfort	138	6.4	2.8	286	5.8	2.4	117	6.4	2.4	754	6.3	2.8
	mean score	133	6.0	2.3	285	5.8	2.2	115	6.3	2.2	748	6.2	2.5
Casual with white coat	knowledgeable	133	6.3	2.1	288	6.7	2.1	125	6.1	2.4	759	7.2	2.2
	trustworthy	133	6.5	2.1	288	6.8	2.1	124	6.5	2.4	757	7.4	2.2
	caring	133	7.1	2.0	288	7.3	1.9	122	6.6	2.5	759	7.5	2.1
	approachable	133	7.4	1.9	288	7.5	1.9	124	7.1	2.4	764	7.7	2.1
	comfort	133	7.2	2.0	288	7.1	2.1	123	6.5	2.5	759	7.5	2.2
	mean score	133	6.9	1.8	288	7.1	1.8	121	6.6	2.3	747	7.4	2.0
Scrubs	knowledgeable	136	6.2	2.4	283	6.3	2.1	114	6.8	2.0	747	7.0	2.3
	trustworthy	135	6.4	2.3	283	6.5	2.1	116	7.2	2.0	747	7.3	2.2
	caring	134	6.9	2.2	283	7.0	1.9	115	7.0	2.0	746	7.5	2.1
	approachable	136	7.2	2.1	283	7.2	1.8	115	7.4	1.7	749	7.7	2.1
	comfort	136	7.1	2.4	283	6.8	2.0	114	7.1	2.1	749	7.5	2.3
	mean score	134	6.8	2.1	283	6.8	1.8	113	7.1	1.7	742	7.4	2.0
Scrubs with white coat	knowledgeable	126	6.7	2.2	288	6.1	2.0	122	7.1	2.0	761	7.5	2.1
	trustworthy	128	6.9	2.3	290	6.2	2.0	122	7.5	2.1	759	7.6	2.1
	caring	126	7.1	2.3	290	6.8	2.0	121	7.4	2.0	757	7.6	2.1
	approachable	127	7.4	2.0	290	7.2	2.0	120	7.8	1.9	761	7.8	2.1
	comfort	128	7.3	2.2	290	6.6	2.1	121	7.5	1.9	760	7.7	2.2
	mean score	125	7.1	2.0	288	6.6	1.8	120	7.5	1.7	753	7.6	2.0
Formal	knowledgeable	137	5.6	2.4	286	5.5	2.3	121	5.6	2.4	759	7.4	2.1
	trustworthy	137	5.7	2.4	285	5.5	2.3	121	6.0	2.3	759	7.5	2.1

	caring	136	6.1	2.5	286	6.1	2.1	119	5.8	2.6	756	7.5	2.1
	approachable	137	6.5	2.3	286	6.3	2.2	121	6.0	2.6	763	7.7	2.1
	comfort	137	6.1	2.5	286	5.8	2.3	121	5.7	2.5	761	7.5	2.2
	mean score	136	6.0	2.2	285	5.9	2.1	119	5.8	2.3	754	7.5	2.0
Formal with white coat	knowledgeable	131	7.2	2.1	284	6.6	1.9	102	7.4	2.0	764	8.2	1.9
	trustworthy	130	7.4	2.0	284	6.7	1.9	101	7.4	2.0	761	8.2	1.9
	caring	131	7.6	1.9	284	7.4	1.7	101	7.1	2.1	759	8.0	1.9
	approachable	131	7.8	1.8	284	7.4	1.8	102	7.2	2.1	758	8.1	1.9
	comfort	130	7.7	1.8	284	7.0	1.8	101	7.0	2.3	758	8.1	2.0
	mean score	130	7.5	1.8	284	7.0	1.6	101	7.2	1.9	754	8.1	1.8
Business suit	knowledgeable	131	5.5	2.6	295	5.3	2.2	110	5.2	2.5	755	7.4	2.3
	trustworthy	129	5.7	2.5	295	5.4	2.2	109	5.4	2.5	755	7.3	2.3
	caring	130	5.6	2.5	296	5.8	2.2	110	5.0	2.4	754	7.1	2.4
	approachable	128	5.8	2.6	296	5.8	2.3	110	5.4	2.5	753	7.2	2.4
	comfort	131	5.5	2.8	295	5.4	2.3	109	5.2	2.5	755	7.0	2.5
	mean score	128	5.6	2.4	295	5.5	2.1	108	5.2	2.2	751	7.2	2.2

Appendix D. Comparisons of patient preferences for physician attire by type of attire between countries

	Casual				Casual + White Coat				Scrubs				Scrubs + White Coat			
Location Comparison	Mean difference	Simultaneous 95% confidence limits		sig	Mean difference	Simultaneous 95% confidence limits		sig	Mean difference	Simultaneous 95% confidence limits		sig	Mean difference	Simultaneous 95% confidence limits		sig
Italy-Japan	0.2049	-0.4354	0.8452		-0.1829	-0.714	0.3481		0.0028	-0.5287	0.5343		0.46551	-0.0631	0.99412	
Italy-US	-0.1454	-0.7192	0.4284		-0.5303	-1.007	-0.0535	***	-0.6422	-1.118	-0.1665	***	-0.58969	-1.06635	-0.11303	***
Italy-Swiss	-0.3137	-1.0902	0.4627		0.3128	-0.3236	0.9492		-0.3083	-0.9557	0.3391		-0.43477	-1.06551	0.19598	
Japan-US	-0.3503	-0.7748	0.0742		-0.3473	-0.6987	0.004		-0.645	-0.9992	-0.2909	***	-1.0552	-1.39714	-0.71326	***
Japan-Swiss	-0.5186	-1.1923	0.155		0.4957	-0.0531	1.0445		-0.3111	-0.8751	0.253		-0.90028	-1.43652	-0.36404	***
US-Swiss	-0.1683	-0.7791	0.4424		0.843	0.3466	1.3394	***	0.3339	-0.1779	0.8458		0.15492	-0.33018	0.64003	

	Formal				Formal + White Coat				Suit			
Location Comparison	Mean difference	Simultaneous 95% confidence limits		sig	Mean difference	Simultaneous 95% confidence limits		sig	Mean difference	Simultaneous 95% confidence limits		sig
Italy-Japan	0.1275	-0.4215	0.6765		0.53538	0.05998	1.01079	***	0.101	-0.4955	0.6975	
Italy-US	-1.553	-2.0438	-1.0622	***	-0.57297	-0.99932	-0.14662	***	-1.5514	-2.0903	-1.0125	***
Italy-Swiss	0.1381	-0.5231	0.7994		0.3136	-0.28188	0.90908		0.4027	-0.3337	1.139	
Japan-US	-1.6805	-2.0468	-1.3142	***	-1.10836	-1.42093	-0.79579	***	-1.6523	-2.0396	-1.2651	***
Japan-Swiss	0.0106	-0.5643	0.5856		-0.22178	-0.7419	0.29834		0.3017	-0.3321	0.9355	
US-Swiss	1.6911	1.1715	2.2108	***	0.88657	0.41088	1.36227	***	1.954	1.3741	2.534	***

Sig, ***: Statistically significant

Appendix E. **Comparisons of respondent opinions regarding importance, influence, and appropriateness of physician attire and white coats between countries**

	Important				Influence				Casual weekend				White coat office				White coat ER				White coat hospital				White coat any setting			
Location Comparison	Mean difference	Simultaneous 95% confidence limits		sig	Mean difference	Simultaneous 95% confidence limits		sig	Mean difference	Simultaneous 95% confidence limits		sig	Mean difference	Simultaneous 95% confidence limits		sig	Mean difference	Simultaneous 95% confidence limits		sig	Mean difference	Simultaneous 95% confidence limits		sig	Mean difference	Simultaneous 95% confidence limits		sig
Italy-Japan	0.03935	-0.06317	0.14187		-0.29709	-0.40417	-0.19002	***	0.57316	0.46985	0.67648	***	0.33013	0.23509	0.42516	***	1.01157	0.91526	1.10788	***	0.599	0.51144	0.68657	***	0.57579	0.47487	0.67671	***
Italy-US	0.05486	-0.03705	0.14677		-0.24249	-0.33851	-0.14646	***	-0.12125	-0.21387	-0.02864	***	0.38806	0.30286	0.47326	***	0.72743	0.64117	0.81369	***	0.53173	0.45328	0.61019	***	0.43908	0.34865	0.52952	***
Italy-Swiss	0.49847	0.37459	0.62235	***	0.44933	0.32	0.57867	***	-0.22814	-0.35336	-0.10292	***	0.65738	0.5425	0.77226	***	0.04456	-0.072	0.16113		0.27093	0.16489	0.37697	***	0.73863	0.61654	0.86072	***
Japan-US	0.01551	-0.05221	0.08323		0.05461	-0.01601	0.12522		-0.69442	-0.76271	-0.62613	***	0.05793	-0.00488	0.12075		-0.28414	-0.34798	-0.22031	***	-0.06727	-0.12529	-0.00925	***	-0.1367	-0.20354	-0.06986	***
Japan-Swiss	0.45912	0.35195	0.56629	***	0.74643	0.63466	0.8582	***	-0.8013	-0.90977	-0.69283	***	0.32726	0.22784	0.42667	***	-0.96701	-1.06811	-0.8659	***	-0.32808	-0.42003	-0.23612	***	0.16284	0.05703	0.26865	***
US-Swiss	0.44361	0.34655	0.54067	***	0.69182	0.59059	0.79306	***	-0.10689	-0.20522	-0.00856	***	0.26933	0.17926	0.35939	***	-0.68287	-0.77444	-0.59129	***	-0.26081	-0.34414	-0.17748	***	0.29954	0.20369	0.3954	***

Sig, ***: Statistically significant

Important: How my doctor dresses is important to me.

Influence: How my doctor dresses influences how happy I am with the care I receive.

Casual weekend: It is appropriate for a doctor to dress casually when seeing patients over the weekend.

White coat office: Doctors should wear a white coat when seeing patients in their office.

White coat ER: Doctors should wear a white coat when seeing patients in the emergency room.

White coat hospital: Doctors should wear a white coat when seeing patients in the hospital.

White coat any setting: Doctors should always wear a white coat when seeing patients in any setting.

Appendix F. **Composite scores by respondent gender**

**Attire**	**Italy**			**Japan**			**Switzerland**			**United States**		
	**Male**	**Female**	**P**	**Male**	**Female**	**P**	**Male**	**Female**	**P**	**Male**	**Female**	**P**
Casual	6.0	6.1	0.77	6.0	5.6	0.13	6.5	6.0	0.21	6.3	6.0	0.10
Casual with white coat	7.0	6.9	0.85	7.2	7.0	0.40	6.5	6.6	0.90	7.3	7.5	0.16
Scrubs	6.5	6.9	0.34	6.8	6.8	0.93	7.2	6.9	0.38	7.4	7.5	0.71
Scrubs with white coat	7.3	6.9	0.26	6.5	6.6	0.60	7.5	7.5	0.96	7.6	7.7	0.41
Formal	5.6	6.3	0.09	6.0	5.7	0.28	6.2	5.4	0.04*	7.6	7.4	0.23
Formal with white coat	7.5	7.6	0.73	7.0	7.0	0.77	7.3	7.1	0.55	8.1	8.1	0.94
Business suit	5.5	5.8	0.52	5.6	5.4	0.41	5.1	5.2	0.74	7.1	7.3	0.38

* Statistically significant

Appendix G. **Composite scores by respondent age**

Attire	Italy						Japan					
	**18-25**	**26-34**	**35-54**	**55-64**	**65+**	**P**	**18-25**	**26-34**	**35-54**	**55-64**	**65+**	**P**
Casual	4.8	5.6	6.1	6.4	6.2	0.40	5.4	4.6	5.2	5.4	6.3	0.001*
Casual with white coat	8.1	6.5	6.4	7.1	7.0	0.06	8.0	7.0	6.6	6.7	7.4	0.003*
Scrubs	6.4	7.6	6.5	7.0	6.7	0.63	6.0	5.9	6.6	6.6	7.0	0.07
Scrubs with white coat	7.5	7.4	6.4	7.3	7.5	0.12	7.3	6.9	6.3	6.4	6.7	0.37
Formal	5.7	6.3	5.8	6.0	6.0	0.95	6.1	5.1	5.3	5.5	6.4	0.002*
Formal with white coat	7.9	7.3	7.6	7.3	7.7	0.76	7.5	6.6	6.6	6.8	7.3	0.01*
Business suit	4.7	7.1	5.7	5.3	5.4	0.12	5.0	4.8	5.0	5.2	6.1	<0.001*
	**Switzerland**						**United States**					
	**18-25**	**26-34**	**35-54**	**55-64**	**65+**	**P**	**18-25**	**26-34**	**35-54**	**55-64**	**65+**	**P**
Casual	7.7	6.6	6.3	6.4	5.9	0.72	5.9	6.3	5.8	6.1	6.5	0.09
Casual with white coat	7.2	7.5	6.6	6.1	6.2	0.27	8.0	7.6	7.2	7.3	7.6	0.03*
Scrubs	7.3	6.4	6.9	7.5	7.5	0.35	8.1	7.9	7.2	7.2	7.6	0.01*
Scrubs with white coat	8.5	7.8	7.1	7.5	8.0	0.10	7.9	7.7	7.7	7.5	7.7	0.73

Formal	5.9	5.2	5.8	6.3	5.8	0.52	8.3	7.5	7.2	7.5	7.8	0.003*
Formal with white coat	6.8	6.1	7.3	7.9	6.9	0.20	8.2	7.8	8.0	8.1	8.3	0.15
Business suit	5.8	4.4	5.2	5.4	5.4	0.69	7.1	7.2	7.0	7.1	7.4	0.28

* Statistically significant
