# Supplementary material for: International patient preferences for physician attire: results from cross-sectional studies in four countries across three continents
Source: BMJ Open. 2022 Oct 3;12(10):e061092. doi: 10.1136/bmjopen-2022-061092 (PMC9535197; doi:10.1136/bmjopen-2022-061092)
Supplement: Author's manuscript [file bmjopen-2022-061092.draft_revisions.pdf]

BMJ Open is committed to open peer review. As part of this commitment we make the peer review history of every article we publish publicly available.

When an article is published we post the peer reviewers' comments and the authors' responses online. We also post the versions of the paper that were used during peer review. These are the versions that the peer review comments apply to.

The versions of the paper that follow are the versions that were submitted during the peer review process. They are not the versions of record or the final published versions. They should not be cited or distributed as the published version of this manuscript.

BMJ Open is an open access journal and the full, final, typeset and author-corrected version of record of the manuscript is available on our site with no access controls, subscription charges or pay-per-view fees (http://bmjopen.bmj.com).

If you have any questions on BMJ Open's open peer review process please email info.bmjopen@bmj.com

BMJ Open

International Patient Preferences for Physician Attire: Results from Cross-Sectional Studies in Four Countries Across Three Continents

Journal:	*BMJ Open*
Manuscript ID	bmjopen-2022-061092
Article Type:	Original research
Date Submitted by the Author:	22-Jan-2022

Complete List of Authors:	Houchens, Nathan; VA Ann Arbor Healthcare System; University of Michigan, Department of Medicine<br>Saint, Sanjay; VA Ann Arbor Healthcare System; University of Michigan, Department of Medicine<br>Petrilli, Christopher; NYU Langone Health<br>Kuhn, Latoya; VA Ann Arbor Healthcare System; University of Michigan, Department of Medicine<br>Ratz, David; VA Ann Arbor Healthcare System<br>De Lott, Lindsey; W K Kellogg Eye Center<br>Zollinger, Marc; Psychiatric University Hospital Zurich Department of Social and General Psychiatry Zurich West<br>Sax, Hugo; Inselspital University Hospital Bern, Department of Infectious Diseases<br>Kamata, Kazuhiro ; Niigata University Faculty of Medicine Graduate School of Medical and Dental Science, Department of Pediatrics; Fukushima Medical University Aizu Medical Center, Department of General Internal Medicine<br>Kuriyama, Akira; Kurashiki Central Hospital Emergency and Critical Care Center<br>Tokuda, Yasuharu; Muribushi Project for Okinawa Residency Programs, Department of Medicine<br>Fumagalli, Carlo; University of Florence, Department of Experimental and Clinical Medicine<br>Virgili, Gianni ; University of Florence, Department of Neurosciences, Psychology, Drug Research and Child Health (NEUROFARBA); Queen's University Belfast, Centre for Public Health<br>Fumagalli, Stefano; University of Florence, Department of Experimental and Clinical Medicine<br>Chopra, Vineet; University of Colorado, Department of Medicine

Keywords:	GENERAL MEDICINE (see Internal Medicine), Health policy < HEALTH SERVICES ADMINISTRATION & MANAGEMENT, Protocols & guidelines < HEALTH SERVICES ADMINISTRATION & MANAGEMENT

I, the Submitting Author has the right to grant and does grant on behalf of all authors of the Work (as defined in the below author licence), an exclusive licence and/or a non-exclusive licence for contributions from authors who are: i) UK Crown employees; ii) where BMJ has agreed a CC-BY licence shall apply, and/or iii) in accordance with the terms applicable for US Federal Government officers or employees acting as part of their official duties; on a worldwide, perpetual, irrevocable, royalty-free basis to BMJ Publishing Group Ltd ("BMJ") its licensees and where the relevant Journal is co-owned by BMJ to the co-owners of the Journal, to publish the Work in this journal and any other BMJ products and to exploit all rights, as set out in our licence.

The Submitting Author accepts and understands that any supply made under these terms is made by BMJ to the Submitting Author unless you are acting as an employee on behalf of your employer or a postgraduate student of an affiliated institution which is paying any applicable article publishing charge ("APC") for Open Access articles. Where the Submitting Author wishes to make the Work available on an Open Access basis (and intends to pay the relevant APC), the terms of reuse of such Open Access shall be governed by a Creative Commons licence – details of these licences and which Creative Commons licence will apply to this Work are set out in our licence referred to above.

Other than as permitted in any relevant BMJ Author's Self Archiving Policies, I confirm this Work has not been accepted for publication elsewhere, is not being considered for publication elsewhere and does not duplicate material already published. I confirm all authors consent to publication of this Work and authorise the granting of this licence.

For peer review only

International Patient Preferences for Physician Attire: Results from Cross-Sectional Studies in Four Countries Across Three Continents

Nathan Houchens, MD[1,2]

Sanjay Saint, MD, MPH[1,2]

Christopher M. Petrilli, MD[3]

Latoya Kuhn, MPH[1,2]

David Ratz, MS[1]

Lindsey De Lott, MD, MS[4]

Marc Zollinger, MSc[5]

Hugo Sax, MD[6]

Kazuhiro Kamata, MD[7,8]

Akira Kuriyama, MD, MPH, PhD[9]

Yasuharu Tokuda, MD, MPH[10]

Carlo Fumagalli, MD[11]

Gianni Virgili, MD[12,13]

Stefano Fumagalli, MD, PhD[11]

Vineet Chopra, MD, MSc[14]

1 Medicine Service, Veterans Affairs Ann Arbor Healthcare System, Ann Arbor, Michigan, USA

2 Division of Hospital Medicine, Department of Medicine, University of Michigan, Ann Arbor, Michigan, USA

For peer review only

For peer review only - http://bmjopen.bmj.com/site/about/guidelines.xhtml

3 Division of General Internal Medicine and Clinical Innovation, Department of Medicine, NYU Langone Health, New York, New York, USA

4 Kellogg Eye Center, University of Michigan, Ann Arbor, Michigan, USA

5 University Hospital of Psychiatry Zurich (PUK), Zurich, Switzerland

6 Department of Infectious Diseases, Bern University Hospital and University of Bern, Bern, Switzerland

7 Department of Pediatrics, Niigata University Graduate School of Medical and Dental Sciences, Niigata, Japan

8 Department of General Internal Medicine, Aizu Medical Center, Fukushima Medical University, Fukushima, Japan

9 Emergency and Critical Care Center, Kurashiki Central Hospital, Okayama, Japan

10 Department of Medicine, Muribushi Project for Okinawa Residency Programs, Okinawa, Japan

11 Department of Experimental and Clinical Medicine, Geriatric Intensive Care Unit, University of Florence, Florence, Italy

12 Department of Neurosciences, Psychology, Drug Research and Child Health (NEUROFARBA), University of Florence and AOU, Florence, Italy

13 Centre for Public Health, Queen's University Belfast, Belfast, United Kingdom

14 Division of Hospital Medicine, Department of Medicine, University of Colorado, Denver, Colorado, USA

RUNNING TITLE: International Patient Preferences for Physician Attire

For peer review only

For peer review only - http://bmjopen.bmj.com/site/about/guidelines.xhtml

WORD COUNT: 3,850

CORRESPONDING AUTHOR:

Nathan Houchens, MD

Veterans Affairs Ann Arbor Healthcare System and University of Michigan

2215 Fuller Road, Mail Code 111

Ann Arbor, Michigan 48105

Email: nathanho@med.umich.edu

Phone: 734-845-5922

Fax: 734-845-5944

For peer review only

ABSTRACT

Objective: The patient-physician relationship impacts patients' experiences and health outcomes. Physician attire is a form of nonverbal communication that influences this relationship. Prior studies examining attire preferences suffered from heterogenous measurement and limited context. We thus performed a multi-center, cross-sectional study using a standardized survey instrument to compare patient preferences for physician dress in international settings.

Setting: 20 hospitals and healthcare practices in Italy, Japan, Switzerland, and the United States.

Participants: Convenience sample of 9,171 adult patients receiving care in academic hospitals, general medicine clinics, specialty clinics, and ophthalmology practices.

Primary and secondary outcome measures: The survey was randomized and included photographs of a male or female physician dressed in assorted forms of attire. The primary outcome measure was attire preference, comprised of composite ratings across five domains: how knowledgeable, trustworthy, caring, and approachable the physician appeared, and how comfortable the respondent felt. Secondary outcome measures included variation in preferences by country, physician type, and respondent characteristics.

Results: The highest rated forms of attire differed by country, although each most preferred attire with white coat. Low ratings were conferred on attire extremes (casual and business suit). Preferences were more uniform for certain physician types. For example, among all respondents, scrubs garnered the highest rating for emergency department physicians (44.2%) and surgeons (42.4%). However, attire preferences diverged for primary care and hospital physicians. All types of formal attire were more strongly preferred in the United States than elsewhere. Respondent age influenced preferences in Japan and the United States only.

For peer review only

For peer review only - http://bmjopen.bmj.com/site/about/guidelines.xhtml

Conclusions: Patients across a myriad of geographies, settings, and demographics harbor specific preferences for physician attire. Some preferences are nearly universal, whereas others vary substantially. As a one-size-fits-all dress policy is unlikely to reflect patient desires and expectations, a tailored approach should be sought that attempts to match attire to clinical context.

Keywords: Physician attire, dress, clothing, uniform, patient preferences, patient-physician relationship, nonverbal communication

ARTICLE SUMMARY

Strengths and Limitations of This Study

- With over 9,000 participants, this is the largest international study examining opinions on physician dress to date.
- Use of a standardized survey instrument allowed direct comparisons across diverse geographic regions, populations, physician types, and clinical contexts.
- Robust and careful survey design, including randomization and constant photographic features, mitigated bias and confounding.
- Comparative over-representation of the United States and convenience sampling may have contributed to disproportionate representation.
- The survey instrument used pre-defined Likert scales, which may not accurately reflect nuanced patient opinions, and which do not capture other elements of patient-physician interactions.

For peer review only - http://bmjopen.bmj.com/site/about/guidelines.xhtml

INTRODUCTION

Successful patient-physician relationships are founded on mutual respect, trust, confidence, and care. The strength of these connections can directly impact patients' experiences with healthcare, satisfaction, and important health outcomes such as adherence to treatment recommendations,[1,2] 30-day readmissions,[3] and mortality.[4] Patient-physician interactions are complex and dependent on multiple factors including social definitions and cultural norms. To ensure the highest quality care, it is essential to identify techniques that physicians may use to establish and maintain strong relationships with their unique individual patients while recognizing the influence of sociocultural context. From initial introductions, physicians employ verbal and nonverbal communication to form impressions and cultivate partnerships with their patients.[5]

The clothing worn by a physician is one form of nonverbal communication that may influence the patient-physician relationship. Physician attire is an important element in establishing patient confidence and trust,[6] enhancing patient comfort when discussing personal problems,[7-9] and shaping patient perceptions of physician professionalism,[6] intelligence,[10] and empathy.[11] Most prior scholarship has focused on a single geographic region, country, or clinical context (e.g., primary care clinic, hospital setting)[12-15] and has not considered the relative impacts of different physician specialties, contexts of care, geography, and patient factors such as age, education, and gender. Additionally, heterogeneity among prior studies, such as different sampling methodology and survey instruments, has made comparisons across different studies challenging.

The objective of this international, multi-center, cross-sectional study was to use a structured survey instrument to examine patient preferences for physician attire in different regions, countries, and continents. The survey instrument allowed direct comparisons among a

For peer review only - http://bmjopen.bmj.com/site/about/guidelines.xhtml

variety of cultures and contexts, thereby mitigating the heterogeneity of prior studies.[16-18] We report comparisons of data from five primary cross-sectional survey research studies conducted in Italy, Japan,[19] Switzerland,[20] and the United States.[21,22] Our aim was to identify common themes and differences of patient expectations for physician dress so that we may tailor attire and thus elevate the patient experience and optimize health outcomes.

METHODS

Study Design and Participants

We performed a survey-based study using a convenience sample of patients in 20 hospitals and healthcare practices in Italy, Japan, Switzerland, and the United States. These sites were selected based on our research networks and availability of clinicians who would serve as leads in their respective institutions. Sites included academic hospitals (general medicine wards, intensive care units), general medicine ambulatory clinics, specialty ambulatory clinics (dermatology, infectious disease, neurology, orthopedic surgery), and ophthalmology practices (**Table 1**). Data collection took place between June 2015 and October 2017.

At each participating healthcare location, the research team printed and randomly administered a survey instrument, targeting representative adult patients who were receiving clinical care at one of those sites. Participants were presented with a paper-based instrument of 22 questions that included photographs of either a male or female physician wearing various forms of attire and asked to rate their preferences. Respondents could request assistance with form completion from persons accompanying them.

All participants provided informed verbal consent. No identifying information was collected from participants that completed the study. Institutional permission for recruitment and

For peer review only - http://bmjopen.bmj.com/site/about/guidelines.xhtml

data collection was obtained from each site. The country-specific ethical review committees that reviewed and approved or deemed this project exempt from regulation were the University of Michigan Institutional Review Board (United States, HUM00085305); the Cantonal Ethics Review Board of Zurich, based on the Swiss law on research on humans (Switzerland, No. 60-2015); the ethics committee for Tokyo Joto Hospital (Japan, No. 2015-0001); and the ethics committee for Careggi University Hospital, according to the Declaration of Helsinki (Italy, CE 7123).

Procedures

The 22-item survey instrument was developed following a systematic review of the literature that examined the role of physician attire on the patient experience.[23] The survey instrument was developed and piloted by a multidisciplinary team to gather feedback and refine photographs, questions, rating scale, presentation order, and randomization scheme. Questions were translated into different languages for each country by interpreters at each site: Italian for Italy, Japanese for Japan, German for Switzerland (since the Swiss survey was conducted in Zurich), and English for the United States.

Each question referenced particular preferences and opinions of respondents in relation to photographs of medical providers wearing seven unique forms of attire. The forms of dress presented included: casual, casual with white coat, scrubs, scrubs with white coat, formal, formal with white coat, and business suit. Photographs were taken with attention paid to achieving constant physician facial expressions as well as consistent visual cues such as lighting, background, and pose. Photographs used at all study sites were identical with the following exceptions: In Switzerland, photographs of physicians in medical attire contextually appropriate

For peer review only

to the Swiss health system (i.e., white scrubs instead of blue scrubs) were used. All other photographic elements including physician models and other forms of attire were unchanged. In Japan, photographs of physicians of Japanese descent with slightly modified attire were used (**Appendix A**).

Each survey instrument had four sections. The first section showed a photograph of either a male or female physician wearing one of the seven unique forms of attire. To avoid biases such as anchoring, priming, order response, and gender conformity, 14 different versions of the survey instrument were created. The gender and attire of the first photograph seen by each respondent were randomized; all other sections of the survey were identical (**Appendix B**).

Measurements

Respondents were first asked to rate the standalone, randomized physician photograph using a 1 to 10 scale across five domains (i.e., how knowledgeable, trustworthy, caring, and approachable the physician appeared, and how comfortable the physician's appearance made the respondent feel), with a score of 10 representing the highest rating. Respondents were subsequently given seven photographs of the same physician wearing various forms of attire. Questions were asked regarding preference of attire in varied clinical settings (i.e., primary care, emergency department, hospital, surgery) and overall preference. To identify the influence of and respondent preferences for physician dress and white coats, a Likert scale ranging from 1 (strongly disagree) to 5 (strongly agree) was employed. Preferences for attire by respondent characteristics such as age, gender, education level, nationality, and number of unique physicians seen in the past year were collected. Unanswered questions and those with multiple responses were excluded.

For peer review only - http://bmjopen.bmj.com/site/about/guidelines.xhtml

The primary outcome of attire preference was calculated as the mean composite score of the five individual rating domains (i.e., knowledgeable, trustworthy, caring, approachable, and comfortable), with the highest score representing the most preferred form of attire. We also assessed variation in preferences for physician attire between countries, by physician type and clinical setting, and by respondent characteristics such as age and gender.

Statistical Analysis

Survey data were entered independently and in duplicate by the study teams. Respondents were not required to answer all questions; therefore, the denominator for each question varied. Data were reported as mean and standard deviation (SD) or N and percentage, where appropriate. Differences in the mean composite rating scores between countries were assessed using one-way ANOVA with the Tukey method for pairwise comparisons. Differences in mean composite score within country by sociodemographic factors were assessed using Student's T-tests. Differences between countries with respect to categorical responses were compared using Chi-squared tests. Statistical tests were assessed using p-value <0.05 considered significant. All analyses were performed using SAS V9.4 (SAS Inc, Cary, NC).

Patient and Public Involvement

Patients were not included in the design of the survey instrument, recruitment, or conduct of the study. Patients who participated did so anonymously, and therefore, the study team will be unable to disseminate the results to study participants.

Role of the Funding Source

For peer review only - http://bmjopen.bmj.com/site/about/guidelines.xhtml

This work was partially supported by a Swiss National Science Foundation grant (32003B_149474; PI, HS). Several investigators (SS, HS, MZ, VC, LD) received extramural funding for salary support. All authors had full access to all the data in the study and accept responsibility for the decision to submit for publication.

RESULTS

Characteristics of Study Sites and Participants

A total of 9,171 patients completed the survey instrument in outpatient and inpatient healthcare settings within a total of 20 hospitals or practices, 13 distinct geographic regions, 4 countries, and 3 continents. Patients aged 65 years or older comprised 36.0% of all respondents with substantial age variation across countries. For instance, those 65 years or older represented 48.5% of respondents in Japan, 35.6% in the United States, 27.8% in Italy, and 16.7% in Switzerland. Among all respondents, 44.9% were female, 39.6% had a college or graduate degree, and 26.6% had seen 6 or more physicians in the previous year. Characteristics of study sites are found in **Table 1**, and sociodemographic characteristics of respondents are described in **Table 2**.

Ratings of Attire Types by Country

Responses regarding patient preferences for physician attire varied by country. Formal attire with white coat received the highest ratings from respondents in Italy and the United States with mean composite scores of 7.5 (SD 1.8) and 8.1 (SD 1.8), respectively. Conversely, scrubs with white coat received the highest ratings in Switzerland (mean composite score of 7.5 [SD 1.7]) and casual attire with white coat in Japan (mean composite score of 7.1 [SD 1.8]). The forms of attire

For peer review only

For peer review only - http://bmjopen.bmj.com/site/about/guidelines.xhtml

that received the lowest mean composite ratings were business suit in Italy, Japan, and Switzerland with mean composite scores of 5.6 (SD 2.4), 5.5 (SD 2.1), and 5.2 (SD 2.2), respectively and casual attire in the United States with a mean composite score of 6.2 (SD 2.5). Ratings of different forms of attire by country are found in **Figure 1** and ratings of physician attire by domain are found in **Appendix C**.

Comparisons of Patient Preferences Between Countries

Preferences for Physician Attire by Type of Attire

Similarities between countries when comparing preferences for different types of physician attire were observed. For instance, there was complete concordance for all types of attire between the European countries of Italy and Switzerland. There was near complete concordance when comparing Italy and Japan, with the only statistically significant difference of Italy more strongly preferring formal attire with white coat compared with Japan (mean composite rating difference 0.54, simultaneous 95% confidence limits 0.06 to 1.01). Similarly, there was near complete concordance when comparing Switzerland and Japan, with the only significant difference of Switzerland more strongly preferring scrubs with white coat compared with Japan (mean composite rating difference 0.90, simultaneous 95% confidence limits 0.36 to 1.44). Among all types of attire, the form with the most concordance across countries was casual attire, with no between-country differences identified.

Just as ratings for physician attire varied by country, preferences for specific forms of attire also differed. For instance, the United States significantly more strongly preferred both forms of scrubs-based attire when compared with Italy and Japan, but not when compared with Switzerland. Additionally, the United States significantly more strongly preferred all forms of

For peer review only

formal attire (i.e., formal attire with or without white coat and business suit) when compared with the other countries. These results are summarized in **Appendix D**.

Preferences for Physician Attire by Type of Physician

Photographs of either a male or female physician in seven different forms of attire (**Appendix B**) were shown, and respondents were asked to select which attire they preferred for different physician types. With respect to primary care physicians, respondents had varying preferences for attire. The highest rated forms in each country were formal attire with white coat in Italy (31.6%) and the United States (46.8%), casual attire with white coat in Japan (34.1%), and casual attire in Switzerland (24.4%). Heterogeneity in patient preferences was particularly noted in Switzerland with nearly equal preference given to casual attire, casual attire with white coat, and formal attire with white coat. The highest rated form of attire across all respondents was formal attire with white coat (40.1%).

With respect to hospital-based physicians, preferences again diverged. The highest rated forms in each country were scrubs with white coat in Italy (43.8%) and Switzerland (35.0%), casual attire with white coat in Japan (34.0%), and formal attire with white coat in the United States (37.6%). The highest rated form of attire across all respondents was formal attire with white coat (32.8%).

With respect to both emergency department physicians and surgeons, preferences were more uniform. Among all respondents across all countries, the most preferred form of attire was scrubs (44.2% for emergency department physicians, 42.4% for surgeons) followed by scrubs with white coat (30.4% for emergency department physicians, 25.4% for surgeons).

For peer review only - http://bmjopen.bmj.com/site/about/guidelines.xhtml

With respect to the most preferred form of attire overall, differences between countries were noted. The top forms of attire in each country were scrubs with white coat in Italy (41.7%) and Switzerland (31.5%) and formal attire with white coat in Japan (35.3%) and the United States (45.7%). The highest rated form of attire across all respondents was formal attire with white coat (38.6%). **Table 3** shows preferred physician attire by physician type and clinical care setting.

Importance, Impact, and Appropriateness of Physician Attire and White Coats

Respondent opinions were sought using a Likert scale in which a score of 1 indicated “strongly disagree” and 5 “strongly agree.” In response to the prompt “how my doctor dresses is important to me,” mean scores were similar for Italy (3.55), Japan (3.51), and the United States (3.49) and lower for Switzerland (3.05) ($p<0.05$ for all 3 pairwise comparisons). In response to the prompt “how my doctor dresses influences how happy I am with the care I receive,” mean scores for Italy were 2.92, Japan 3.22, Switzerland 2.47, and the United States 3.17 ($p<0.05$ for all pairwise comparisons except for Japan-United States). In response to the prompt “it is appropriate for a doctor to dress casually when seeing patients over the weekend,” all countries differed with mean scores for Italy of 3.15, Japan 2.57, Switzerland 3.37, and the United States 3.27 ($p<0.05$ for all 6 pairwise comparisons).

With respect to perceptions of whether white coats should be worn by physicians in various settings, differences emerged. When considering whether physicians should wear a white coat when seeing patients in their office, mean scores for Italy were 3.92, Japan 3.59, Switzerland 3.27, and the United States 3.53 ($p<0.05$ for all pairwise comparisons except for Japan-United States). When asked if physicians should wear a white coat in the emergency

For peer review only - http://bmjopen.bmj.com/site/about/guidelines.xhtml

department, mean scores for Italy were 4.06, Japan 3.05, Switzerland 4.02, and the United States 3.34 ($p<0.05$ for all pairwise comparisons except for Italy-Switzerland). When asked if physicians should wear a white coat in the hospital, all countries differed with mean scores for Italy of 4.16, Japan 3.57, Switzerland 3.89, and the United States 3.63 ($p<0.05$ for all 6 pairwise comparisons). In response to the prompt "doctors should always wear a white coat when seeing patients in any setting," all countries differed with mean scores for Italy of 3.56, Japan 2.99, Switzerland 2.82, and the United States 3.12 ($p<0.05$ for all 6 pairwise comparisons). These results are summarized in **Table 4** and **Appendix E**.

Comparisons of Patient Preferences Within Countries

Similarities and differences were identified when comparing preferences within countries based on respondent sociodemographic characteristics. When examining respondent gender, men and women rated different types of physician attire similarly within their respective countries. The only significant difference was that men rated formal attire more highly than women in Switzerland (male composite score 6.2, female composite score 5.4, p=0.04) (**Appendix F**). When comparing respondents aged 65 years and older with those less than 65 years, there were no significant differences in composite scores for all types of physician attire in both Italy and Switzerland. In contrast, when compared with the younger cohort, respondents aged 65 years and older rated casual attire, formal attire, formal attire with white coat, and business suit more highly in both Japan and the United States. When compared with the younger cohort, respondents aged 65 years and older rated casual attire with white coat and scrubs more highly in Japan, a finding that was not significant in the United States (**Appendix G**). There was no

For peer review only - http://bmjopen.bmj.com/site/about/guidelines.xhtml

association between respondent preferences for physician attire and number of physicians seen in the prior year.

DISCUSSION

In this international, multi-center, cross-sectional study, we report preferences of 9,171 patients for physician attire across a variety of geographic regions, clinical contexts, physician types, and patient sociodemographic characteristics. We found that the highest rated form of physician attire differed across countries, but that all most strongly preferred a white coat with any attire. Respondents from the United States more strongly preferred all types of formal attire compared with those from Italy, Japan, and Switzerland. All countries more strongly preferred scrubs-based attire for emergency department physicians and surgeons. Taken together, these findings suggest that how a physician dresses has importance that varies around the world.

Our study adds to the existing literature by demonstrating that patients harbor expectations of how their physicians dress, and these expectations depend on sociocultural norms, context, and patient factors. In some clinical care contexts, preferences vary substantially. In others, they are nearly universal such as those for emergency department physicians and surgeons wearing scrubs-based attire. With some exceptions, patients tended to dislike extremes in attire such as casual or business suit. Finally, it was very common for patients to prefer their physicians wear a white coat, a historically traditional aspect of the physician's uniform and what is often considered a symbol of the profession.[24] This was particularly evident when patient preferences for the underlying form of attire were split (e.g., primary care and hospital physicians).

For peer review only - http://bmjopen.bmj.com/site/about/guidelines.xhtml

Other studies exploring patient perceptions for physician attire have yielded a diverse and often conflicting array of findings, most of which are complicated by different measurement tools and outcomes. Consistent with our results, numerous studies across continents have identified a clear patient preference for white coats.[6,7,10,12,14,23,25-41] However, some studies reveal no significant preferences,[42-45] and others indicate that the white coat may even cause higher levels of tension in patients.[44] Some studies have shown that physician attire carries little importance with patients,[46-50] whereas others have shown it has a substantial impact on the patient experience,[30,51] congruent with our results. Literature differs on whether preferences for the white coat change after patients are educated about potential risk of microbial transmission, with some studies showing decreased preference[14,52] and another showing no change.[35] Studies examining attire in countries with bare-below-the-elbow policies have indicated near universal disdain for this infection prevention measure.[27,35] Some studies have shown preference for different forms of attire such as scrubs (e.g., specific circumstances like gastroenterology suites[18,53] and emergencies[5]) and informal attire,[54] and some have revealed no specific patient preferences.[52,55,56] Five studies noted that patient perceptions of compassion, professionalism, and credibility were not associated with a physician's dress.[25,32,57-59] Finally, some studies have demonstrated that attire is more important to patients who are older,[34,51,60] a finding we noted in Japan and the United States.

Studies conducted around the globe have repeatedly demonstrated that context is crucial when considering nonverbal cues like physician dress. Patient viewpoints are associated with a variety of factors such as type of care delivered, type of physician, and even time of day. In one example, Switzerland has a defined healthcare uniform of white scrubs and white coat.[20] This relatively unique phenomenon likely caused patients to expect this form of attire and thus

For peer review only - http://bmjopen.bmj.com/site/about/guidelines.xhtml

strongly prefer it to other forms. In another example from the United States, parents of children being evaluated in the pediatric emergency department were more likely to prefer physicians wearing scrubs but only if their children were experiencing a surgical emergency.[46] Likewise, in that same study, parents who visited the emergency department during the day shift preferred formal attire, whereas those who visited during the night preferred less formal attire.[46] Finally, preferences have also previously been shown to deviate from cultural norms or established national dress.[11,13,30,38] For instance, Batais and colleagues found that patients in family medicine clinics in Saudi Arabia were more likely to adhere to medical recommendations and return for subsequent care if the physician was dressed in Western garb;[60] yet this same population was significantly more willing to discuss personal issues such as psychological problems with a physician wearing Saudi national dress.[60] This finding of preferences that varied based on topic of conversation was noted in other studies as well.[9,10]

A number of strengths distinguish our study from others that have previously investigated patient preferences for physician attire. To our knowledge, this study of over 9,000 participants is the largest study examining opinions on physician dress to date. We employed a standardized survey instrument which allowed direct comparisons across diverse geography and contexts. Randomization of photograph sequence and instrument delivery reduced the risk of ordering, priming, and anchoring bias. We also used photographs containing physician models with identical postures, facial expressions, lighting, and background, all of which limited the confounding associated with previous studies utilizing models of different backgrounds and appearances.[16-18,51,61] Finally, our findings have important policy implications for physician dress code in different care settings and areas of the world.

For peer review only - http://bmjopen.bmj.com/site/about/guidelines.xhtml

Our study also has limitations. Our physician models were young, slender, and either Caucasian or Asian, and as such were not representative of the various sociodemographic characteristics of physicians. Likewise, purposeful differences among survey instruments, including white scrubs instead of blue scrubs in the Switzerland survey and physician models of Japanese descent in the Japan survey, were introduced to ensure relevance. Our study over-represented the United States more so than Japan and the European countries, which could have contributed to skewed results and greater power in any comparison with the United States. For instance, this was particularly evident when examining attire for hospital physicians, in which the highest preference for formal attire with white coat was driven by United States respondents. We did not obtain results from other regions including Africa, Australia, the Middle East, and South America, which could have contributed noteworthy input. Countries yielded different arrays of respondent sociodemographic characteristics such as age and education, which led to disproportionate representation among some groups. The survey instrument used Likert scales with pre-defined categories which may not accurately reflect nuanced patient opinions, and the clinical relevance of small but significant differences in these scales is unknown. The instrument did not capture or explore other elements of etiquette-based patient-physician interaction[62] such as introductions and smiles,[17,18,26,36,45] which are known to be paramount for ensuring effective healthcare relationships. Our study did not assess the relative impact of physician attire compared with the influence from these other relational elements. Finally, the data from several of the individual country-specific studies have been previously published. However, this study is the first instance in which all data are compiled to allow for cross-national comparisons.

In conclusion, the effects of physician attire on the patient experience are complex and multilayered. Our findings suggest that one-size-fits-all physician attire policies which extend to

For peer review only - http://bmjopen.bmj.com/site/about/guidelines.xhtml

all healthcare specialties and contexts are unlikely to reflect the desires and expectations of patients. Instead, our nuanced results that harness direct patient preferences may be used to inform local, regional, and national healthcare policymakers and leaders in their efforts to define physician uniforms. Given that preferences vary, a tailored approach should be sought that matches attire with acuity, setting, and context. This approach is most likely to cultivate the patient-physician relationship and in turn enhance patient satisfaction, trust, confidence, and health outcomes.

For peer review only

For peer review only - http://bmjopen.bmj.com/site/about/guidelines.xhtml

AUTHOR CONTRIBUTIONS

Conception and design of the work: SS, CMP, and VC. **Acquisition of the data**: SS, CMP, LK, VC, MZ, HS, and KK. **Analysis and interpretation of the data**: NH, SS, CMP, LK, DR, LD, MZ, HS, KK, AK, YT, CF, GV, SF, and VC. **Drafting of the manuscript**: NH, CMP, LK, DR, and VC. **Critical revision of the manuscript for important intellectual content**: NH, SS, CMP, LK, DR, LD, MZ, HS, KK, AK, YT, CF, GV, SF, and VC. **Accountable for all aspects of the work**: NH, SS, CMP, LK, DR, LD, MZ, HS, KK, AK, YT, CF, GV, SF, and VC. **Approval of the final manuscript**: NH, SS, CMP, LK, DR, LD, MZ, HS, KK, AK, YT, CF, GV, SF, and VC.

COMPETING INTERESTS

None declared.

FUNDING

This study was partially supported by a Swiss National Science Foundation grant (32003B_149474; PI, HS).

DATA SHARING STATEMENT

Additional unpublished data are not publicly available.

PATIENT CONSENT FOR PUBLICATION

Not required.

For peer review only

ETHICS APPROVAL

The country-specific ethical review committees that reviewed and approved or deemed this project exempt from regulation were the University of Michigan Institutional Review Board (United States, HUM00085305); the Cantonal Ethics Review Board of Zurich, based on the Swiss law on research on humans (Switzerland, No. 60-2015); the ethics committee for Tokyo Joto Hospital (Japan, No. 2015-0001); and the ethics committee for Careggi University Hospital, according to the Declaration of Helsinki (Italy, CE 7123).

AUTHOR NOTE

The views expressed in this article are those of the authors and do not necessarily reflect the position or policy of the Department of Veterans Affairs or the US government.

For peer review only

For peer review only - http://bmjopen.bmj.com/site/about/guidelines.xhtml

REFERENCES

1. Barbosa CD, Balp MM, Kulich K, et al. A literature review to explore the link between treatment satisfaction and adherence, compliance, and persistence. *Patient Prefer Adherence* 2012;6:39–48.
2. O'Malley AS, Forrest CB, Mandelblatt J. Adherence of low-income women to cancer screening recommendations. *J Gen Intern Med* 2002;17:144–54.
3. Boulding W, Glickman SW, Manary MP, et al. Relationship between patient satisfaction with inpatient care and hospital readmission within 30 days. *Am J Manag Care* 2011;17:41–8.
4. Doyle C, Lennox L, Bell D. A systematic review of evidence on the links between patient experience and clinical safety and effectiveness. *BMJ Open* 2013 Jan 3;3(1):e001570. doi: 10.1136/bmjopen-2012-001570. PMID: 23293244; PMCID: PMC3549241.
5. Van De Car W, Starostanko A, Wendling A. Rural Patient Preference for Physician Attire. *PRiMER* 2017 Feb 8;1:3. doi: 10.22454/PRiMER.2017.1.3. PMID: 32944689; PMCID: PMC7490192.
6. Gooden BR, Smith MJ, Tattersall SJ, et al. Hospitalised patients' views on doctors and white coats. *Med J Aust* 2001 Aug 20;175(4):219-22. doi: 10.5694/j.1326-5377.2001.tb143103.x. PMID: 11587285.
7. Rehman SU, Nietert PJ, Cope DW, et al. What to wear today? Effect of doctor's attire on the trust and confidence of patients. *Am J Med* 2005 Nov;118(11):1279-86. doi: 10.1016/j.amjmed.2005.04.026. PMID: 16271913.

For peer review only

For peer review only - http://bmjopen.bmj.com/site/about/guidelines.xhtml

8. Trowbridge RE, Pearson R. Impact of military physician rank and appearance on patient perceptions of clinical competency in a primary care setting. *Mil Med* 2013 Sep;178(9):994-1001. doi: 10.7205/MILMED-D-13-00197. PMID: 24005549.

9. Niederhauser A, Turner MD, Chauhan SP, et al. Physician attire in the military setting: does it make a difference to our patients? *Mil Med* 2009 Aug;174(8):817-20. doi: 10.7205/milmed-d-00-8409. PMID: 19743736.

10. Jennings JD, Ciaravino SG, Ramsey FV, et al. Physicians' Attire Influences Patients' Perceptions in the Urban Outpatient Orthopaedic Surgery Setting. *Clin Orthop Relat Res* 2016 Sep;474(9):1908-18. doi: 10.1007/s11999-016-4855-7. Epub 2016 Apr 26. PMID: 27116208; PMCID: PMC4965372.

11. Chung H, Lee H, Chang DS, et al. Doctor's attire influences perceived empathy in the patient-doctor relationship. *Patient Educ Couns* 2012 Dec;89(3):387-91. doi: 10.1016/j.pec.2012.02.017. Epub 2012 Mar 23. PMID: 22445730.

12. Mun HW, Kim JH, Ahn JH, et al. Patient's Preference on Neurosurgeon's Attire and Appearance: A Single Center Study in Korea Cross-Sectional Study. *Biomed Res Int* 2019 Apr 9;2019:3893049. doi: 10.1155/2019/3893049. PMID: 31093498; PMCID: PMC6481109.

13. Alzahrani HM, Mahfouz AA, Farag S, et al. Patients' perceptions and preferences for physicians' attire in hospitals in south western Saudi Arabia. *J Family Med Prim Care* 2020 Jun 30;9(6):3119-3123. doi: 10.4103/jfmpc.jfmpc_166_20. PMID: 32984183; PMCID: PMC7491852.

For peer review only

For peer review only - http://bmjopen.bmj.com/site/about/guidelines.xhtml

14. Zahrina AZ, Haymond P, Rosanna P, et al. Does the attire of a primary care physician affect patients' perceptions and their levels of trust in the doctor? *Malays Fam Physician* 2018 Dec 31;13(3):3-11. PMID: 30800227; PMCID: PMC6382088.

15. Verghese BG, Kalvehalli Kashinath S, Jadhav N, et al. Physician attire: physicians perspectives on attire in a community hospital setting among non-surgical specialties. *J Community Hosp Intern Med Perspect* 2020 Feb 10;10(1):1-5. doi: 10.1080/20009666.2020.1718478. PMID: 32128050; PMCID: PMC7034488.

16. Au S, Khandwala F, Stelfox HT. Physician attire in the intensive care unit and patient family perceptions of physician professional characteristics. *JAMA Intern Med* 2013 Mar 25;173(6):465-7. doi: 10.1001/jamainternmed.2013.2732. PMID: 23420343.

17. Lill MM, Wilkinson TJ. Judging a book by its cover: descriptive survey of patients' preferences for doctors' appearance and mode of address. *BMJ* 2005 Dec 24;331(7531):1524-7. doi: 10.1136/bmj.331.7531.1524. PMID: 16373739; PMCID: PMC1322253.

18. Sotgiu G, Nieddu P, Mameli L, et al. Evidence for preferences of Italian patients for physician attire. *Patient Prefer Adherence* 2012;6:361-7. doi: 10.2147/PPA.S29587. Epub 2012 Apr 27. PMID: 22573935; PMCID: PMC3346157.

19. Kamata K, Kuriyama A, Chopra V, et al. Patient Preferences for Physician Attire: A Multicenter Study in Japan. *J Hosp Med* 2020 Apr 1;15(4):204-210. doi: 10.12788/jhm.3350. Epub 2020 Feb 11. PMID: 32118558.

20. Zollinger M, Houchens N, Chopra V, et al. Understanding patient preference for physician attire in ambulatory clinics: a cross-sectional observational study. *BMJ Open*

For peer review only - http://bmjopen.bmj.com/site/about/guidelines.xhtml

2019 May 9;9(5):e026009. doi: 10.1136/bmjopen-2018-026009. PMID: 31072853; PMCID: PMC6528053.

21. Petrilli CM, Saint S, Jennings JJ, et al. Understanding patient preference for physician attire: a cross-sectional observational study of 10 academic medical centres in the USA. *BMJ Open* 2018 May 29;8(5):e021239. doi: 10.1136/bmjopen-2017-021239. PMID: 29844101; PMCID: PMC5988098.
22. De Lott LB, Panarelli JF, Samimi D, et al. Patient Preferences for Physician Attire in Ophthalmology Practices. *J Acad Ophthalmol* 2019 Jan;11(1):e36-e42. doi: 10.1055/s-0039-1688913. PMID: 31475243; PMCID: PMC6716523.
23. Petrilli CM, Mack M, Petrilli JJ, et al. Understanding the role of physician attire on patient perceptions: a systematic review of the literature--targeting attire to improve likelihood of rapport (TAILOR) investigators. *BMJ Open* 2015 Jan 19;5(1):e006578. doi: 10.1136/bmjopen-2014-006578. PMID: 25600254; PMCID: PMC4312788.
24. Karnieli-Miller O, Frankel RM, Inui TS. Cloak of compassion, or evidence of elitism? an empirical analysis of white coat ceremonies. *Med Educ* 2013 Jan;47(1):97-108. doi: 10.1111/j.1365-2923.2012.04324.x. PMID: 23278829.
25. Al-Ghobain MO, Al-Drees TM, Alarifi MS, et al. Patients' preferences for physicians' attire in Saudi Arabia. *Saudi Med J* 2012 Jul;33(7):763-7. PMID: 22821311.
26. Major K, Hayase Y, Balderrama D, et al. Attitudes regarding surgeons' attire. *Am J Surg* 2005 Jul;190(1):103-6. doi: 10.1016/j.amjsurg.2005.04.003. PMID: 15972180.
27. Meshkat B, Bass GA, Matcovici M, et al. Patients attitude towards surgeons attire in Our Lady of Lourdes Hospital Drogheda. *Int J Health Policy Manag* 2015 Feb 15;4(4):217-20. doi: 10.15171/ijhpm.2015.31. PMID: 25844382; PMCID: PMC4380563.

For peer review only - http://bmjopen.bmj.com/site/about/guidelines.xhtml

28. Lands VW, Malige A, Nwachuku CO, et al. The Effect of an Orthopedic Hand Surgeon's Attire on Patient Confidence and Trust. *Hand (N Y)* 2019 Sep;14(5):675-683. doi: 10.1177/1558944717750918. Epub 2018 Jan 18. PMID: 29343100; PMCID: PMC6759972.

29. Yamada Y, Takahashi O, Ohde S, et al. Patients' preferences for doctors' attire in Japan. *Intern Med* 2010;49(15):1521-6. doi: 10.2169/internalmedicine.49.3572. Epub 2010 Aug 2. PMID: 20686283.

30. Aldrees T, Alsuhaibani R, Alqaryan S, et al. Physicians' attire. Parents preferences in a tertiary hospital. *Saudi Med J* 2017 Apr;38(4):435-439. doi: 10.15537/smj.2017.4.15853. PMID: 28397953; PMCID: PMC5447199.

31. Al Amry KM, Al Farrah M, Ur Rahman S, et al. Patient perceptions and preferences of physicians' attire in Saudi primary healthcare setting. *J Community Hosp Intern Med Perspect* 2018 Dec 11;8(6):326-330. doi: 10.1080/20009666.2018.1551026. PMID: 30559939; PMCID: PMC6292367.

32. Carugno J, Timmons D, Grady M, et al. Impact of physician attire on patients' impression of their gynecologist: Results from a large single-center survey analysis. *Eur J Obstet Gynecol Reprod Biol* 2020 Nov;254:266-270. doi: 10.1016/j.ejogrb.2020.09.040. Epub 2020 Sep 23. PMID: 33035822.

33. Maruani A, Léger J, Giraudeau B, et al. Effect of physician dress style on patient confidence. *J Eur Acad Dermatol Venereol* 2013 Mar;27(3):e333-7. doi: 10.1111/j.1468-3083.2012.04665.x. Epub 2012 Aug 9. PMID: 22882283.

For peer review only - http://bmjopen.bmj.com/site/about/guidelines.xhtml

34. Kurihara H, Maeno T, Maeno T. Importance of physicians' attire: factors influencing the impression it makes on patients, a cross-sectional study. *Asia Pac Fam Med* 2014 Jan 8;13(1):2. doi: 10.1186/1447-056X-13-2. PMID: 24397871; PMCID: PMC3890493.

35. Landry M, Dornelles AC, Hayek G, et al. Patient Preferences for Doctor Attire: The White Coat's Place in the Medical Profession. *Ochsner J* 2013 Fall;13(3):334-42. PMID: 24052762; PMCID: PMC3776508.

36. Matsui D, Cho M, Rieder MJ. Physicians' attire as perceived by young children and their parents: the myth of the white coat syndrome. *Pediatr Emerg Care* 1998 Jun;14(3):198-201. doi: 10.1097/00006565-199806000-00006. PMID: 9655662.

37. Iram S, Prakash WD, Ali MJ, et al. Preferences of ophthalmic plastics patients and their caregivers toward the doctors' attire and initial communications: A tertiary eye care study. *Indian J Ophthalmol* 2016 Jun;64(6):448-51. doi: 10.4103/0301-4738.187674. PMID: 27488153; PMCID: PMC4991175.

38. Chang DS, Lee H, Lee H, et al. What to wear when practicing oriental medicine: patients' preferences for doctors' attire. *J Altern Complement Med* 2011 Aug;17(8):763-7. doi: 10.1089/acm.2010.0612. Epub 2011 Jul 1. PMID: 21721926.

39. Gallagher J, Waldron Lynch F, Stack J, et al. Dress and address: patient preferences regarding doctor's style of dress and patient interaction. *Ir Med J* 2008 Jul-Aug;101(7):211-3. PMID: 18807811.

40. Gherardi G, Cameron J, West A, et al. Are we dressed to impress? A descriptive survey assessing patients' preference of doctors' attire in the hospital setting. *Clin Med (Lond)* 2009 Dec;9(6):519-24. doi: 10.7861/clinmedicine.9-6-519. PMID: 20095290; PMCID: PMC4952286.

For peer review only - http://bmjopen.bmj.com/site/about/guidelines.xhtml

41. McNaughton-Filion L, Chen JS, Norton PG. The physician's appearance. *Fam Med* 1991 Mar-Apr;23(3):208-11. PMID: 2016013.

42. La Rosa M, Spencer N, Abdelwahab M, et al. The Effect of Wearing White Coats on Patients' Appreciation of Physician Communication during Postpartum Rounds: A Randomized Controlled Trial. *Am J Perinatol* 2019 Jan;36(1):62-66. doi: 10.1055/s-0038-1660470. Epub 2018 Jun 8. PMID: 29883984.

43. Cha A, Hecht BR, Nelson K, et al. Resident physician attire: does it make a difference to our patients? *Am J Obstet Gynecol* 2004 May;190(5):1484-8. doi: 10.1016/j.ajog.2004.02.022. PMID: 15167876.

44. Ikusaka M, Kamegai M, Sunaga T, et al. Patients' attitude toward consultations by a physician without a white coat in Japan. *Intern Med* 1999 Jul;38(7):533-6. doi: 10.2169/internalmedicine.38.533. PMID: 10435357.

45. Varnado-Sullivan P, Larzelere M, Solek K, et al. The Impact of Physician Demographic Characteristics on Perceptions of Their Attire. *Fam Med* 2019 Oct 4;51(9):737-741. doi: 10.22454/FamMed.2019.650493. Epub 2019 Aug 26. PMID: 31465109.

46. Gonzalez Del Rey JA, Paul RI. Preferences of parents for pediatric emergency physicians' attire. *Pediatr Emerg Care* 1995 Dec;11(6):361-4. doi: 10.1097/00006565-199512000-00007. PMID: 8751171.

47. Li SF, Haber M. Patient attitudes toward emergency physician attire. *J Emerg Med* 2005 Jul;29(1):1-3. doi: 10.1016/j.jemermed.2004.12.014. PMID: 15960999.

48. Menahem S, Shvartzman P. Is our appearance important to our patients? *Fam Pract* 1998 Oct;15(5):391-7. doi: 10.1093/fampra/15.5.391. PMID: 9848422.

For peer review only

For peer review only - http://bmjopen.bmj.com/site/about/guidelines.xhtml

49. Friis R, Tilles J. Patients' preferences for resident physician dress style. *Fam Pract Res J* 1988 Fall-Winter;8(1):24-31. PMID: 3239442.

50. McLean C, Patel P, Sullivan C, et al. Patients' perception of military doctors in fracture clinics--does the wearing of uniform make a difference? *J R Nav Med Serv* 2005;91(1):45-7. PMID: 15986841.

51. McKinstry B, Wang JX. Putting on the style: what patients think of the way their doctor dresses. *Br J Gen Pract* 1991 Jul;41(348):270, 275-8. PMID: 1747264; PMCID: PMC1371685.

52. Hueston WJ, Carek SM. Patients' preference for physician attire: a survey of patients in family medicine training practices. *Fam Med* 2011 Oct;43(9):643-7. PMID: 22002776.

53. Clark M, Shuja A, Thomas A, et al. Patients' perceptions of gastroenterologists' attire in the clinic and endoscopy suite. *Ann Gastroentero*l 2018 Mar-Apr;31(2):237-240. doi: 10.20524/aog.2017.0223. Epub 2017 Dec 22. PMID: 29507472; PMCID: PMC5825955.

54. Reddy R. Slippers and a white coat? (Hawai'i physician attire study). *Hawaii Med J* 2009 Dec;68(11):284-5. PMID: 20034257.

55. Fischer RL, Hansen CE, Hunter RL, et al. Does physician attire influence patient satisfaction in an outpatient obstetrics and gynecology setting? *Am J Obstet Gynecol* 2007 Feb;196(2):186.e1-5. doi: 10.1016/j.ajog.2006.09.043. PMID: 17306675.

56. Longmuir S, Gilbertson A, Pfeifer W, et al. Pediatric ophthalmology attire: should we wear a white coat? *Insight* 2010 Oct-Dec;35(4):11-3. PMID: 21189797.

57. Azhar A, Tanco K, Haider A, et al. Challenging the status quo of physician attire in the palliative care setting. *Oncologist* 2020 Jul;25(7):627-637. doi:

For peer review only

For peer review only - http://bmjopen.bmj.com/site/about/guidelines.xhtml

10.1634/theoncologist.2019-0568. Epub 2020 Feb 19. PMID: 32073181; PMCID: PMC7356715.

58. Traeger AC, Skinner IW, Hübscher M, et al. What you wear does not affect the credibility of your treatment: A blinded randomized controlled study. *Patient Educ Couns* 2017 Jan;100(1):104-111. doi: 10.1016/j.pec.2016.08.009. Epub 2016 Aug 8. PMID: 27522250.

59. Boon D, Wardrope J. What should doctors wear in the accident and emergency department? Patients' perception. *J Accid Emerg Med* 1994 Sep;11(3):175-7. doi: 10.1136/emj.11.3.175. PMID: 7804584; PMCID: PMC1342426.

60. Batais MA. Patients' attitudes toward the attire of male physicians: a single-center study in Saudi Arabia. *Ann Saudi Med* 2014 Sep-Oct;34(5):383-9. doi: 10.5144/0256-4947.2014.383. PMID: 25827694; PMCID: PMC6074558.

61. Kocks JW, Lisman-van Leeuwen Y, Berkelmans PG. De kleren maken de dokter--meer vertrouwen in netter geklede huisarts [Clothing make the doctor--patients have more confidence in a smartly dressed GP]. *Ned Tijdschr Geneeskd* 2010;154(51-52):A2898. Dutch. PMID: 21211081.

62. Kahn MW. Etiquette-based medicine. *N Engl J Med* 2008 May 8;358(19):1988-9. doi: 10.1056/NEJMp0801863. PMID: 18463374.

For peer review only

***Table 1*: Characteristics of participating study sites**

Country	Dates of Data Collection	Types of Outpatient Clinics	Clinical Setting(s)	Hospitals, Practices	Geographic Regions Sampled	Surveys Completed
Italy	10/26/2015-10/21/2016	Infectious Disease, Ophthalmology, Geriatric Intensive Care Unit	Outpatient and Inpatient	1	1[*]	958
Japan	12/01/2015-10/30/2017	General Medicine, Medicine Specialties, Orthopedic Surgery	Outpatient and Inpatient	4	3[†]	2020
Switzerland	06/15/2015-10/31/2016	Dermatology, Infectious Disease, Neurology	Outpatient	1	1[‡]	834
United States (US)[§]	06/01/2015-10/31/2016	General Medicine, Medicine Specialties	Outpatient and Inpatient	10	4[**]	4062
		Ophthalmology	Outpatient	4	3[††]	1297

[*] One site in the Tuscany region
[†] Two sites in the Kantō region; one site in the Kansai region; one site in the Chūgoku region
[‡] One site in the Canton of Zurich
[§] Geographic regions of the US include Northeast, Midwest, South, and West
[**] Three sites in the Midwest, three sites in the South, two sites in the Northeast, two sites in the West
[††] Two sites in the Midwest, one site in the Northeast, one site in the West

For peer review only

For peer review only - http://bmjopen.bmj.com/site/about/guidelines.xhtml

***Table 2*: Sociodemographic information**

	Italy (n=958)	Japan (n=2020)	Switzerland (n=834)	United States (n=5359)	Total (n=9171)
**Age**	n=928	n=2010	n=812	n=5279	n=9029
18-25	61 (6.6%)	67 (3.3%)	50 (6.2%)	241 (4.6%)	419 (4.6%)
26-34	89 (9.6%)	162 (8.1%)	93 (11.5%)	464 (8.8%)	808 (9.0%)
35-54	310 (33.4%)	461 (22.9%)	341 (42.0%)	1299 (24.6%)	2411 (26.7%)
55-64	210 (22.6%)	345 (17.2%)	192 (23.6%)	1393 (26.4%)	2140 (23.7%)
≥65	258 (27.8%)	975 (48.5%)	136 (16.7%)	1882 (35.6%)	3251 (36.0%)
**Gender**	n=905	n=2011	n=806	n=5194	n=8916
Female	471 (52.0%)	1040 (51.7%)	304 (37.7%)	2184 (42.0%)	3999 (44.9%)
Male	434 (48.0%)	971 (48.3%)	502 (62.3%)	3010 (58.0%)	4917 (55.1%)
**Education**	n=919	n=2010	n=808	n=5247	n=8984
Less than high school	237 (25.8%)	243 (12.1%)	368 (45.5%)	146 (2.8%)	994 (11.1%)
High school diploma	416 (45.3%)	1236 (61.5%)	82 (10.2%)	2691 (51.3%)	4425 (49.3%)
College degree	77 (8.4%)	487 (24.2%)	340 (42.1%)	1490 (28.4%)	2394 (26.6%)
Graduate degree	189 (20.5%)	44 (2.2%)	18 (2.2%)	920 (17.5%)	1171 (13.0%)
**Number of unique physicians seen in the past year**	n=928	n=2009	n=810	n=5265	n=9012
0	76 (8.2%)	38 (1.9%)	13 (1.6%)	51 (1.0%)	178 (2.0%)
1	126 (13.6%)	140 (7.0%)	83 (10.2%)	377 (7.2%)	726 (8.1%)
2	199 (21.4%)	373 (18.5%)	165 (20.4%)	769 (14.6%)	1506 (16.7%)
3	188 (20.3%)	512 (25.5%)	203 (25.1%)	940 (17.9%)	1843 (20.4%)
4	112 (12.1%)	359 (17.9%)	126 (15.6%)	824 (15.6%)	1421 (15.8%)
5	84 (9.0%)	225 (11.2%)	57 (7.0%)	571 (10.8%)	937 (10.4%)
≥6	143 (15.4%)	362 (18.0%)	163 (20.1%)	1733 (32.9%)	2401 (26.6%)

For peer review only - http://bmjopen.bmj.com/site/about/guidelines.xhtml

***Table 3*: Preferred physician attire by physician type and care setting**

Physician Type	Attire	Italy	Japan	Switzerland	United States	Total
Primary Care Physician	Casual	103 (11.0%)	33 (1.6%)	199 (24.4%)	158 (3.0%)	493 (5.5%)
	Casual with white coat	165 (17.6%)	682 (34.1%)	183 (22.4%)	518 (9.9%)	1548 (17.2%)
	Scrubs	61 (6.5%)	188 (9.4%)	90 (11.0%)	238 (4.6%)	577 (6.4%)
	Scrubs with white coat	126 (13.5%)	357 (17.9%)	78 (9.6%)	742 (14.2%)	1303 (14.5%)
	Formal	128 (13.7%)	49 (2.5%)	73 (8.9%)	787 (15.0%)	1037 (11.6%)
	Formal with white coat	296 (31.6%)	669 (33.4%)	188 (23.0%)	2451 (46.8%)	3604 (40.1%)
	Business suit	57 (6.1%)	22 (1.1%)	6 (0.7%)	340 (6.5%)	425 (4.7%)
Emergency Department Physician	Casual	36 (3.9%)	42 (2.1%)	31 (3.8%)	63 (1.2%)	172 (1.9%)
	Casual with white coat	89 (9.6%)	206 (10.3%)	65 (8.0%)	298 (5.7%)	658 (7.3%)
	Scrubs	343 (37.2%)	1131 (56.5%)	382 (46.9%)	2108 (40.2%)	3964 (44.2%)
	Scrubs with white coat	324 (35.1%)	354 (17.7%)	271 (33.3%)	1784 (34.1%)	2733 (30.4%)
	Formal	16 (1.7%)	61 (3.0%)	8 (1.0%)	134 (2.6%)	219 (2.4%)
	Formal with white coat	105 (11.4%)	204 (10.2%)	52 (6.4%)	793 (15.1%)	1154 (12.9%)
	Business suit	10 (1.1%)	5 (0.2%)	5 (0.6%)	60 (1.1%)	80 (0.9%)
Hospital Physician	Casual	25 (2.7%)	19 (1.0%)	33 (4.1%)	68 (1.3%)	145 (1.6%)
	Casual with white coat	98 (10.6%)	680 (34.0%)	138 (17.0%)	435 (8.3%)	1351 (15.1%)
	Scrubs	176 (19.1%)	162 (8.1%)	203 (25.0%)	594 (11.4%)	1135 (12.7%)
	Scrubs with white coat	404 (43.8%)	444 (22.2%)	285 (35.0%)	1600 (30.7%)	2733 (30.5%)
	Formal	17 (1.8%)	26 (1.3%)	20 (2.4%)	346 (6.6%)	409 (4.6%)
	Formal with white coat	189 (20.5%)	660 (33.0%)	129 (15.9%)	1964 (37.6%)	2942 (32.8%)
	Business suit	14 (1.5%)	9 (0.4%)	5 (0.6%)	212 (4.1%)	240 (2.7%)
Surgeon	Casual	32 (3.5%)	13 (0.6%)	17 (2.1%)	37 (0.7%)	99 (1.1%)
	Casual with white coat	85 (9.2%)	238 (11.9%)	44 (5.4%)	179 (3.4%)	546 (6.1%)
	Scrubs	289 (31.2%)	942 (47.1%)	345 (42.6%)	2224 (42.5%)	3800 (42.4%)
	Scrubs with white coat	302 (32.6%)	501 (25.0%)	272 (33.6%)	1202 (23.0%)	2277 (25.4%)
	Formal	37 (4.0%)	35 (1.8%)	17 (2.1%)	192 (3.7%)	281 (3.1%)
	Formal with white coat	155 (16.8%)	266 (13.3%)	108 (13.3%)	1102 (21.1%)	1631 (18.2%)
	Business suit	25 (2.7%)	6 (0.3%)	7 (0.9%)	291 (5.6%)	329 (3.7%)
Overall	Casual	20 (2.2%)	17 (0.9%)	46 (5.8%)	70 (1.4%)	153 (1.7%)

For peer review only - http://bmjopen.bmj.com/site/about/guidelines.xhtml

	Casual with white coat	94 (10.2%)	606 (30.3%)	136 (17.0%)	367 (7.1%)	1203 (13.5%)
	Scrubs	146 (15.8%)	203 (10.1%)	205 (25.6%)	390 (7.5%)	944 (10.6%)
	Scrubs with white coat	385 (41.7%)	436 (21.8%)	252 (31.5%)	1289 (24.8%)	2362 (26.5%)
	Formal	25 (2.7%)	26 (1.3%)	22 (2.7%)	448 (8.6%)	521 (5.9%)
	Formal with white coat	235 (25.5%)	707 (35.3%)	131 (16.4%)	2370 (45.7%)	3443 (38.6%)
	Business suit	18 (1.9%)	7 (0.3%)	8 (1.0%)	255 (4.9%)	288 (3.2%)

For peer review only

For peer review only - http://bmjopen.bmj.com/site/about/guidelines.xhtml

Table 4: **Respondent opinions regarding importance, influence, and appropriateness of physician attire and white coats**

		**Italy**	**Japan**	**Switzerland**	**United States**	**Total**
How my doctor dresses is important to me.	Strongly disagree	60 (6.4%)	67 (3.3%)	110 (13.4%)	222 (4.2%)	459 (5.1%)
	Disagree	87 (9.4%)	280 (13.9%)	151 (18.4%)	531 (10.0%)	1049 (11.6%)
	Neither agree nor disagree	220 (23.7%)	430 (21.4%)	260 (31.8%)	1603 (30.2%)	2513 (27.7%)
	Agree	410 (44.1%)	1031 (51.3%)	185 (22.6%)	2303 (43.5%)	3929 (43.4%)
	Strongly agree	153 (16.4%)	202 (10.1%)	113 (13.8%)	641 (12.1%)	1109 (12.2%)
	Mean*	3.55	3.51	3.05	3.49	
How my doctor dresses influences how happy I am with the care I receive.	Strongly disagree	132 (14.3%)	124 (6.2%)	223 (27.3%)	334 (6.3%)	813 (9.0%)
	Disagree	209 (22.6%)	396 (19.7%)	235 (28.8%)	851 (16.1%)	1691 (18.7%)
	Neither agree nor disagree	250 (27.0%)	536 (26.7%)	171 (20.9%)	2088 (39.5%)	3045 (33.7%)
	Agree	263 (28.5%)	812 (40.5%)	124 (15.2%)	1633 (30.9%)	2832 (31.3%)
	Strongly agree	70 (7.6%)	138 (6.9%)	64 (7.8%)	384 (7.2%)	656 (7.3%)
	Mean*	2.92	3.22	2.47	3.17	
It is appropriate for a doctor to dress casually when seeing patients over the weekend.	Strongly disagree	81 (8.7%)	209 (10.4%)	104 (12.8%)	182 (3.5%)	576 (6.4%)
	Disagree	213 (22.9%)	837 (41.7%)	139 (17.2%)	955 (18.1%)	2144 (23.7%)
	Neither agree nor disagree	218 (23.4%)	613 (30.5%)	147 (18.2%)	1761 (33.3%)	2739 (30.3%)
	Agree	326 (35.1%)	300 (15.0%)	189 (23.4%)	2047 (38.7%)	2862 (31.7%)
	Strongly agree	92 (9.9%)	48 (2.4%)	230 (28.4%)	340 (6.4%)	340 (7.9%)
	Mean*	3.15	2.57	3.37	3.27	
Doctors should wear a white coat when seeing patients in their office.	Strongly disagree	20 (2.2%)	48 (2.4%)	108 (13.2%)	84 (1.6%)	260 (2.9%)
	Disagree	47 (5.1%)	226 (11.2%)	132 (16.1%)	552 (10.4%)	957 (10.6%)
	Neither agree nor disagree	139 (14.9%)	437 (21.7%)	170 (20.8%)	1698 (32.1%)	2444 (27.0%)
	Agree	504 (54.1%)	1085 (54.0%)	251 (30.7%)	2361 (44.7%)	4201 (46.4%)
	Strongly agree	221 (23.7%)	214 (10.7%)	157 (19.2%)	593 (11.2%)	1185 (13.1%)
	Mean*	3.92	3.59	3.27	3.53	
Doctors should wear a white coat when seeing patients in the	Strongly disagree	15 (1.6%)	102 (5.1%)	47 (5.8%)	111 (2.1%)	275 (3.0%)
	Disagree	36 (3.8%)	541 (27.0%)	56 (6.9%)	828 (15.6%)	1461 (16.2%)
	Neither agree nor disagree	115 (12.3%)	623 (31.1%)	75 (9.2%)	1952 (36.9%)	2765 (30.6%)

For peer review only - http://bmjopen.bmj.com/site/about/guidelines.xhtml

emergency department.	Agree	480 (51.2%)	628 (31.3%)	294 (36.0%)	1973 (37.3%)	3375 (37.3%)
	Strongly agree	291 (31.1%)	110 (5.5%)	343 (42.1%)	426 (8.1%)	1170 (12.9%)
	Mean*	4.06	3.05	4.02	3.34	
Doctors should wear a white coat when seeing patients in the hospital	Strongly disagree	13 (1.4%)	45 (2.2%)	50 (6.1%)	65 (1.2%)	173 (1.9%)
	Disagree	19 (2.0%)	236 (11.7%)	45 (5.5%)	401 (7.6%)	701 (7.7%)
	Neither agree nor disagree	83 (8.8%)	441 (22.0%)	128 (15.7%)	1507 (28.5%)	2159 (23.9%)
	Agree	509 (54.3%)	1114 (55.4%)	311 (38.2%)	2756 (52.1%)	4690 (51.8%)
	Strongly agree	314 (33.5%)	174 (8.7%)	281 (34.5%)	560 (10.6%)	1329 (14.7%)
	Mean*	4.16	3.57	3.89	3.63	
Doctors should always wear a white coat when seeing patients in any setting.	Strongly disagree	23 (2.5%)	109 (5.4%)	179 (21.9%)	181 (3.4%)	492 (5.4%)
	Disagree	119 (12.7%)	567 (28.2%)	164 (20.0%)	1140 (21.5%)	1990 (22.0%)
	Neither agree nor disagree	269 (28.7%)	682 (33.9%)	202 (24.7%)	2147 (40.6%)	3300 (36.4%)
	Agree	361 (38.5%)	550 (27.4%)	169 (20.7%)	1497 (28.3%)	2577 (28.5%)
	Strongly agree	165 (17.6%)	103 (5.1%)	104 (12.7%)	326 (6.2%)	698 (7.7%)
	Mean*	3.56	2.99	2.82	3.12	

* Means calculated with scores of 1 assigned to “strongly disagree,” 3 to “neither agree nor disagree,” and 5 to “strongly agree.”

For peer review only

For peer review only - http://bmjopen.bmj.com/site/about/guidelines.xhtml

FIGURE LEGEND

***Figure 1*: Mean composite ratings of physician attire**

For peer review only

For peer review only - http://bmjopen.bmj.com/site/about/guidelines.xhtml

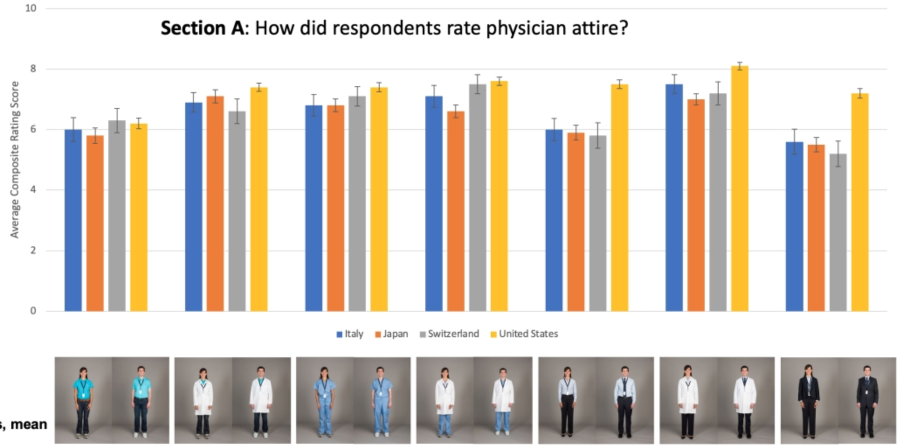

Countries, mean (SD)							
Italy	6.0 (2.3)	6.9 (1.8)	6.8 (2.1)	7.1 (2.0)	6.0 (2.2)	7.5 (1.8)	5.6 (2.4)
Japan	5.8 (2.2)	7.1 (1.8)	6.8 (1.8)	6.6 (1.8)	5.9 (2.1)	7.0 (1.6)	5.5 (2.1)
Switzerland	6.3 (2.2)	6.6 (2.3)	7.1 (1.7)	7.5 (1.7)	5.8 (2.3)	7.2 (1.9)	5.2 (2.2)
United States	6.2 (2.5)	7.4 (2.0)	7.4 (2.0)	7.6 (2.0)	7.5 (2.0)	8.1 (1.8)	7.2 (2.2)

Mean composite ratings of physician attire

443x261mm (144 x 144 DPI)

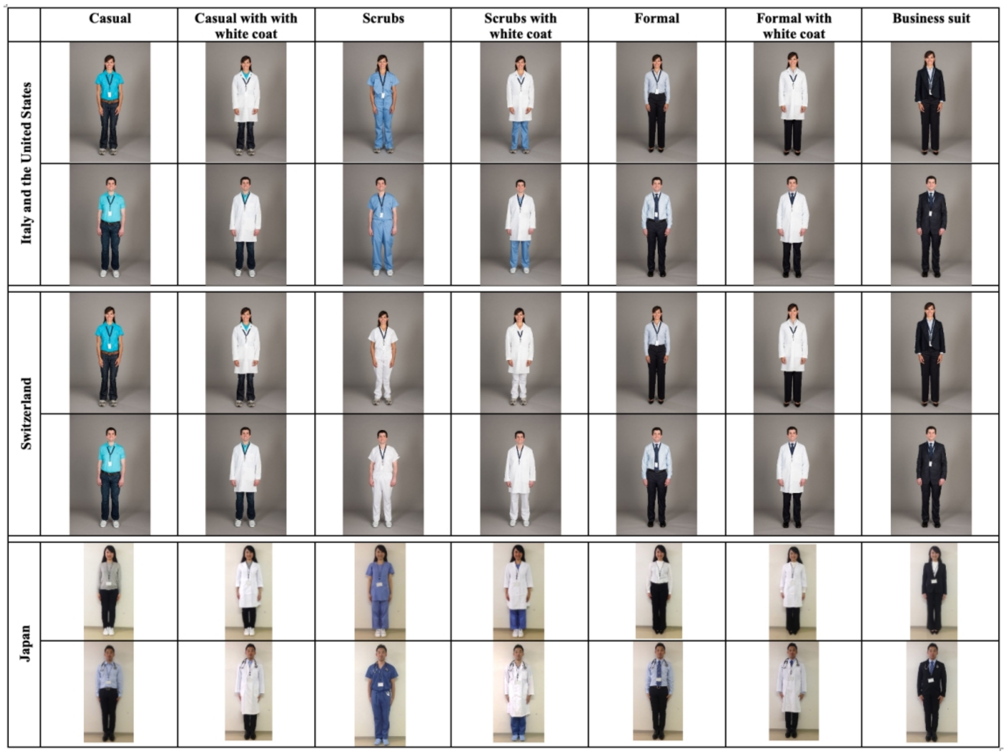

272x203mm (144 x 144 DPI)

Appendix B: Survey Instrument

Section A – Physician Attire - Ratings

Please rate the doctor for each of the following questions by circling the number that corresponds to your answer.

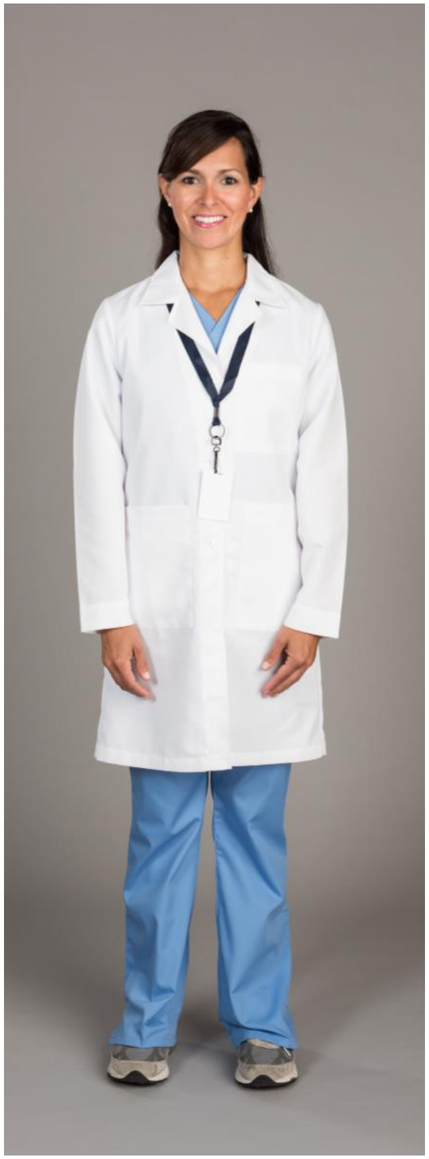

1) How **knowledgeable** does this doctor appear?	1 2 3 4 5 6 7 8 9 10<br>Somewhat … Extremely
2) How **trustworthy** does this doctor appear?	1 2 3 4 5 6 7 8 9 10<br>Somewhat … Extremely
3) How **caring** does this doctor appear?	1 2 3 4 5 6 7 8 9 10<br>Somewhat … Extremely
4) How **approachable** does this doctor appear?	1 2 3 4 5 6 7 8 9 10<br>Somewhat … Extremely
5) How **comfortable** does this doctor make you feel?	1 2 3 4 5 6 7 8 9 10<br>Somewhat … Extremely

For peer review only

Section B – Physician Attire - Preferences

Please provide your ONE best answer to each of the following questions

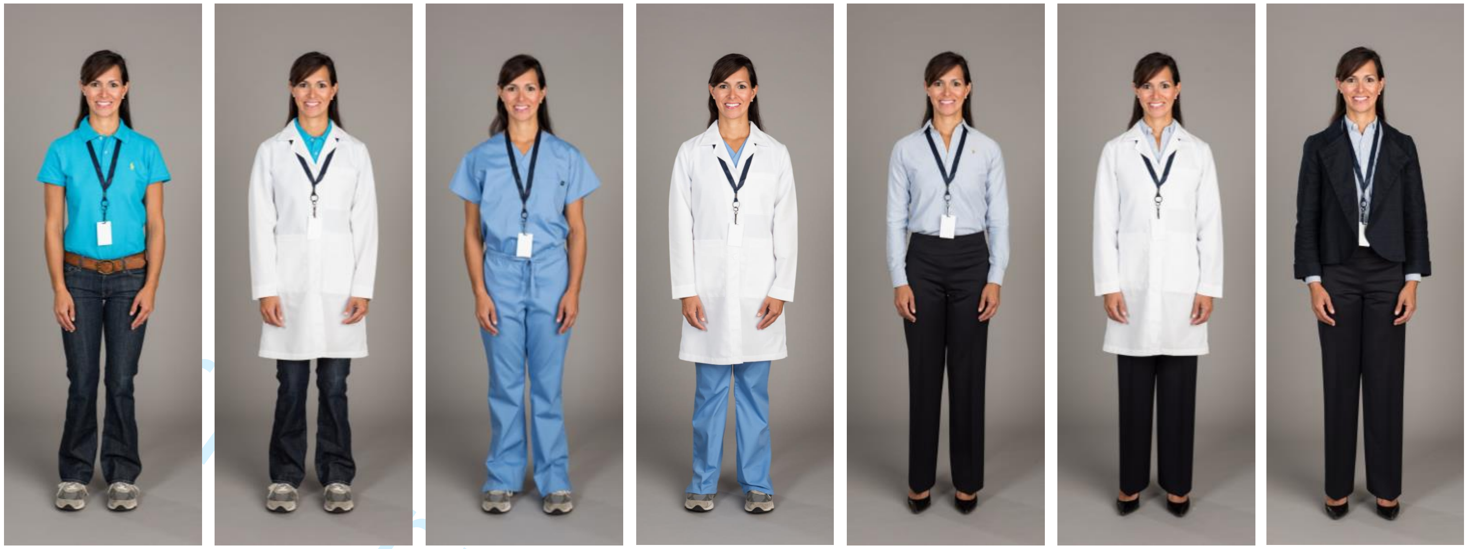

A **B** **C** **D** **E** **F** **G**

6) Which doctor would you prefer for your **primary care doctor**? (Please select only ONE option)

☐ **A** ☐ **B** ☐ **C** ☐ **D** ☐ **E** ☐ **F** ☐ **G**

7) Which doctor would you prefer to see when visiting the **emergency room**? (Please select only ONE option)

☐ **A** ☐ **B** ☐ **C** ☐ **D** ☐ **E** ☐ **F** ☐ **G**

8) Which doctor would you prefer to see when **in the hospital**? (Please select only ONE option)

☐ **A** ☐ **B** ☐ **C** ☐ **D** ☐ **E** ☐ **F** ☐ **G**

9) Which doctor would you prefer for your **surgeon**? (Please select only ONE option)

☐ **A** ☐ **B** ☐ **C** ☐ **D** ☐ **E** ☐ **F** ☐ **G**

10) **Overall**, which clothes do you feel doctors should wear? (Please select only ONE option)

☐ **A** ☐ **B** ☐ **C** ☐ **D** ☐ **E** ☐ **F** ☐ **G**

For peer review only - http://bmjopen.bmj.com/site/about/guidelines.xhtml

Section C – General Physician Attire

Please indicate your level of agreement with the following statements by checking ONE box to the left of your answer.

11) How my doctor dresses is important to me.

☐ Strongly Disagree ☐ Disagree ☐ Neither Agree nor Disagree ☐ Agree ☐ Strongly Agree

12) How my doctor dresses influences how happy I am with the care I receive.

☐ Strongly Disagree ☐ Disagree ☐ Neither Agree nor Disagree ☐ Agree ☐ Strongly Agree

13) It is appropriate for a doctor to dress casually when seeing patients **over the weekend.**

☐ Strongly Disagree ☐ Disagree ☐ Neither Agree nor Disagree ☐ Agree ☐ Strongly Agree

14) Doctors should wear a white coat when seeing patients **in their office or clinic**.

☐ Strongly Disagree ☐ Disagree ☐ Neither Agree nor Disagree ☐ Agree ☐ Strongly Agree

15) Doctors should wear a white coat when seeing patients in the **emergency room**.

☐ Strongly Disagree ☐ Disagree ☐ Neither Agree nor Disagree ☐ Agree ☐ Strongly Agree

16) Doctors should wear a white coat when seeing patients **in the hospital**.

☐ Strongly Disagree ☐ Disagree ☐ Neither Agree nor Disagree ☐ Agree ☐ Strongly Agree

17) Doctors should always wear a white coat when seeing patients **in any setting**.

☐ Strongly Disagree ☐ Disagree ☐ Neither Agree nor Disagree ☐ Agree ☐ Strongly Agree

For peer review only

D

Section D – Demographics

Please remember that all of your answers will be kept confidential.

18) How old are you?

☐ 18-25 ☐ 26-34 ☐ 35-54 ☐ 55-64 ☐ 65 or older

19) What is your gender?

☐ Male ☐ Female

20) What is the highest level of education you have completed?

☐ Less than High School ☐ High School ☐ Some College ☐ College ☐ Graduate Degree

21) What is your race?

☐ American Indian/Alaska Native ☐ Asian ☐ Native Hawaiian or Other Pacific Islander
☐ Black or African American ☐ White ☐ Hispanic
☐ Other (Please specify) ___________________________________

22) How many different doctors have you seen in the past year?

☐ 0 ☐ 1 ☐ 2 ☐ 3 ☐ 4 ☐ 5 ☐ 6 or more

Thank you for taking the time to fill out our survey.

Your input is greatly appreciated.

For peer review only

Appendix C. **Composite ratings of physician attire by domain**

Attire	Domain	Italy			Japan			Switzerland			United States		
		n	Mean	SD	n	Mean	SD	n	Mean	SD	n	Mean	SD
Casual	knowledgeable	137	5.2	2.5	285	5.3	2.4	118	5.6	2.4	752	5.4	2.7
	trustworthy	136	5.5	2.6	286	5.4	2.4	117	6.0	2.4	752	6.0	2.7
	caring	134	6.2	2.4	286	6.2	2.3	119	6.4	2.4	751	6.4	2.6
	approachable	137	6.7	2.3	286	6.5	2.3	119	7.1	2.2	752	6.7	2.6
	comfort	138	6.4	2.8	286	5.8	2.4	117	6.4	2.4	754	6.3	2.8
	mean score	133	6.0	2.3	285	5.8	2.2	115	6.3	2.2	748	6.2	2.5
Casual with white coat	knowledgeable	133	6.3	2.1	288	6.7	2.1	125	6.1	2.4	759	7.2	2.2
	trustworthy	133	6.5	2.1	288	6.8	2.1	124	6.5	2.4	757	7.4	2.2
	caring	133	7.1	2.0	288	7.3	1.9	122	6.6	2.5	759	7.5	2.1
	approachable	133	7.4	1.9	288	7.5	1.9	124	7.1	2.4	764	7.7	2.1
	comfort	133	7.2	2.0	288	7.1	2.1	123	6.5	2.5	759	7.5	2.2
	mean score	133	6.9	1.8	288	7.1	1.8	121	6.6	2.3	747	7.4	2.0
Scrubs	knowledgeable	136	6.2	2.4	283	6.3	2.1	114	6.8	2.0	747	7.0	2.3
	trustworthy	135	6.4	2.3	283	6.5	2.1	116	7.2	2.0	747	7.3	2.2
	caring	134	6.9	2.2	283	7.0	1.9	115	7.0	2.0	746	7.5	2.1
	approachable	136	7.2	2.1	283	7.2	1.8	115	7.4	1.7	749	7.7	2.1
	comfort	136	7.1	2.4	283	6.8	2.0	114	7.1	2.1	749	7.5	2.3
	mean score	134	6.8	2.1	283	6.8	1.8	113	7.1	1.7	742	7.4	2.0
Scrubs with white coat	knowledgeable	126	6.7	2.2	288	6.1	2.0	122	7.1	2.0	761	7.5	2.1
	trustworthy	128	6.9	2.3	290	6.2	2.0	122	7.5	2.1	759	7.6	2.1
	caring	126	7.1	2.3	290	6.8	2.0	121	7.4	2.0	757	7.6	2.1
	approachable	127	7.4	2.0	290	7.2	2.0	120	7.8	1.9	761	7.8	2.1
	comfort	128	7.3	2.2	290	6.6	2.1	121	7.5	1.9	760	7.7	2.2
	mean score	125	7.1	2.0	288	6.6	1.8	120	7.5	1.7	753	7.6	2.0
Formal	knowledgeable	137	5.6	2.4	286	5.5	2.3	121	5.6	2.4	759	7.4	2.1
	trustworthy	137	5.7	2.4	285	5.5	2.3	121	6.0	2.3	759	7.5	2.1

For peer review only

	caring	136	6.1	2.5	286	6.1	2.1	119	5.8	2.6	756	7.5	2.1
	approachable	137	6.5	2.3	286	6.3	2.2	121	6.0	2.6	763	7.7	2.1
	comfort	137	6.1	2.5	286	5.8	2.3	121	5.7	2.5	761	7.5	2.2
	mean score	136	6.0	2.2	285	5.9	2.1	119	5.8	2.3	754	7.5	2.0
Formal with white coat	knowledgeable	131	7.2	2.1	284	6.6	1.9	102	7.4	2.0	764	8.2	1.9
	trustworthy	130	7.4	2.0	284	6.7	1.9	101	7.4	2.0	761	8.2	1.9
	caring	131	7.6	1.9	284	7.4	1.7	101	7.1	2.1	759	8.0	1.9
	approachable	131	7.8	1.8	284	7.4	1.8	102	7.2	2.1	758	8.1	1.9
	comfort	130	7.7	1.8	284	7.0	1.8	101	7.0	2.3	758	8.1	2.0
	mean score	130	7.5	1.8	284	7.0	1.6	101	7.2	1.9	754	8.1	1.8
Business suit	knowledgeable	131	5.5	2.6	295	5.3	2.2	110	5.2	2.5	755	7.4	2.3
	trustworthy	129	5.7	2.5	295	5.4	2.2	109	5.4	2.5	755	7.3	2.3
	caring	130	5.6	2.5	296	5.8	2.2	110	5.0	2.4	754	7.1	2.4
	approachable	128	5.8	2.6	296	5.8	2.3	110	5.4	2.5	753	7.2	2.4
	comfort	131	5.5	2.8	295	5.4	2.3	109	5.2	2.5	755	7.0	2.5
	mean score	128	5.6	2.4	295	5.5	2.1	108	5.2	2.2	751	7.2	2.2

For peer review only

Appendix D. Comparisons of patient preferences for physician attire by type of attire between countries

	Casual				Casual + White Coat				Scrubs			
Location Comparison	Mean difference	Simultaneous 95% confidence limits		sig	Mean difference	Simultaneous 95% confidence limits		sig	Mean difference	Simultaneous 95% confidence limits		sig
Italy-Japan	0.2049	-0.4354	0.8452		-0.1829	-0.714	0.3481		0.0028	-0.5287	0.5343	
Italy-US	-0.1454	-0.7192	0.4284		-0.5303	-1.007	-0.0535	***	-0.6422	-1.118	-0.1665	***
Italy-Swiss	-0.3137	-1.0902	0.4627		0.3128	-0.3236	0.9492		-0.3083	-0.9557	0.3391	
Japan-US	-0.3503	-0.7748	0.0742		-0.3473	-0.6987	0.004		-0.645	-0.9992	-0.2909	***
Japan-Swiss	-0.5186	-1.1923	0.155		0.4957	-0.0531	1.0445		-0.3111	-0.8751	0.253	
US-Swiss	-0.1683	-0.7791	0.4424		0.843	0.3466	1.3394	***	0.3339	-0.1779	0.8458	

	Scrubs + White Coat				Formal				Formal + White Coat				Suit			
Location Comparison	Mean difference	Simultaneous 95% confidence limits		sig	Mean difference	Simultaneous 95% confidence limits		sig	Mean difference	Simultaneous 95% confidence limits		sig	Mean difference	Simultaneous 95% confidence limits		sig
Italy-Japan	0.46551	-0.0631	0.99412		0.1275	-0.4215	0.6765		0.53538	0.05998	1.01079	***	0.101	-0.4955	0.6975	
Italy-US	-0.58969	-1.06635	-0.11303	***	-1.553	-2.0438	-1.0622	***	-0.57297	-0.99932	-0.14662	***	-1.5514	-2.0903	-1.0125	***
Italy-Swiss	-0.43477	-1.06551	0.19598		0.1381	-0.5231	0.7994		0.3136	-0.28188	0.90908		0.4027	-0.3337	1.139	
Japan-US	-1.0552	-1.39714	-0.71326	***	-1.6805	-2.0468	-1.3142	***	-1.10836	-1.42093	-0.79579	***	-1.6523	-2.0396	-1.2651	***
Japan-Swiss	-0.90028	-1.43652	-0.36404	***	0.0106	-0.5643	0.5856		-0.22178	-0.7419	0.29834		0.3017	-0.3321	0.9355	
US-Swiss	0.15492	-0.33018	0.64003		1.6911	1.1715	2.2108	***	0.88657	0.41088	1.36227	***	1.954	1.3741	2.534	***

Sig, ***: Statistically significant

For peer review only

Appendix E. **Comparisons of respondent opinions regarding importance, influence, and appropriateness of physician attire and white coats between countries**

	Important				Influence				Casual weekend			
Location Comparison	Mean difference	Simultaneous 95% confidence limits		sig	Mean difference	Simultaneous 95% confidence limits		sig	Mean difference	Simultaneous 95% confidence limits		sig
Italy-Japan	0.03935	-0.06317	0.14187		-0.29709	-0.40417	-0.19002	***	0.57316	0.46985	0.67648	***
Italy-US	0.05486	-0.03705	0.14677		-0.24249	-0.33851	-0.14646	***	-0.12125	-0.21387	-0.02864	***
Italy-Swiss	0.49847	0.37459	0.62235	***	0.44933	0.32	0.57867	***	-0.22814	-0.35336	-0.10292	***
Japan-US	0.01551	-0.05221	0.08323		0.05461	-0.01601	0.12522		-0.69442	-0.76271	-0.62613	***
Japan-Swiss	0.45912	0.35195	0.56629	***	0.74643	0.63466	0.8582	***	-0.8013	-0.90977	-0.69283	***
US-Swiss	0.44361	0.34655	0.54067	***	0.69182	0.59059	0.79306	***	-0.10689	-0.20522	-0.00856	***

	White coat office				White coat ER				White coat hospital				White coat any setting			
Location Comparison	Mean difference	Simultaneous 95% confidence limits		sig	Mean difference	Simultaneous 95% confidence limits		sig	Mean difference	Simultaneous 95% confidence limits		sig	Mean difference	Simultaneous 95% confidence limits		sig
Italy-Japan	0.33013	0.23509	0.42516	***	1.01157	0.91526	1.10788	***	0.599	0.51144	0.68657	***	0.57579	0.47487	0.67671	***
Italy-US	0.38806	0.30286	0.47326	***	0.72743	0.64117	0.81369	***	0.53173	0.45328	0.61019	***	0.43908	0.34865	0.52952	***
Italy-Swiss	0.65738	0.5425	0.77226	***	0.04456	-0.072	0.16113		0.27093	0.16489	0.37697	***	0.73863	0.61654	0.86072	***
Japan-US	0.05793	-0.00488	0.12075		-0.28414	-0.34798	-0.22031	***	-0.06727	-0.12529	-0.00925	***	-0.1367	-0.20354	-0.06986	***
Japan-Swiss	0.32726	0.22784	0.42667	***	-0.96701	-1.06811	-0.8659	***	-0.32808	-0.42003	-0.23612	***	0.16284	0.05703	0.26865	***
US-Swiss	0.26933	0.17926	0.35939	***	-0.68287	-0.77444	-0.59129	***	-0.26081	-0.34414	-0.17748	***	0.29954	0.20369	0.3954	***

Sig, ***: Statistically significant

Important: How my doctor dresses is important to me.

Influence: How my doctor dresses influences how happy I am with the care I receive.

Casual weekend: It is appropriate for a doctor to dress casually when seeing patients over the weekend.

White coat office: Doctors should wear a white coat when seeing patients in their office.

White coat ER: Doctors should wear a white coat when seeing patients in the emergency room.

White coat hospital: Doctors should wear a white coat when seeing patients in the hospital.

White coat any setting: Doctors should always wear a white coat when seeing patients in any setting.

For peer review only

Appendix F. **Composite scores by respondent gender**

Attire	Italy			Japan			Switzerland			United States		
	Male	Female	P	Male	Female	P	Male	Female	P	Male	Female	P
Casual	6.0	6.1	0.77	6.0	5.6	0.13	6.5	6.0	0.21	6.3	6.0	0.10
Casual with white coat	7.0	6.9	0.85	7.2	7.0	0.40	6.5	6.6	0.90	7.3	7.5	0.16
Formal	5.6	6.3	0.09	6.0	5.7	0.28	6.2	5.4	0.04*	7.6	7.4	0.23
Formal with white coat	7.5	7.6	0.73	7.0	7.0	0.77	7.3	7.1	0.55	8.1	8.1	0.94
Scrubs	6.5	6.9	0.34	6.8	6.8	0.93	7.2	6.9	0.38	7.4	7.5	0.71
Scrubs with white coat	7.3	6.9	0.26	6.5	6.6	0.60	7.5	7.5	0.96	7.6	7.7	0.41
Business suit	5.5	5.8	0.52	5.6	5.4	0.41	5.1	5.2	0.74	7.1	7.3	0.38

* Statistically significant

For peer review only

Appendix G. **Composite scores by respondent age**

**Attire**	**Italy**			**Japan**			**Switzerland**			**United States**		
	**Age ≥ 65**	**Age < 65**	**P**	**Age ≥ 65**	**Age < 65**	**P**	**Age ≥ 65**	**Age < 65**	**P**	**Age ≥ 65**	**Age < 65**	**P**
Casual	6.2	6.0	0.65	6.3	5.3	<.001*	5.9	6.4	0.37	6.5	6.0	0.01*
Casual with white coat	7.0	6.9	0.70	7.4	6.8	0.002*	6.2	6.6	0.56	7.6	7.3	0.10
Formal	6.0	6.0	0.86	6.4	5.4	<.001*	5.8	5.9	0.87	7.8	7.4	0.03*
Formal with white coat	7.7	7.5	0.41	7.3	6.7	0.002*	6.9	7.3	0.38	8.3	8.0	0.03*
Scrubs	6.7	6.7	0.99	7.0	6.5	0.01*	7.5	7.0	0.33	7.6	7.3	0.11
Scrubs with white coat	7.5	6.9	0.17	6.7	6.5	0.39	8.0	7.4	0.14	7.7	7.6	0.70
Business suit	5.4	5.7	0.65	6.1	5.0	<.001*	5.4	5.2	0.78	7.4	7.1	0.030*

* Statistically significant

For peer review only

BMJ Open

International Patient Preferences for Physician Attire: Results from Cross-Sectional Studies in Four Countries Across Three Continents

Journal:	*BMJ Open*
Manuscript ID	bmjopen-2022-061092.R1
Article Type:	Original research
Date Submitted by the Author:	22-Jun-2022
Complete List of Authors:	Houchens, Nathan; VA Ann Arbor Healthcare System; University of Michigan, Department of Medicine<br>Saint, Sanjay; VA Ann Arbor Healthcare System; University of Michigan, Department of Medicine<br>Petrilli, Christopher; NYU Langone Health<br>Kuhn, Latoya; VA Ann Arbor Healthcare System; University of Michigan, Department of Medicine<br>Ratz, David; VA Ann Arbor Healthcare System<br>De Lott, Lindsey; W K Kellogg Eye Center<br>Zollinger, Marc; Psychiatric University Hospital Zurich Department of Social and General Psychiatry Zurich West<br>Sax, Hugo; Inselspital University Hospital Bern, Department of Infectious Diseases<br>Kamata, Kazuhiro ; Niigata University Faculty of Medicine Graduate School of Medical and Dental Science, Department of Pediatrics; Fukushima Medical University Aizu Medical Center, Department of General Internal Medicine<br>Kuriyama, Akira; Kurashiki Central Hospital Emergency and Critical Care Center<br>Tokuda, Yasuharu; Muribushi Project for Okinawa Residency Programs, Department of Medicine<br>Fumagalli, Carlo; University of Florence, Department of Experimental and Clinical Medicine<br>Virgili, Gianni ; University of Florence, Department of Neurosciences, Psychology, Drug Research and Child Health (NEUROFARBA); Queen's University Belfast, Centre for Public Health<br>Fumagalli, Stefano; University of Florence, Department of Experimental and Clinical Medicine<br>Chopra, Vineet; University of Colorado, Department of Medicine
**Primary Subject Heading**:	Patient-centred medicine
Secondary Subject Heading:	Communication, Health policy
Keywords:	GENERAL MEDICINE (see Internal Medicine), Health policy < HEALTH SERVICES ADMINISTRATION & MANAGEMENT, Protocols & guidelines < HEALTH SERVICES ADMINISTRATION & MANAGEMENT

SCHOLARONE™
Manuscripts

I, the Submitting Author has the right to grant and does grant on behalf of all authors of the Work (as defined in the below author licence), an exclusive licence and/or a non-exclusive licence for contributions from authors who are: i) UK Crown employees; ii) where BMJ has agreed a CC-BY licence shall apply, and/or iii) in accordance with the terms applicable for US Federal Government officers or employees acting as part of their official duties; on a worldwide, perpetual, irrevocable, royalty-free basis to BMJ Publishing Group Ltd ("BMJ") its licensees and where the relevant Journal is co-owned by BMJ to the co-owners of the Journal, to publish the Work in this journal and any other BMJ products and to exploit all rights, as set out in our licence.

The Submitting Author accepts and understands that any supply made under these terms is made by BMJ to the Submitting Author unless you are acting as an employee on behalf of your employer or a postgraduate student of an affiliated institution which is paying any applicable article publishing charge ("APC") for Open Access articles. Where the Submitting Author wishes to make the Work available on an Open Access basis (and intends to pay the relevant APC), the terms of reuse of such Open Access shall be governed by a Creative Commons licence – details of these licences and which Creative Commons licence will apply to this Work are set out in our licence referred to above.

Other than as permitted in any relevant BMJ Author's Self Archiving Policies, I confirm this Work has not been accepted for publication elsewhere, is not being considered for publication elsewhere and does not duplicate material already published. I confirm all authors consent to publication of this Work and authorise the granting of this licence.

For peer review only

International Patient Preferences for Physician Attire: Results from Cross-Sectional Studies in Four Countries Across Three Continents

Nathan Houchens, MD[1,2]

Sanjay Saint, MD, MPH[1,2]

Christopher M. Petrilli, MD[3]

Latoya Kuhn, MPH[1,2]

David Ratz, MS[1]

Lindsey De Lott, MD, MS[4]

Marc Zollinger, MSc[5]

Hugo Sax, MD[6]

Kazuhiro Kamata, MD[7,8]

Akira Kuriyama, MD, MPH, PhD[9]

Yasuharu Tokuda, MD, MPH[10]

Carlo Fumagalli, MD[11]

Gianni Virgili, MD[12,13]

Stefano Fumagalli, MD, PhD[11]

Vineet Chopra, MD, MSc[14]

1 Medicine Service, Veterans Affairs Ann Arbor Healthcare System, Ann Arbor, Michigan, USA

2 Division of Hospital Medicine, Department of Medicine, University of Michigan, Ann Arbor, Michigan, USA

For peer review only - http://bmjopen.bmj.com/site/about/guidelines.xhtml

3 Division of General Internal Medicine and Clinical Innovation, Department of Medicine, NYU Langone Health, New York, New York, USA

4 Kellogg Eye Center, University of Michigan, Ann Arbor, Michigan, USA

5 University Hospital of Psychiatry Zurich (PUK), Zurich, Switzerland

6 Department of Infectious Diseases, Bern University Hospital and University of Bern, Bern, Switzerland

7 Department of Pediatrics, Niigata University Graduate School of Medical and Dental Sciences, Niigata, Japan

8 Department of General Internal Medicine, Aizu Medical Center, Fukushima Medical University, Fukushima, Japan

9 Emergency and Critical Care Center, Kurashiki Central Hospital, Okayama, Japan

10 Department of Medicine, Muribushi Project for Okinawa Residency Programs, Okinawa, Japan

11 Department of Experimental and Clinical Medicine, Geriatric Intensive Care Unit, University of Florence, Florence, Italy

12 Department of Neurosciences, Psychology, Drug Research and Child Health (NEUROFARBA), University of Florence and AOU, Florence, Italy

13 Centre for Public Health, Queen's University Belfast, Belfast, United Kingdom

14 Division of Hospital Medicine, Department of Medicine, University of Colorado, Denver, Colorado, USA

RUNNING TITLE: International Patient Preferences for Physician Attire

For peer review only

For peer review only - http://bmjopen.bmj.com/site/about/guidelines.xhtml

WORD COUNT: 4,000

CORRESPONDING AUTHOR:

Nathan Houchens, MD

Veterans Affairs Ann Arbor Healthcare System and University of Michigan

2215 Fuller Road, Mail Code 111

Ann Arbor, Michigan 48105

Email: nathanho@med.umich.edu

Phone: 734-845-5922

Fax: 734-845-5944

For peer review only

ABSTRACT

Objective: The patient-physician relationship impacts patients' experiences and health outcomes. Physician attire is a form of nonverbal communication that influences this relationship. Prior studies examining attire preferences suffered from heterogenous measurement and limited context. We thus performed a multi-center, cross-sectional study using a standardized survey instrument to compare patient preferences for physician dress in international settings.

Setting: 20 hospitals and healthcare practices in Italy, Japan, Switzerland, and the United States.

Participants: Convenience sample of 9,171 adult patients receiving care in academic hospitals, general medicine clinics, specialty clinics, and ophthalmology practices.

Primary and secondary outcome measures: The survey was randomized and included photographs of a male or female physician dressed in assorted forms of attire. The primary outcome measure was attire preference, comprised of composite ratings across five domains: how knowledgeable, trustworthy, caring, and approachable the physician appeared, and how comfortable the respondent felt. Secondary outcome measures included variation in preferences by country, physician type, and respondent characteristics.

Results: The highest rated forms of attire differed by country, although each most preferred attire with white coat. Low ratings were conferred on attire extremes (casual and business suit). Preferences were more uniform for certain physician types. For example, among all respondents, scrubs garnered the highest rating for emergency department physicians (44.2%) and surgeons (42.4%). However, attire preferences diverged for primary care and hospital physicians. All types of formal attire were more strongly preferred in the United States than elsewhere. Respondent age influenced preferences in Japan and the United States only.

For peer review only - http://bmjopen.bmj.com/site/about/guidelines.xhtml

Conclusions: Patients across a myriad of geographies, settings, and demographics harbor specific preferences for physician attire. Some preferences are nearly universal, whereas others vary substantially. As a one-size-fits-all dress policy is unlikely to reflect patient desires and expectations, a tailored approach should be sought that attempts to match attire to clinical context.

Keywords: Physician attire, dress, clothing, uniform, patient preferences, patient-physician relationship, nonverbal communication

ARTICLE SUMMARY

Strengths and Limitations of This Study

- With over 9,000 participants, this is the largest international study examining opinions on physician dress to date.
- Use of a standardized survey instrument allowed direct comparisons across diverse geographic regions, populations, physician types, and clinical contexts.
- Robust and careful survey design, including randomization and constant photographic features, mitigated bias and confounding.
- Comparative over-representation of the United States and convenience sampling may have contributed to disproportionate representation.
- The survey instrument used pre-defined Likert scales, which may not accurately reflect nuanced patient opinions, and which do not capture other elements of patient-physician interactions.

For peer review only

INTRODUCTION

Successful patient-physician relationships are founded on mutual respect, trust, confidence, and care. The strength of these connections can directly impact patients' experiences with healthcare, satisfaction, and important health outcomes such as adherence to treatment recommendations,[1,2] 30-day readmissions,[3] and mortality.[4] Patient-physician interactions are complex and dependent on multiple factors including social definitions and cultural norms. To ensure the highest quality care, it is essential to identify techniques that physicians may use to establish and maintain strong relationships with their unique individual patients while recognizing the influence of sociocultural context. From initial introductions, physicians employ verbal and nonverbal communication to form impressions and cultivate partnerships with their patients.[5]

The clothing worn by a physician is one form of nonverbal communication that may influence the patient-physician relationship. Physician attire is an important element in establishing patient confidence and trust,[6] enhancing patient comfort when discussing personal problems,[7-9] and shaping patient perceptions of physician professionalism,[6] intelligence,[10] and empathy.[11] Most prior scholarship has focused on a single geographic region, country, or clinical context (e.g., primary care clinic, hospital setting)[12-15] and has not considered the relative impacts of different physician specialties, contexts of care, geography, and patient factors such as age, education, and gender. Additionally, heterogeneity among prior studies, such as different sampling methodology and survey instruments, has made comparisons across different studies challenging.

The objective of this international, multi-center, cross-sectional study was to use a structured survey instrument to examine patient preferences for physician attire in different regions, countries, and continents. The survey instrument allowed direct comparisons among a

For peer review only - http://bmjopen.bmj.com/site/about/guidelines.xhtml

variety of cultures and contexts, thereby mitigating the heterogeneity of prior studies.[16-18] We report comparisons of data from five primary cross-sectional survey research studies conducted in Italy, Japan,[19] Switzerland,[20] and the United States.[21,22] Our aim was to identify common themes and differences of patient expectations for physician dress so that we may tailor attire and thus elevate the patient experience and optimize health outcomes.

METHODS

Study Design and Participants

We performed a survey-based study using a convenience sample of patients in 20 hospitals and healthcare practices in Italy, Japan, Switzerland, and the United States. These sites were selected based on our research networks and availability of clinicians who would serve as leads in their respective institutions. Sites included academic hospitals (general medicine wards, intensive care units), general medicine ambulatory clinics, specialty ambulatory clinics (dermatology, infectious disease, neurology, orthopedic surgery), and ophthalmology practices (**Table 1**). Data collection took place between June 2015 and October 2017.

At each participating healthcare location, the research team printed and randomly administered a survey instrument, targeting representative adult patients who were receiving clinical care at one of those sites. Participants were presented with a paper-based instrument of 22 questions that included photographs of either a male or female physician wearing various forms of attire and asked to rate their preferences. Respondents could request assistance with form completion from persons accompanying them.

All participants provided informed verbal consent. No identifying information was collected from participants that completed the study. Institutional permission for recruitment and

For peer review only - http://bmjopen.bmj.com/site/about/guidelines.xhtml

data collection was obtained from each site. The country-specific ethical review committees that reviewed and approved or deemed this project exempt from regulation were the University of Michigan Institutional Review Board (United States, HUM00085305); the Cantonal Ethics Review Board of Zurich, based on the Swiss law on research on humans (Switzerland, No. 60-2015); the ethics committee for Tokyo Joto Hospital (Japan, No. 2015-0001); and the ethics committee for Careggi University Hospital, according to the Declaration of Helsinki (Italy, CE 7123).

Procedures

The 22-item survey instrument was developed following a systematic review of the literature that examined the role of physician attire on the patient experience.[23] The survey instrument was developed and piloted by a multidisciplinary team to gather feedback and refine photographs, questions, rating scale, presentation order, and randomization scheme. Questions were translated into different languages for each country by interpreters at each site: Italian for Italy, Japanese for Japan, German for Switzerland (since the Swiss survey was conducted in Zurich), and English for the United States.

Each question referenced particular preferences and opinions of respondents in relation to photographs of medical providers wearing seven unique forms of attire. The forms of dress presented included: casual, casual with white coat, scrubs, scrubs with white coat, formal, formal with white coat, and business suit. Photographs were taken with attention paid to achieving constant physician facial expressions as well as consistent visual cues such as lighting, background, and pose. Photographs used at all study sites were identical with the following exceptions: In Switzerland, photographs of physicians in medical attire contextually appropriate

For peer review only - http://bmjopen.bmj.com/site/about/guidelines.xhtml

to the Swiss health system (i.e., white scrubs instead of blue scrubs) were used. All other photographic elements including physician models and other forms of attire were unchanged. In Japan, photographs of physicians of Japanese descent with slightly modified attire were used (**Appendix A**).

Each survey instrument had four sections. The first section showed a photograph of either a male or female physician wearing one of the seven unique forms of attire. To avoid biases such as anchoring, priming, order response, and gender conformity, 14 different versions of the survey instrument were created. The gender and attire of the first photograph seen by each respondent were randomized; all other sections of the survey were identical (**Appendix B**).

Measurements

Respondents were first asked to rate the standalone, randomized physician photograph using a 1 to 10 scale across five domains (i.e., how knowledgeable, trustworthy, caring, and approachable the physician appeared, and how comfortable the physician's appearance made the respondent feel), with a score of 10 representing the highest rating. Respondents were subsequently given seven photographs of the same physician wearing various forms of attire. Questions were asked regarding preference of attire in varied clinical settings (i.e., primary care, emergency department, hospital, surgery) and overall preference. To identify the influence of and respondent preferences for physician dress and white coats, a Likert scale ranging from 1 (strongly disagree) to 5 (strongly agree) was employed. Preferences for attire by respondent characteristics such as age, gender, education level, nationality, and number of unique physicians seen in the past year were collected. Unanswered questions and those with multiple responses were excluded.

For peer review only - http://bmjopen.bmj.com/site/about/guidelines.xhtml

The primary outcome of attire preference was calculated as the mean composite score of the five individual rating domains (i.e., knowledgeable, trustworthy, caring, approachable, and comfortable), with the highest score representing the most preferred form of attire. We also assessed variation in preferences for physician attire between countries, by physician type and clinical setting, and by respondent characteristics such as age and gender.

Statistical Analysis

Survey data were entered independently and in duplicate by the study teams. Respondents were not required to answer all questions; therefore, the denominator for each question varied. Data were reported as mean and standard deviation (SD) or N and percentage, where appropriate. Differences in the mean composite rating scores between countries were assessed using one-way ANOVA with the Tukey method for pairwise comparisons. Differences in mean composite score within country by sociodemographic factors were assessed using Student's T-tests. Differences between countries with respect to categorical responses were compared using Chi-squared tests. Statistical tests were assessed using p-value <0.05 considered significant. All analyses were performed using SAS V9.4 (SAS Inc, Cary, NC).

Patient and Public Involvement

Patients were not included in the design of the survey instrument, recruitment, or conduct of the study. Patients who participated did so anonymously, and therefore, the study team will be unable to disseminate the results to study participants.

Role of the Funding Source

For peer review only - http://bmjopen.bmj.com/site/about/guidelines.xhtml

This work was partially supported by a Swiss National Science Foundation grant (32003B_149474; PI, HS). Several investigators (SS, HS, MZ, VC, LD) received extramural funding for salary support. All authors had full access to all the data in the study and accept responsibility for the decision to submit for publication.

RESULTS

Characteristics of Study Sites and Participants

A total of 9,171 patients completed the survey instrument in outpatient and inpatient healthcare settings within a total of 20 hospitals or practices, 13 distinct geographic regions, 4 countries, and 3 continents. Patients were examined in age ranges of 18-64 years and 65 years or older. Patients aged 65 years or older comprised 36.0% of all respondents with substantial age variation across countries. For instance, those 65 years or older represented 48.5% of respondents in Japan, 35.6% in the United States, 27.8% in Italy, and 16.7% in Switzerland. Among all respondents, 44.9% were female, 39.6% had a college or graduate degree, and 26.6% had seen 6 or more physicians in the previous year. Characteristics of study sites are found in **Table 1**, and sociodemographic characteristics of respondents are described in **Table 2**.

Ratings of Attire Types by Country

Responses regarding patient preferences for physician attire varied by country. Formal attire with white coat received the highest ratings from respondents in Italy and the United States with mean composite scores of 7.5 (SD 1.8) and 8.1 (SD 1.8), respectively. Conversely, scrubs with white coat received the highest ratings in Switzerland (mean composite score of 7.5 [SD 1.7]) and casual attire with white coat in Japan (mean composite score of 7.1 [SD 1.8]). The forms of attire

For peer review only

For peer review only - http://bmjopen.bmj.com/site/about/guidelines.xhtml

that received the lowest mean composite ratings were business suit in Italy, Japan, and Switzerland with mean composite scores of 5.6 (SD 2.4), 5.5 (SD 2.1), and 5.2 (SD 2.2), respectively and casual attire in the United States with a mean composite score of 6.2 (SD 2.5). Within each country, composite scores for attire forms with white coat were higher than those for the corresponding forms without white coat, with only one exception (composite scores for scrubs and scrubs with white coat in Japan were 6.8 and 6.6, respectively). Ratings of different forms of attire by country are found in **Figure 1** and ratings of physician attire by domain are found in **Appendix C**.

Comparisons of Patient Preferences Between Countries

Preferences for Physician Attire by Type of Attire

Similarities between countries when comparing preferences for different types of physician attire were observed. For instance, there was complete concordance for all types of attire between the European countries of Italy and Switzerland. There was near complete concordance when comparing Italy and Japan, with the only statistically significant difference of Italy more strongly preferring formal attire with white coat compared with Japan (mean composite rating difference 0.54, simultaneous 95% confidence limits 0.06 to 1.01). Similarly, there was near complete concordance when comparing Switzerland and Japan, with the only significant difference of Switzerland more strongly preferring scrubs with white coat compared with Japan (mean composite rating difference 0.90, simultaneous 95% confidence limits 0.36 to 1.44). Among all types of attire, the form with the most concordance across countries was casual attire, with no between-country differences identified.

For peer review only - http://bmjopen.bmj.com/site/about/guidelines.xhtml

Just as ratings for physician attire varied by country, preferences for specific forms of attire also differed. For instance, the United States significantly more strongly preferred both forms of scrubs-based attire when compared with Italy and Japan, but not when compared with Switzerland. Additionally, the United States significantly more strongly preferred all forms of formal attire (i.e., formal attire with or without white coat and business suit) when compared with the other countries. These results are summarized in **Appendix D**.

Preferences for Physician Attire by Type of Physician

Photographs of either a male or female physician in seven different forms of attire (**Appendix B**) were shown, and respondents were asked to select which attire they preferred for different physician types. With respect to primary care physicians, respondents had varying preferences for attire. The highest rated forms in each country were formal attire with white coat in Italy (31.6%) and the United States (46.8%), casual attire with white coat in Japan (34.1%), and casual attire in Switzerland (24.4%). Heterogeneity in patient preferences was particularly noted in Switzerland with nearly equal preference given to casual attire, casual attire with white coat, and formal attire with white coat. The highest rated form of attire across all respondents was formal attire with white coat (40.1%).

With respect to hospital-based physicians, preferences again diverged. The highest rated forms in each country were scrubs with white coat in Italy (43.8%) and Switzerland (35.0%), casual attire with white coat in Japan (34.0%), and formal attire with white coat in the United States (37.6%). The highest rated form of attire across all respondents was formal attire with white coat (32.8%).

For peer review only - http://bmjopen.bmj.com/site/about/guidelines.xhtml

With respect to both emergency department physicians and surgeons, preferences were more uniform. Among all respondents across all countries, the most preferred form of attire was scrubs (44.2% for emergency department physicians, 42.4% for surgeons) followed by scrubs with white coat (30.4% for emergency department physicians, 25.4% for surgeons).

With respect to the most preferred form of attire overall, differences between countries were noted. The top forms of attire in each country were scrubs with white coat in Italy (41.7%) and Switzerland (31.5%) and formal attire with white coat in Japan (35.3%) and the United States (45.7%). The highest rated form of attire across all respondents was formal attire with white coat (38.6%). Among all respondents, 78.6% preferred some form of attire with a white coat, while 21.4% preferred a form without a white coat. **Table 3** shows preferred physician attire by physician type and clinical care setting.

Importance, Impact, and Appropriateness of Physician Attire and White Coats

Respondent opinions were sought using a Likert scale in which a score of 1 indicated "strongly disagree" and 5 "strongly agree." In response to the prompt "how my doctor dresses is important to me," mean scores were similar for Italy (3.55), Japan (3.51), and the United States (3.49) and lower for Switzerland (3.05) ($p<0.05$ for all 3 pairwise comparisons). In response to the prompt "how my doctor dresses influences how happy I am with the care I receive," mean scores for Italy were 2.92, Japan 3.22, Switzerland 2.47, and the United States 3.17 ($p<0.05$ for all pairwise comparisons except for Japan-United States). In response to the prompt "it is appropriate for a doctor to dress casually when seeing patients over the weekend," all countries differed with mean scores for Italy of 3.15, Japan 2.57, Switzerland 3.37, and the United States 3.27 ($p<0.05$ for all 6 pairwise comparisons).

For peer review only - http://bmjopen.bmj.com/site/about/guidelines.xhtml

With respect to perceptions of whether white coats should be worn by physicians in various settings, differences emerged. When considering whether physicians should wear a white coat when seeing patients in their office, mean scores for Italy were 3.92, Japan 3.59, Switzerland 3.27, and the United States 3.53 ($p<0.05$ for all pairwise comparisons except for Japan-United States). When asked if physicians should wear a white coat in the emergency department, mean scores for Italy were 4.06, Japan 3.05, Switzerland 4.02, and the United States 3.34 ($p<0.05$ for all pairwise comparisons except for Italy-Switzerland). When asked if physicians should wear a white coat in the hospital, all countries differed with mean scores for Italy of 4.16, Japan 3.57, Switzerland 3.89, and the United States 3.63 ($p<0.05$ for all 6 pairwise comparisons). In response to the prompt "doctors should always wear a white coat when seeing patients in any setting," all countries differed with mean scores for Italy of 3.56, Japan 2.99, Switzerland 2.82, and the United States 3.12 ($p<0.05$ for all 6 pairwise comparisons). These results are summarized in **Table 4** and **Appendix E**.

Comparisons of Patient Preferences Within Countries

Similarities and differences were identified when comparing preferences within countries based on respondent sociodemographic characteristics. When examining respondent gender, men and women rated different types of physician attire similarly within their respective countries. The only significant difference was that men rated formal attire more highly than women in Switzerland (male composite score 6.2, female composite score 5.4, $p=0.04$) (**Appendix F**). When comparing respondents aged 65 years and older with those between 18 and 64 years, there were no significant differences in composite scores for all types of physician attire in both Italy and Switzerland. In contrast, when compared with the younger cohort, respondents aged 65 years

For peer review only - http://bmjopen.bmj.com/site/about/guidelines.xhtml

and older rated casual attire, formal attire, formal attire with white coat, and business suit more highly in both Japan and the United States. When compared with the younger cohort, respondents aged 65 years and older rated casual attire with white coat and scrubs more highly in Japan, a finding that was not significant in the United States (**Appendix G**). There was no association between respondent preferences for physician attire and number of physicians seen in the prior year.

DISCUSSION

In this international, multi-center, cross-sectional study, we report preferences of 9,171 patients for physician attire across a variety of geographic regions, clinical contexts, physician types, and patient sociodemographic characteristics. We found that the highest rated form of physician attire differed across countries, but that all most strongly preferred a white coat with any attire. Respondents from the United States more strongly preferred all types of formal attire compared with those from Italy, Japan, and Switzerland. All countries more strongly preferred scrubs-based attire for emergency department physicians and surgeons. Taken together, these findings suggest that how a physician dresses has importance that varies around the world.

Our study adds to the existing literature by demonstrating that patients harbor expectations of how their physicians dress, and these expectations depend on sociocultural norms, context, and patient factors. In some clinical care contexts, preferences vary substantially. In others, they are nearly universal such as those for emergency department physicians and surgeons wearing scrubs-based attire. With some exceptions, patients tended to dislike extremes in attire such as casual or business suit. Finally, it was very common for patients to prefer their physicians wear a white coat, a historically traditional aspect of the physician's uniform and

For peer review only - http://bmjopen.bmj.com/site/about/guidelines.xhtml

what is often considered a symbol of the profession.[24] This was particularly evident when patient preferences for the underlying form of attire were split (e.g., primary care and hospital physicians).

Other studies exploring patient perceptions for physician attire have yielded a diverse and often conflicting array of findings, most of which are complicated by different measurement tools and outcomes. Consistent with our results, numerous studies across continents have identified a clear patient preference for white coats.[6,7,10,12,14,23,25-41] However, some studies reveal no significant preferences,[42-45] and others indicate that the white coat may even cause higher levels of tension in patients.[44] Some studies have shown that physician attire carries little importance with patients,[46-50] whereas others have shown it has a substantial impact on the patient experience,[30,51] congruent with our results. Literature differs on whether preferences for the white coat change after patients are educated about potential risk of microbial transmission, with some studies showing decreased preference[14,52] and another showing no change.[35] Studies examining attire in countries with bare-below-the-elbow policies have indicated near universal disdain for this infection prevention measure.[27,35] Some studies have shown preference for different forms of attire such as scrubs (e.g., specific circumstances like gastroenterology suites[18,53] and emergencies[5]) and informal attire,[54] and some have revealed no specific patient preferences.[52,55,56] Five studies noted that patient perceptions of compassion, professionalism, and credibility were not associated with a physician's dress.[25,32,57-59] Finally, some studies have demonstrated that attire is more important to patients who are older,[34,51,60] a finding we noted in Japan and the United States.

Studies conducted around the globe have repeatedly demonstrated that context is crucial when considering nonverbal cues like physician dress. Patient viewpoints are associated with a

For peer review only

For peer review only - http://bmjopen.bmj.com/site/about/guidelines.xhtml

variety of factors such as type of care delivered, type of physician, and even time of day. In one example, Switzerland has a defined healthcare uniform of white scrubs and white coat.[20] This relatively unique phenomenon likely caused patients to expect this form of attire and thus strongly prefer it to other forms. In another example from the United States, parents of children being evaluated in the pediatric emergency department were more likely to prefer physicians wearing scrubs but only if their children were experiencing a surgical emergency.[46] Likewise, in that same study, parents who visited the emergency department during the day shift preferred formal attire, whereas those who visited during the night preferred less formal attire.[46] Finally, preferences have also previously been shown to deviate from cultural norms or established national dress.[11,13,30,38] For instance, Batais and colleagues found that patients in family medicine clinics in Saudi Arabia were more likely to adhere to medical recommendations and return for subsequent care if the physician was dressed in Western garb;[60] yet this same population was significantly more willing to discuss personal issues such as psychological problems with a physician wearing Saudi national dress.[60] This finding of preferences that varied based on topic of conversation was noted in other studies as well.[9,10]

A number of strengths distinguish our study from others that have previously investigated patient preferences for physician attire. To our knowledge, this study of over 9,000 participants is the largest study examining opinions on physician dress to date. We employed a standardized survey instrument which allowed direct comparisons across diverse geography and contexts. Randomization of photograph sequence and instrument delivery reduced the risk of ordering, priming, and anchoring bias. We also used photographs containing physician models with identical postures, facial expressions, lighting, and background, all of which limited the confounding associated with previous studies utilizing models of different backgrounds and

For peer review only

For peer review only - http://bmjopen.bmj.com/site/about/guidelines.xhtml

appearances.[16-18,51,61] Finally, our findings have important policy implications for physician dress code in different care settings and areas of the world.

Our study also has limitations. Our physician models were young, slender, and either Caucasian or Asian, and as such were not representative of the various sociodemographic characteristics of physicians. Likewise, purposeful differences among survey instruments, including white scrubs instead of blue scrubs in the Switzerland survey and physician models of Japanese descent in the Japan survey, were introduced to ensure relevance. Our study over-represented the United States more so than Japan and the European countries, which could have contributed to skewed results and greater power in any comparison with the United States. For instance, this was particularly evident when examining attire for hospital physicians, in which the highest preference for formal attire with white coat was driven by United States respondents. Despite large sample sizes in Italy and Switzerland, only one clinical site was represented in each of these countries, and this may not fully represent the country. When feasible from our convenience sampling methodology, we surveyed multiple clinical sites because this approach likely achieved better representation of patients' preferences for different forms of attire in the respective countries. We did not obtain results from other regions including Africa, Australia, the Middle East, and South America, which could have contributed noteworthy input. Countries yielded different arrays of respondent sociodemographic characteristics such as age and education, which led to disproportionate representation among some groups. The survey instrument used Likert scales with pre-defined categories which may not accurately reflect nuanced patient opinions, and the clinical relevance of small but significant differences in these scales is unknown. The instrument did not capture or explore other elements of etiquette-based patient-physician interaction[62] such as introductions and smiles,[17,18,26,36,45] which are known to be

For peer review only

For peer review only - http://bmjopen.bmj.com/site/about/guidelines.xhtml

paramount for ensuring effective healthcare relationships. Our study did not compare the relative impacts of physician attire with these and other factors known to influence the patient-physician relationship such as health literacy,[63] communication skills,[64,65] and respect for patient autonomy.[64] Finally, the data from several of the individual country-specific studies have been previously published. However, this study is the first instance in which all data are compiled to allow for cross-national comparisons.

In conclusion, the effects of physician attire on the patient experience are complex and multilayered. Our findings suggest that one-size-fits-all physician attire policies which extend to all healthcare specialties and contexts are unlikely to reflect the desires and expectations of patients. Instead, our nuanced results that harness direct patient preferences may be used to inform local, regional, and national healthcare policymakers and leaders in their efforts to define physician uniforms. Given that preferences vary, a tailored approach should be sought that matches attire with acuity, setting, and context. This approach is most likely to cultivate the patient-physician relationship and in turn enhance patient satisfaction, trust, confidence, and health outcomes.

For peer review only

AUTHOR CONTRIBUTIONS

Conception and design of the work: SS, CMP, and VC. **Acquisition of the data**: SS, CMP, LK, VC, MZ, HS, and KK. **Analysis and interpretation of the data**: NH, SS, CMP, LK, DR, LD, MZ, HS, KK, AK, YT, CF, GV, SF, and VC. **Drafting of the manuscript**: NH, CMP, LK, DR, and VC. **Critical revision of the manuscript for important intellectual content**: NH, SS, CMP, LK, DR, LD, MZ, HS, KK, AK, YT, CF, GV, SF, and VC. **Accountable for all aspects of the work**: NH, SS, CMP, LK, DR, LD, MZ, HS, KK, AK, YT, CF, GV, SF, and VC. **Approval of the final manuscript**: NH, SS, CMP, LK, DR, LD, MZ, HS, KK, AK, YT, CF, GV, SF, and VC.

COMPETING INTERESTS

None declared.

FUNDING

This study was partially supported by a Swiss National Science Foundation grant (32003B_149474; PI, HS).

DATA SHARING STATEMENT

No data are available.

PATIENT CONSENT FOR PUBLICATION

Not required.

For peer review only

ETHICS APPROVAL

The country-specific ethical review committees that reviewed and approved or deemed this project exempt from regulation were the University of Michigan Institutional Review Board (United States, HUM00085305); the Cantonal Ethics Review Board of Zurich, based on the Swiss law on research on humans (Switzerland, No. 60-2015); the ethics committee for Tokyo Joto Hospital (Japan, No. 2015-0001); and the ethics committee for Careggi University Hospital, according to the Declaration of Helsinki (Italy, CE 7123).

AUTHOR NOTE

The views expressed in this article are those of the authors and do not necessarily reflect the position or policy of the Department of Veterans Affairs or the US government.

For peer review only

For peer review only - http://bmjopen.bmj.com/site/about/guidelines.xhtml

REFERENCES

1. Barbosa CD, Balp MM, Kulich K, et al. A literature review to explore the link between treatment satisfaction and adherence, compliance, and persistence. *Patient Prefer Adherence* 2012;6:39–48.
2. O'Malley AS, Forrest CB, Mandelblatt J. Adherence of low-income women to cancer screening recommendations. *J Gen Intern Med* 2002;17:144–54.
3. Boulding W, Glickman SW, Manary MP, et al. Relationship between patient satisfaction with inpatient care and hospital readmission within 30 days. *Am J Manag Care* 2011;17:41–8.
4. Doyle C, Lennox L, Bell D. A systematic review of evidence on the links between patient experience and clinical safety and effectiveness. *BMJ Open* 2013 Jan 3;3(1):e001570. doi: 10.1136/bmjopen-2012-001570. PMID: 23293244; PMCID: PMC3549241.
5. Van De Car W, Starostanko A, Wendling A. Rural Patient Preference for Physician Attire. *PRiMER* 2017 Feb 8;1:3. doi: 10.22454/PRiMER.2017.1.3. PMID: 32944689; PMCID: PMC7490192.
6. Gooden BR, Smith MJ, Tattersall SJ, et al. Hospitalised patients' views on doctors and white coats. *Med J Aust* 2001 Aug 20;175(4):219-22. doi: 10.5694/j.1326-5377.2001.tb143103.x. PMID: 11587285.
7. Rehman SU, Nietert PJ, Cope DW, et al. What to wear today? Effect of doctor's attire on the trust and confidence of patients. *Am J Med* 2005 Nov;118(11):1279-86. doi: 10.1016/j.amjmed.2005.04.026. PMID: 16271913.

For peer review only - http://bmjopen.bmj.com/site/about/guidelines.xhtml

8. Trowbridge RE, Pearson R. Impact of military physician rank and appearance on patient perceptions of clinical competency in a primary care setting. *Mil Med* 2013 Sep;178(9):994-1001. doi: 10.7205/MILMED-D-13-00197. PMID: 24005549.

9. Niederhauser A, Turner MD, Chauhan SP, et al. Physician attire in the military setting: does it make a difference to our patients? *Mil Med* 2009 Aug;174(8):817-20. doi: 10.7205/milmed-d-00-8409. PMID: 19743736.

10. Jennings JD, Ciaravino SG, Ramsey FV, et al. Physicians' Attire Influences Patients' Perceptions in the Urban Outpatient Orthopaedic Surgery Setting. *Clin Orthop Relat Res* 2016 Sep;474(9):1908-18. doi: 10.1007/s11999-016-4855-7. Epub 2016 Apr 26. PMID: 27116208; PMCID: PMC4965372.

11. Chung H, Lee H, Chang DS, et al. Doctor's attire influences perceived empathy in the patient-doctor relationship. *Patient Educ Couns* 2012 Dec;89(3):387-91. doi: 10.1016/j.pec.2012.02.017. Epub 2012 Mar 23. PMID: 22445730.

12. Mun HW, Kim JH, Ahn JH, et al. Patient's Preference on Neurosurgeon's Attire and Appearance: A Single Center Study in Korea Cross-Sectional Study. *Biomed Res Int* 2019 Apr 9;2019:3893049. doi: 10.1155/2019/3893049. PMID: 31093498; PMCID: PMC6481109.

13. Alzahrani HM, Mahfouz AA, Farag S, et al. Patients' perceptions and preferences for physicians' attire in hospitals in south western Saudi Arabia. *J Family Med Prim Care* 2020 Jun 30;9(6):3119-3123. doi: 10.4103/jfmpc.jfmpc_166_20. PMID: 32984183; PMCID: PMC7491852.

For peer review only

14. Zahrina AZ, Haymond P, Rosanna P, et al. Does the attire of a primary care physician affect patients' perceptions and their levels of trust in the doctor? *Malays Fam Physician* 2018 Dec 31;13(3):3-11. PMID: 30800227; PMCID: PMC6382088.

15. Verghese BG, Kalvehalli Kashinath S, Jadhav N, et al. Physician attire: physicians perspectives on attire in a community hospital setting among non-surgical specialties. *J Community Hosp Intern Med Perspect* 2020 Feb 10;10(1):1-5. doi: 10.1080/20009666.2020.1718478. PMID: 32128050; PMCID: PMC7034488.

16. Au S, Khandwala F, Stelfox HT. Physician attire in the intensive care unit and patient family perceptions of physician professional characteristics. *JAMA Intern Med* 2013 Mar 25;173(6):465-7. doi: 10.1001/jamainternmed.2013.2732. PMID: 23420343.

17. Lill MM, Wilkinson TJ. Judging a book by its cover: descriptive survey of patients' preferences for doctors' appearance and mode of address. *BMJ* 2005 Dec 24;331(7531):1524-7. doi: 10.1136/bmj.331.7531.1524. PMID: 16373739; PMCID: PMC1322253.

18. Sotgiu G, Nieddu P, Mameli L, et al. Evidence for preferences of Italian patients for physician attire. *Patient Prefer Adherence* 2012;6:361-7. doi: 10.2147/PPA.S29587. Epub 2012 Apr 27. PMID: 22573935; PMCID: PMC3346157.

19. Kamata K, Kuriyama A, Chopra V, et al. Patient Preferences for Physician Attire: A Multicenter Study in Japan. *J Hosp Med* 2020 Apr 1;15(4):204-210. doi: 10.12788/jhm.3350. Epub 2020 Feb 11. PMID: 32118558.

20. Zollinger M, Houchens N, Chopra V, et al. Understanding patient preference for physician attire in ambulatory clinics: a cross-sectional observational study. *BMJ Open*

For peer review only

2019 May 9;9(5):e026009. doi: 10.1136/bmjopen-2018-026009. PMID: 31072853; PMCID: PMC6528053.

21. Petrilli CM, Saint S, Jennings JJ, et al. Understanding patient preference for physician attire: a cross-sectional observational study of 10 academic medical centres in the USA. *BMJ Open* 2018 May 29;8(5):e021239. doi: 10.1136/bmjopen-2017-021239. PMID: 29844101; PMCID: PMC5988098.

22. De Lott LB, Panarelli JF, Samimi D, et al. Patient Preferences for Physician Attire in Ophthalmology Practices. *J Acad Ophthalmol* 2019 Jan;11(1):e36-e42. doi: 10.1055/s-0039-1688913. PMID: 31475243; PMCID: PMC6716523.

23. Petrilli CM, Mack M, Petrilli JJ, et al. Understanding the role of physician attire on patient perceptions: a systematic review of the literature--targeting attire to improve likelihood of rapport (TAILOR) investigators. *BMJ Open* 2015 Jan 19;5(1):e006578. doi: 10.1136/bmjopen-2014-006578. PMID: 25600254; PMCID: PMC4312788.

24. Karnieli-Miller O, Frankel RM, Inui TS. Cloak of compassion, or evidence of elitism? an empirical analysis of white coat ceremonies. *Med Educ* 2013 Jan;47(1):97-108. doi: 10.1111/j.1365-2923.2012.04324.x. PMID: 23278829.

25. Al-Ghobain MO, Al-Drees TM, Alarifi MS, et al. Patients' preferences for physicians' attire in Saudi Arabia. *Saudi Med J* 2012 Jul;33(7):763-7. PMID: 22821311.

26. Major K, Hayase Y, Balderrama D, et al. Attitudes regarding surgeons' attire. *Am J Surg* 2005 Jul;190(1):103-6. doi: 10.1016/j.amjsurg.2005.04.003. PMID: 15972180.

27. Meshkat B, Bass GA, Matcovici M, et al. Patients attitude towards surgeons attire in Our Lady of Lourdes Hospital Drogheda. *Int J Health Policy Manag* 2015 Feb 15;4(4):217-20. doi: 10.15171/ijhpm.2015.31. PMID: 25844382; PMCID: PMC4380563.

For peer review only - http://bmjopen.bmj.com/site/about/guidelines.xhtml

28. Lands VW, Malige A, Nwachuku CO, et al. The Effect of an Orthopedic Hand Surgeon's Attire on Patient Confidence and Trust. *Hand (N Y)* 2019 Sep;14(5):675-683. doi: 10.1177/1558944717750918. Epub 2018 Jan 18. PMID: 29343100; PMCID: PMC6759972.

29. Yamada Y, Takahashi O, Ohde S, et al. Patients' preferences for doctors' attire in Japan. *Intern Med* 2010;49(15):1521-6. doi: 10.2169/internalmedicine.49.3572. Epub 2010 Aug 2. PMID: 20686283.

30. Aldrees T, Alsuhaibani R, Alqaryan S, et al. Physicians' attire. Parents preferences in a tertiary hospital. *Saudi Med J* 2017 Apr;38(4):435-439. doi: 10.15537/smj.2017.4.15853. PMID: 28397953; PMCID: PMC5447199.

31. Al Amry KM, Al Farrah M, Ur Rahman S, et al. Patient perceptions and preferences of physicians' attire in Saudi primary healthcare setting. *J Community Hosp Intern Med Perspect* 2018 Dec 11;8(6):326-330. doi: 10.1080/20009666.2018.1551026. PMID: 30559939; PMCID: PMC6292367.

32. Carugno J, Timmons D, Grady M, et al. Impact of physician attire on patients' impression of their gynecologist: Results from a large single-center survey analysis. *Eur J Obstet Gynecol Reprod Biol* 2020 Nov;254:266-270. doi: 10.1016/j.ejogrb.2020.09.040. Epub 2020 Sep 23. PMID: 33035822.

33. Maruani A, Léger J, Giraudeau B, et al. Effect of physician dress style on patient confidence. *J Eur Acad Dermatol Venereol* 2013 Mar;27(3):e333-7. doi: 10.1111/j.1468-3083.2012.04665.x. Epub 2012 Aug 9. PMID: 22882283.

For peer review only

For peer review only - http://bmjopen.bmj.com/site/about/guidelines.xhtml

34. Kurihara H, Maeno T, Maeno T. Importance of physicians' attire: factors influencing the impression it makes on patients, a cross-sectional study. *Asia Pac Fam Med* 2014 Jan 8;13(1):2. doi: 10.1186/1447-056X-13-2. PMID: 24397871; PMCID: PMC3890493.

35. Landry M, Dornelles AC, Hayek G, et al. Patient Preferences for Doctor Attire: The White Coat's Place in the Medical Profession. *Ochsner J* 2013 Fall;13(3):334-42. PMID: 24052762; PMCID: PMC3776508.

36. Matsui D, Cho M, Rieder MJ. Physicians' attire as perceived by young children and their parents: the myth of the white coat syndrome. *Pediatr Emerg Care* 1998 Jun;14(3):198-201. doi: 10.1097/00006565-199806000-00006. PMID: 9655662.

37. Iram S, Prakash WD, Ali MJ, et al. Preferences of ophthalmic plastics patients and their caregivers toward the doctors' attire and initial communications: A tertiary eye care study. *Indian J Ophthalmol* 2016 Jun;64(6):448-51. doi: 10.4103/0301-4738.187674. PMID: 27488153; PMCID: PMC4991175.

38. Chang DS, Lee H, Lee H, et al. What to wear when practicing oriental medicine: patients' preferences for doctors' attire. *J Altern Complement Med* 2011 Aug;17(8):763-7. doi: 10.1089/acm.2010.0612. Epub 2011 Jul 1. PMID: 21721926.

39. Gallagher J, Waldron Lynch F, Stack J, et al. Dress and address: patient preferences regarding doctor's style of dress and patient interaction. *Ir Med J* 2008 Jul-Aug;101(7):211-3. PMID: 18807811.

40. Gherardi G, Cameron J, West A, et al. Are we dressed to impress? A descriptive survey assessing patients' preference of doctors' attire in the hospital setting. *Clin Med (Lond)* 2009 Dec;9(6):519-24. doi: 10.7861/clinmedicine.9-6-519. PMID: 20095290; PMCID: PMC4952286.

For peer review only - http://bmjopen.bmj.com/site/about/guidelines.xhtml

41. McNaughton-Filion L, Chen JS, Norton PG. The physician's appearance. *Fam Med* 1991 Mar-Apr;23(3):208-11. PMID: 2016013.

42. La Rosa M, Spencer N, Abdelwahab M, et al. The Effect of Wearing White Coats on Patients' Appreciation of Physician Communication during Postpartum Rounds: A Randomized Controlled Trial. *Am J Perinatol* 2019 Jan;36(1):62-66. doi: 10.1055/s-0038-1660470. Epub 2018 Jun 8. PMID: 29883984.

43. Cha A, Hecht BR, Nelson K, et al. Resident physician attire: does it make a difference to our patients? *Am J Obstet Gynecol* 2004 May;190(5):1484-8. doi: 10.1016/j.ajog.2004.02.022. PMID: 15167876.

44. Ikusaka M, Kamegai M, Sunaga T, et al. Patients' attitude toward consultations by a physician without a white coat in Japan. *Intern Med* 1999 Jul;38(7):533-6. doi: 10.2169/internalmedicine.38.533. PMID: 10435357.

45. Varnado-Sullivan P, Larzelere M, Solek K, et al. The Impact of Physician Demographic Characteristics on Perceptions of Their Attire. *Fam Med* 2019 Oct 4;51(9):737-741. doi: 10.22454/FamMed.2019.650493. Epub 2019 Aug 26. PMID: 31465109.

46. Gonzalez Del Rey JA, Paul RI. Preferences of parents for pediatric emergency physicians' attire. *Pediatr Emerg Care* 1995 Dec;11(6):361-4. doi: 10.1097/00006565-199512000-00007. PMID: 8751171.

47. Li SF, Haber M. Patient attitudes toward emergency physician attire. *J Emerg Med* 2005 Jul;29(1):1-3. doi: 10.1016/j.jemermed.2004.12.014. PMID: 15960999.

48. Menahem S, Shvartzman P. Is our appearance important to our patients? *Fam Pract* 1998 Oct;15(5):391-7. doi: 10.1093/fampra/15.5.391. PMID: 9848422.

For peer review only - http://bmjopen.bmj.com/site/about/guidelines.xhtml

49. Friis R, Tilles J. Patients' preferences for resident physician dress style. *Fam Pract Res J* 1988 Fall-Winter;8(1):24-31. PMID: 3239442.

50. McLean C, Patel P, Sullivan C, et al. Patients' perception of military doctors in fracture clinics--does the wearing of uniform make a difference? *J R Nav Med Serv* 2005;91(1):45-7. PMID: 15986841.

51. McKinstry B, Wang JX. Putting on the style: what patients think of the way their doctor dresses. *Br J Gen Pract* 1991 Jul;41(348):270, 275-8. PMID: 1747264; PMCID: PMC1371685.

52. Hueston WJ, Carek SM. Patients' preference for physician attire: a survey of patients in family medicine training practices. *Fam Med* 2011 Oct;43(9):643-7. PMID: 22002776.

53. Clark M, Shuja A, Thomas A, et al. Patients' perceptions of gastroenterologists' attire in the clinic and endoscopy suite. *Ann Gastroentero*l 2018 Mar-Apr;31(2):237-240. doi: 10.20524/aog.2017.0223. Epub 2017 Dec 22. PMID: 29507472; PMCID: PMC5825955.

54. Reddy R. Slippers and a white coat? (Hawai'i physician attire study). *Hawaii Med J* 2009 Dec;68(11):284-5. PMID: 20034257.

55. Fischer RL, Hansen CE, Hunter RL, et al. Does physician attire influence patient satisfaction in an outpatient obstetrics and gynecology setting? *Am J Obstet Gynecol* 2007 Feb;196(2):186.e1-5. doi: 10.1016/j.ajog.2006.09.043. PMID: 17306675.

56. Longmuir S, Gilbertson A, Pfeifer W, et al. Pediatric ophthalmology attire: should we wear a white coat? *Insight* 2010 Oct-Dec;35(4):11-3. PMID: 21189797.

57. Azhar A, Tanco K, Haider A, et al. Challenging the status quo of physician attire in the palliative care setting. *Oncologist* 2020 Jul;25(7):627-637. doi:

For peer review only

For peer review only - http://bmjopen.bmj.com/site/about/guidelines.xhtml

10.1634/theoncologist.2019-0568. Epub 2020 Feb 19. PMID: 32073181; PMCID: PMC7356715.

58. Traeger AC, Skinner IW, Hübscher M, et al. What you wear does not affect the credibility of your treatment: A blinded randomized controlled study. *Patient Educ Couns* 2017 Jan;100(1):104-111. doi: 10.1016/j.pec.2016.08.009. Epub 2016 Aug 8. PMID: 27522250.

59. Boon D, Wardrope J. What should doctors wear in the accident and emergency department? Patients' perception. *J Accid Emerg Med* 1994 Sep;11(3):175-7. doi: 10.1136/emj.11.3.175. PMID: 7804584; PMCID: PMC1342426.

60. Batais MA. Patients' attitudes toward the attire of male physicians: a single-center study in Saudi Arabia. *Ann Saudi Med* 2014 Sep-Oct;34(5):383-9. doi: 10.5144/0256-4947.2014.383. PMID: 25827694; PMCID: PMC6074558.

61. Kocks JW, Lisman-van Leeuwen Y, Berkelmans PG. De kleren maken de dokter--meer vertrouwen in netter geklede huisarts [Clothing make the doctor--patients have more confidence in a smartly dressed GP]. *Ned Tijdschr Geneeskd* 2010;154(51-52):A2898. Dutch. PMID: 21211081.

62. Kahn MW. Etiquette-based medicine. *N Engl J Med* 2008 May 8;358(19):1988-9. doi: 10.1056/NEJMp0801863. PMID: 18463374.

63. Liang CY, Wang KY, Hwang SJ, et al. Factors affecting the physician-patient relationship of older veterans with inadequate health literacy: an observational study. *Br J Gen Pract* 2013 May;63(610):e354-60. doi: 10.3399/bjgp13X667222. PMID: 23643234; PMCID: PMC3635582.

For peer review only - http://bmjopen.bmj.com/site/about/guidelines.xhtml

64. Hamelin ND, Nikolis A, Armano J, et al. Evaluation of factors influencing confidence and trust in the patient-physician relationship: a survey of patient in a hand clinic. *Chir Main* 2012 Apr;31(2):83-90. doi: 10.1016/j.main.2012.01.005. Epub 2012 Feb 3. PMID: 22365321.

65. Ha JF, Longnecker N. Doctor-patient communication: a review. *Ochsner J* 2010 Spring;10(1):38-43. PMID: 21603354; PMCID: PMC3096184.

For peer review only

***Table 1*: Characteristics of participating study sites**

Country	Dates of Data Collection	Types of Outpatient Clinics	Clinical Setting(s)	Hospitals, Practices	Geographic Regions Sampled	Surveys Completed
Italy	10/26/2015-10/21/2016	Infectious Disease, Ophthalmology, Geriatric Intensive Care Unit	Outpatient and Inpatient	1	1[*]	958
Japan	12/01/2015-10/30/2017	General Medicine, Medicine Specialties, Orthopedic Surgery	Outpatient and Inpatient	4	3[†]	2020
Switzerland	06/15/2015-10/31/2016	Dermatology, Infectious Disease, Neurology	Outpatient	1	1[‡]	834
United States (US)[§]	06/01/2015-10/31/2016	General Medicine, Medicine Specialties	Outpatient and Inpatient	10	4[**]	4062
		Ophthalmology	Outpatient	4	3[††]	1297

[*] One site in the Tuscany region
[†] Two sites in the Kantō region; one site in the Kansai region; one site in the Chūgoku region
[‡] One site in the Canton of Zurich
[§] Geographic regions of the US include Northeast, Midwest, South, and West
[**] Three sites in the Midwest, three sites in the South, two sites in the Northeast, two sites in the West
[††] Two sites in the Midwest, one site in the Northeast, one site in the West

For peer review only

Table 2: **Sociodemographic information**

	**Italy (n=958)**	**Japan (n=2020)**	**Switzerland (n=834)**	**United States (n=5359)**	**Total (n=9171)**
**Age**	n=928	n=2010	n=812	n=5279	n=9029
18-25	61 (6.6%)	67 (3.3%)	50 (6.2%)	241 (4.6%)	419 (4.6%)
26-34	89 (9.6%)	162 (8.1%)	93 (11.5%)	464 (8.8%)	808 (9.0%)
35-54	310 (33.4%)	461 (22.9%)	341 (42.0%)	1299 (24.6%)	2411 (26.7%)
55-64	210 (22.6%)	345 (17.2%)	192 (23.6%)	1393 (26.4%)	2140 (23.7%)
≥65	258 (27.8%)	975 (48.5%)	136 (16.7%)	1882 (35.6%)	3251 (36.0%)
**Gender**	n=905	n=2011	n=806	n=5194	n=8916
Female	471 (52.0%)	1040 (51.7%)	304 (37.7%)	2184 (42.0%)	3999 (44.9%)
Male	434 (48.0%)	971 (48.3%)	502 (62.3%)	3010 (58.0%)	4917 (55.1%)
**Education**	n=919	n=2010	n=808	n=5247	n=8984
Less than high school	237 (25.8%)	243 (12.1%)	368 (45.5%)	146 (2.8%)	994 (11.1%)
High school diploma	416 (45.3%)	1236 (61.5%)	82 (10.2%)	2691 (51.3%)	4425 (49.3%)
College degree	77 (8.4%)	487 (24.2%)	340 (42.1%)	1490 (28.4%)	2394 (26.6%)
Graduate degree	189 (20.5%)	44 (2.2%)	18 (2.2%)	920 (17.5%)	1171 (13.0%)
**Number of unique physicians seen in the past year**	n=928	n=2009	n=810	n=5265	n=9012
0	76 (8.2%)	38 (1.9%)	13 (1.6%)	51 (1.0%)	178 (2.0%)
1	126 (13.6%)	140 (7.0%)	83 (10.2%)	377 (7.2%)	726 (8.1%)
2	199 (21.4%)	373 (18.5%)	165 (20.4%)	769 (14.6%)	1506 (16.7%)
3	188 (20.3%)	512 (25.5%)	203 (25.1%)	940 (17.9%)	1843 (20.4%)
4	112 (12.1%)	359 (17.9%)	126 (15.6%)	824 (15.6%)	1421 (15.8%)
5	84 (9.0%)	225 (11.2%)	57 (7.0%)	571 (10.8%)	937 (10.4%)
≥6	143 (15.4%)	362 (18.0%)	163 (20.1%)	1733 (32.9%)	2401 (26.6%)

For peer review only - http://bmjopen.bmj.com/site/about/guidelines.xhtml

***Table 3*: Preferred physician attire by physician type and care setting**

Physician Type	Attire	Italy	Japan	Switzerland	United States	Total
Primary Care Physician	Casual	103 (11.0%)	33 (1.6%)	199 (24.4%)	158 (3.0%)	493 (5.5%)
	Casual with white coat	165 (17.6%)	682 (34.1%)	183 (22.4%)	518 (9.9%)	1548 (17.2%)
	Scrubs	61 (6.5%)	188 (9.4%)	90 (11.0%)	238 (4.6%)	577 (6.4%)
	Scrubs with white coat	126 (13.5%)	357 (17.9%)	78 (9.6%)	742 (14.2%)	1303 (14.5%)
	Formal	128 (13.7%)	49 (2.5%)	73 (8.9%)	787 (15.0%)	1037 (11.6%)
	Formal with white coat	296 (31.6%)	669 (33.4%)	188 (23.0%)	2451 (46.8%)	3604 (40.1%)
	Business suit	57 (6.1%)	22 (1.1%)	6 (0.7%)	340 (6.5%)	425 (4.7%)
Emergency Department Physician	Casual	36 (3.9%)	42 (2.1%)	31 (3.8%)	63 (1.2%)	172 (1.9%)
	Casual with white coat	89 (9.6%)	206 (10.3%)	65 (8.0%)	298 (5.7%)	658 (7.3%)
	Scrubs	343 (37.2%)	1131 (56.5%)	382 (46.9%)	2108 (40.2%)	3964 (44.2%)
	Scrubs with white coat	324 (35.1%)	354 (17.7%)	271 (33.3%)	1784 (34.1%)	2733 (30.4%)
	Formal	16 (1.7%)	61 (3.0%)	8 (1.0%)	134 (2.6%)	219 (2.4%)
	Formal with white coat	105 (11.4%)	204 (10.2%)	52 (6.4%)	793 (15.1%)	1154 (12.9%)
	Business suit	10 (1.1%)	5 (0.2%)	5 (0.6%)	60 (1.1%)	80 (0.9%)
Hospital Physician	Casual	25 (2.7%)	19 (1.0%)	33 (4.1%)	68 (1.3%)	145 (1.6%)
	Casual with white coat	98 (10.6%)	680 (34.0%)	138 (17.0%)	435 (8.3%)	1351 (15.1%)
	Scrubs	176 (19.1%)	162 (8.1%)	203 (25.0%)	594 (11.4%)	1135 (12.7%)
	Scrubs with white coat	404 (43.8%)	444 (22.2%)	285 (35.0%)	1600 (30.7%)	2733 (30.5%)
	Formal	17 (1.8%)	26 (1.3%)	20 (2.4%)	346 (6.6%)	409 (4.6%)
	Formal with white coat	189 (20.5%)	660 (33.0%)	129 (15.9%)	1964 (37.6%)	2942 (32.8%)
	Business suit	14 (1.5%)	9 (0.4%)	5 (0.6%)	212 (4.1%)	240 (2.7%)
Surgeon	Casual	32 (3.5%)	13 (0.6%)	17 (2.1%)	37 (0.7%)	99 (1.1%)
	Casual with white coat	85 (9.2%)	238 (11.9%)	44 (5.4%)	179 (3.4%)	546 (6.1%)
	Scrubs	289 (31.2%)	942 (47.1%)	345 (42.6%)	2224 (42.5%)	3800 (42.4%)
	Scrubs with white coat	302 (32.6%)	501 (25.0%)	272 (33.6%)	1202 (23.0%)	2277 (25.4%)
	Formal	37 (4.0%)	35 (1.8%)	17 (2.1%)	192 (3.7%)	281 (3.1%)
	Formal with white coat	155 (16.8%)	266 (13.3%)	108 (13.3%)	1102 (21.1%)	1631 (18.2%)
	Business suit	25 (2.7%)	6 (0.3%)	7 (0.9%)	291 (5.6%)	329 (3.7%)
Overall	Casual	20 (2.2%)	17 (0.9%)	46 (5.8%)	70 (1.4%)	153 (1.7%)

For peer review only - http://bmjopen.bmj.com/site/about/guidelines.xhtml

	Casual with white coat	94 (10.2%)	606 (30.3%)	136 (17.0%)	367 (7.1%)	1203 (13.5%)
	Scrubs	146 (15.8%)	203 (10.1%)	205 (25.6%)	390 (7.5%)	944 (10.6%)
	Scrubs with white coat	385 (41.7%)	436 (21.8%)	252 (31.5%)	1289 (24.8%)	2362 (26.5%)
	Formal	25 (2.7%)	26 (1.3%)	22 (2.7%)	448 (8.6%)	521 (5.9%)
	Formal with white coat	235 (25.5%)	707 (35.3%)	131 (16.4%)	2370 (45.7%)	3443 (38.6%)
	Business suit	18 (1.9%)	7 (0.3%)	8 (1.0%)	255 (4.9%)	288 (3.2%)

For peer review only

For peer review only - http://bmjopen.bmj.com/site/about/guidelines.xhtml

***Table 4*: Respondent opinions regarding importance, influence, and appropriateness of physician attire and white coats**

		**Italy**	**Japan**	**Switzerland**	**United States**	**Total**
How my doctor dresses is important to me.	Strongly disagree	60 (6.4%)	67 (3.3%)	110 (13.4%)	222 (4.2%)	459 (5.1%)
	Disagree	87 (9.4%)	280 (13.9%)	151 (18.4%)	531 (10.0%)	1049 (11.6%)
	Neither agree nor disagree	220 (23.7%)	430 (21.4%)	260 (31.8%)	1603 (30.2%)	2513 (27.7%)
	Agree	410 (44.1%)	1031 (51.3%)	185 (22.6%)	2303 (43.5%)	3929 (43.4%)
	Strongly agree	153 (16.4%)	202 (10.1%)	113 (13.8%)	641 (12.1%)	1109 (12.2%)
	Mean*	3.55	3.51	3.05	3.49	
How my doctor dresses influences how happy I am with the care I receive.	Strongly disagree	132 (14.3%)	124 (6.2%)	223 (27.3%)	334 (6.3%)	813 (9.0%)
	Disagree	209 (22.6%)	396 (19.7%)	235 (28.8%)	851 (16.1%)	1691 (18.7%)
	Neither agree nor disagree	250 (27.0%)	536 (26.7%)	171 (20.9%)	2088 (39.5%)	3045 (33.7%)
	Agree	263 (28.5%)	812 (40.5%)	124 (15.2%)	1633 (30.9%)	2832 (31.3%)
	Strongly agree	70 (7.6%)	138 (6.9%)	64 (7.8%)	384 (7.2%)	656 (7.3%)
	Mean*	2.92	3.22	2.47	3.17	
It is appropriate for a doctor to dress casually when seeing patients over the weekend.	Strongly disagree	81 (8.7%)	209 (10.4%)	104 (12.8%)	182 (3.5%)	576 (6.4%)
	Disagree	213 (22.9%)	837 (41.7%)	139 (17.2%)	955 (18.1%)	2144 (23.7%)
	Neither agree nor disagree	218 (23.4%)	613 (30.5%)	147 (18.2%)	1761 (33.3%)	2739 (30.3%)
	Agree	326 (35.1%)	300 (15.0%)	189 (23.4%)	2047 (38.7%)	2862 (31.7%)
	Strongly agree	92 (9.9%)	48 (2.4%)	230 (28.4%)	340 (6.4%)	340 (7.9%)
	Mean*	3.15	2.57	3.37	3.27	
Doctors should wear a white coat when seeing patients in their office.	Strongly disagree	20 (2.2%)	48 (2.4%)	108 (13.2%)	84 (1.6%)	260 (2.9%)
	Disagree	47 (5.1%)	226 (11.2%)	132 (16.1%)	552 (10.4%)	957 (10.6%)
	Neither agree nor disagree	139 (14.9%)	437 (21.7%)	170 (20.8%)	1698 (32.1%)	2444 (27.0%)
	Agree	504 (54.1%)	1085 (54.0%)	251 (30.7%)	2361 (44.7%)	4201 (46.4%)
	Strongly agree	221 (23.7%)	214 (10.7%)	157 (19.2%)	593 (11.2%)	1185 (13.1%)
	Mean*	3.92	3.59	3.27	3.53	
Doctors should wear a white coat when seeing patients in the	Strongly disagree	15 (1.6%)	102 (5.1%)	47 (5.8%)	111 (2.1%)	275 (3.0%)
	Disagree	36 (3.8%)	541 (27.0%)	56 (6.9%)	828 (15.6%)	1461 (16.2%)
	Neither agree nor disagree	115 (12.3%)	623 (31.1%)	75 (9.2%)	1952 (36.9%)	2765 (30.6%)

For peer review only - http://bmjopen.bmj.com/site/about/guidelines.xhtml

emergency department.	Agree	480 (51.2%)	628 (31.3%)	294 (36.0%)	1973 (37.3%)	3375 (37.3%)
	Strongly agree	291 (31.1%)	110 (5.5%)	343 (42.1%)	426 (8.1%)	1170 (12.9%)
	Mean*	4.06	3.05	4.02	3.34	
Doctors should wear a white coat when seeing patients in the hospital	Strongly disagree	13 (1.4%)	45 (2.2%)	50 (6.1%)	65 (1.2%)	173 (1.9%)
	Disagree	19 (2.0%)	236 (11.7%)	45 (5.5%)	401 (7.6%)	701 (7.7%)
	Neither agree nor disagree	83 (8.8%)	441 (22.0%)	128 (15.7%)	1507 (28.5%)	2159 (23.9%)
	Agree	509 (54.3%)	1114 (55.4%)	311 (38.2%)	2756 (52.1%)	4690 (51.8%)
	Strongly agree	314 (33.5%)	174 (8.7%)	281 (34.5%)	560 (10.6%)	1329 (14.7%)
	Mean*	4.16	3.57	3.89	3.63	
Doctors should always wear a white coat when seeing patients in any setting.	Strongly disagree	23 (2.5%)	109 (5.4%)	179 (21.9%)	181 (3.4%)	492 (5.4%)
	Disagree	119 (12.7%)	567 (28.2%)	164 (20.0%)	1140 (21.5%)	1990 (22.0%)
	Neither agree nor disagree	269 (28.7%)	682 (33.9%)	202 (24.7%)	2147 (40.6%)	3300 (36.4%)
	Agree	361 (38.5%)	550 (27.4%)	169 (20.7%)	1497 (28.3%)	2577 (28.5%)
	Strongly agree	165 (17.6%)	103 (5.1%)	104 (12.7%)	326 (6.2%)	698 (7.7%)
	Mean*	3.56	2.99	2.82	3.12	

* Means calculated with scores of 1 assigned to “strongly disagree,” 3 to “neither agree nor disagree,” and 5 to “strongly agree.”

For peer review only

FIGURE LEGEND

***Figure 1*: Mean composite ratings of physician attire**

For peer review only

For peer review only - http://bmjopen.bmj.com/site/about/guidelines.xhtml

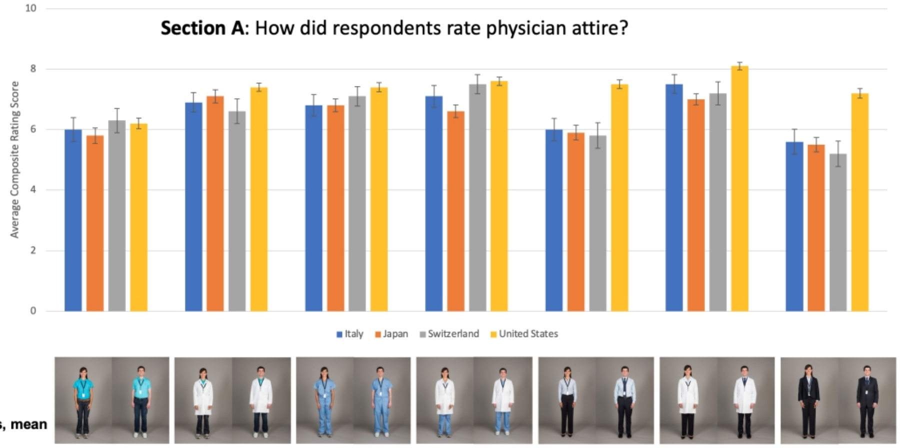

Countries, mean (SD)							
Italy	6.0 (2.3)	6.9 (1.8)	6.8 (2.1)	7.1 (2.0)	6.0 (2.2)	7.5 (1.8)	5.6 (2.4)
Japan	5.8 (2.2)	7.1 (1.8)	6.8 (1.8)	6.6 (1.8)	5.9 (2.1)	7.0 (1.6)	5.5 (2.1)
Switzerland	6.3 (2.2)	6.6 (2.3)	7.1 (1.7)	7.5 (1.7)	5.8 (2.3)	7.2 (1.9)	5.2 (2.2)
United States	6.2 (2.5)	7.4 (2.0)	7.4 (2.0)	7.6 (2.0)	7.5 (2.0)	8.1 (1.8)	7.2 (2.2)

Mean composite ratings of physician attire

443x261mm (144 x 144 DPI)

Appendix A: Survey Photographs by Country

	Casual	Casual with white coat	Scrubs	Scrubs with white coat	Formal	Formal with white coat	Business suit

Italy and the United States

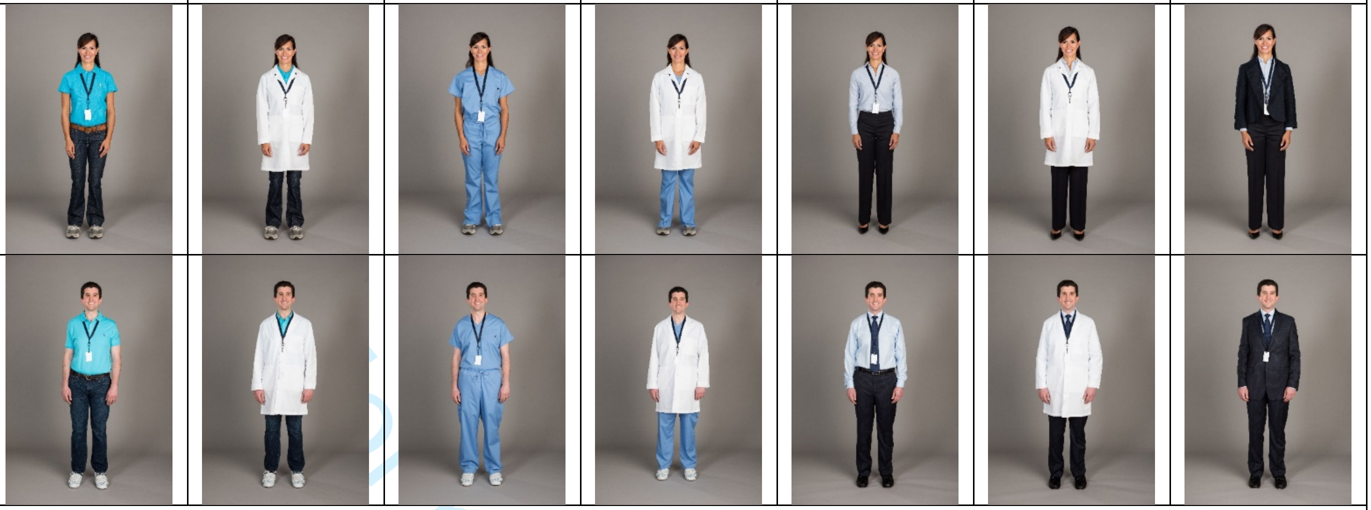

Switzerland

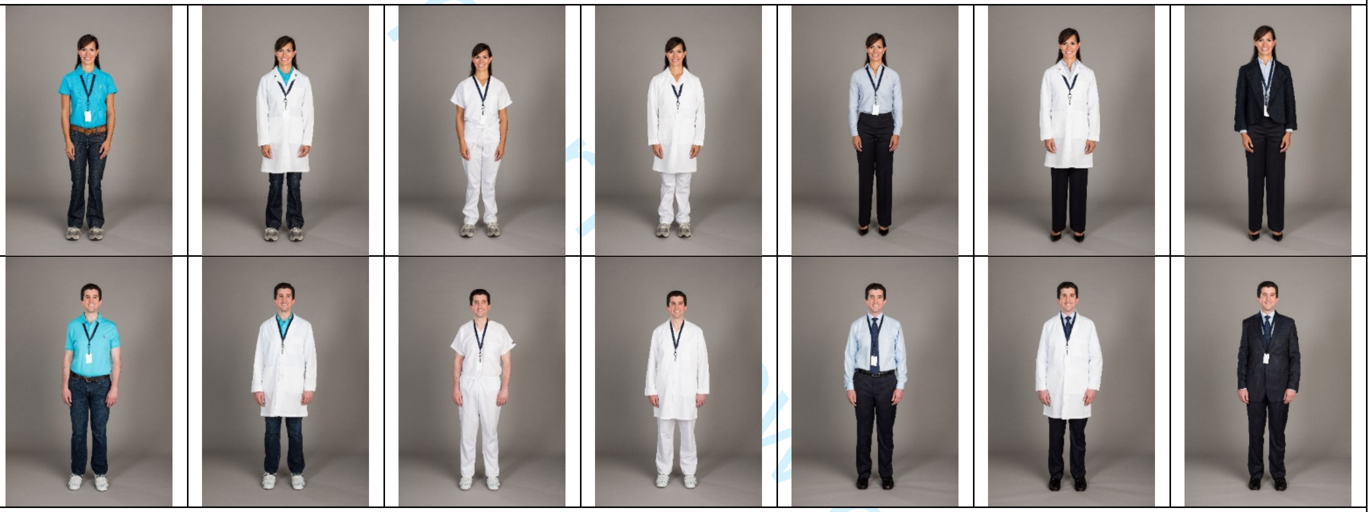

Japan

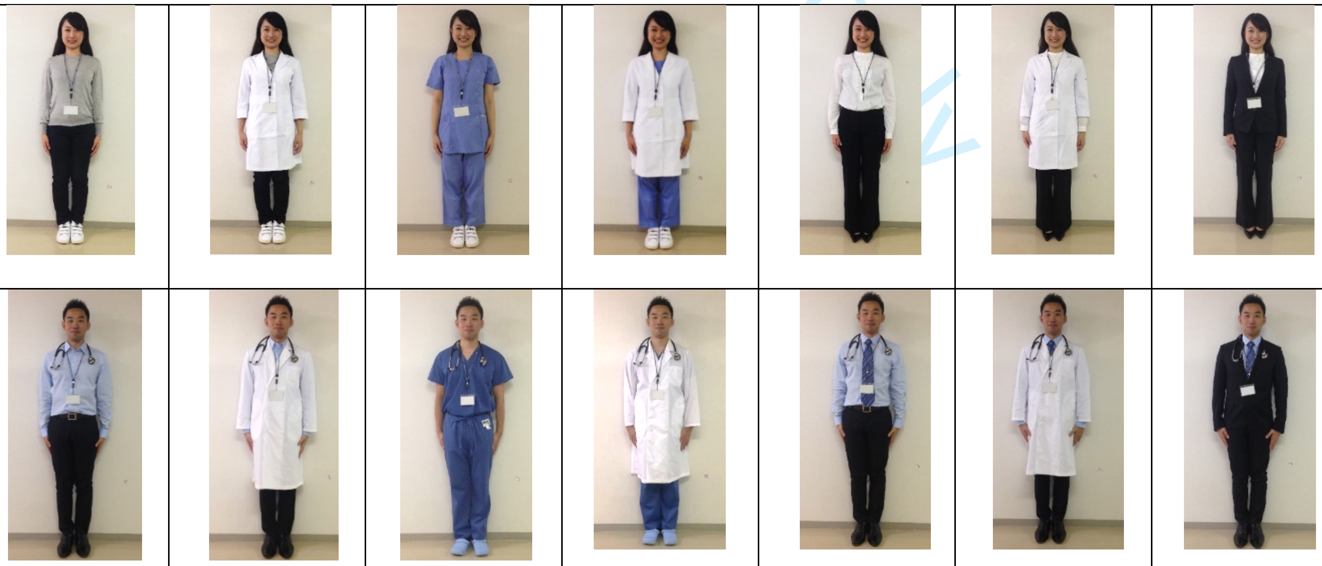

Appendix B: Survey Instrument

Section A – Physician Attire - Ratings

Please rate the doctor for each of the following questions by circling the number that corresponds to your answer.

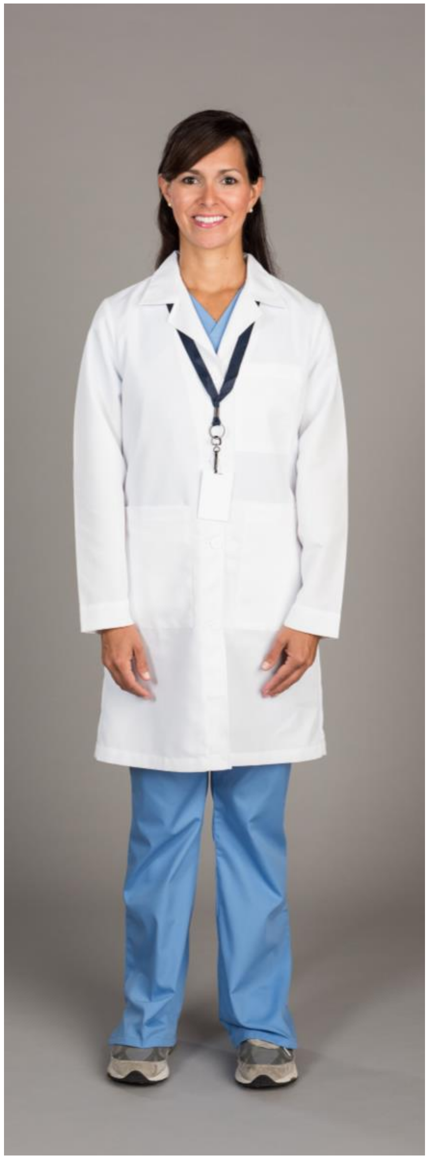	1) How **knowledgeable** does this doctor appear?	1 2 3 4 5 6 7 8 9 10<br>Somewhat Extremely
	2) How **trustworthy** does this doctor appear?	1 2 3 4 5 6 7 8 9 10<br>Somewhat Extremely
	3) How **caring** does this doctor appear?	1 2 3 4 5 6 7 8 9 10<br>Somewhat Extremely
	4) How **approachable** does this doctor appear?	1 2 3 4 5 6 7 8 9 10<br>Somewhat Extremely
	5) How **comfortable** does this doctor make you feel?	1 2 3 4 5 6 7 8 9 10<br>Somewhat Extremely

For peer review only

Section B – Physician Attire - Preferences

Please provide your ONE best answer to each of the following questions

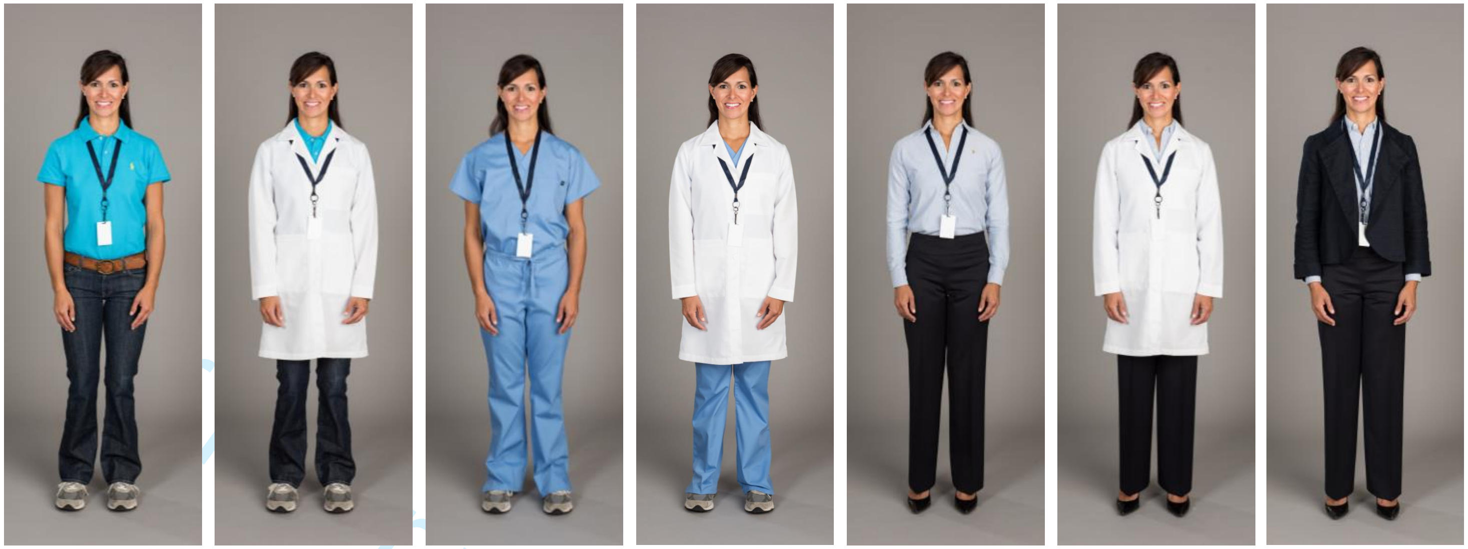

A B C D E F G

6) Which doctor would you prefer for your **primary care doctor**? (Please select only ONE option)

☐ **A** ☐ **B** ☐ **C** ☐ **D** ☐ **E** ☐ **F** ☐ **G**

7) Which doctor would you prefer to see when visiting the **emergency room**? (Please select only ONE option)

☐ **A** ☐ **B** ☐ **C** ☐ **D** ☐ **E** ☐ **F** ☐ **G**

8) Which doctor would you prefer to see when **in the hospital**? (Please select only ONE option)

☐ **A** ☐ **B** ☐ **C** ☐ **D** ☐ **E** ☐ **F** ☐ **G**

9) Which doctor would you prefer for your **surgeon**? (Please select only ONE option)

☐ **A** ☐ **B** ☐ **C** ☐ **D** ☐ **E** ☐ **F** ☐ **G**

10) **Overall**, which clothes do you feel doctors should wear? (Please select only ONE option)

☐ **A** ☐ **B** ☐ **C** ☐ **D** ☐ **E** ☐ **F** ☐ **G**

For peer review only - http://bmjopen.bmj.com/site/about/guidelines.xhtml

Section C – General Physician Attire

Please indicate your level of agreement with the following statements by checking ONE box to the left of your answer.

11) How my doctor dresses is important to me.

☐ Strongly Disagree ☐ Disagree ☐ Neither Agree nor Disagree ☐ Agree ☐ Strongly Agree

12) How my doctor dresses influences how happy I am with the care I receive.

☐ Strongly Disagree ☐ Disagree ☐ Neither Agree nor Disagree ☐ Agree ☐ Strongly Agree

13) It is appropriate for a doctor to dress casually when seeing patients **over the weekend.**

☐ Strongly Disagree ☐ Disagree ☐ Neither Agree nor Disagree ☐ Agree ☐ Strongly Agree

14) Doctors should wear a white coat when seeing patients **in their office or clinic**.

☐ Strongly Disagree ☐ Disagree ☐ Neither Agree nor Disagree ☐ Agree ☐ Strongly Agree

15) Doctors should wear a white coat when seeing patients in the **emergency room**.

☐ Strongly Disagree ☐ Disagree ☐ Neither Agree nor Disagree ☐ Agree ☐ Strongly Agree

16) Doctors should wear a white coat when seeing patients **in the hospital**.

☐ Strongly Disagree ☐ Disagree ☐ Neither Agree nor Disagree ☐ Agree ☐ Strongly Agree

17) Doctors should always wear a white coat when seeing patients **in any setting**.

☐ Strongly Disagree ☐ Disagree ☐ Neither Agree nor Disagree ☐ Agree ☐ Strongly Agree

For peer review only

D

Section D – Demographics

Please remember that all of your answers will be kept confidential.

18) How old are you?

☐ 18-25 ☐ 26-34 ☐ 35-54 ☐ 55-64 ☐ 65 or older

19) What is your gender?

☐ Male ☐ Female

20) What is the highest level of education you have completed?

☐ Less than High School ☐ High School ☐ Some College ☐ College ☐ Graduate Degree

21) What is your race?

☐ American Indian/Alaska Native ☐ Asian ☐ Native Hawaiian or Other Pacific Islander
☐ Black or African American ☐ White ☐ Hispanic
☐ Other (Please specify) ___________________________________

22) How many different doctors have you seen in the past year?

☐ 0 ☐ 1 ☐ 2 ☐ 3 ☐ 4 ☐ 5 ☐ 6 or more

Thank you for taking the time to fill out our survey.

Your input is greatly appreciated.

For peer review only

Appendix C. **Composite ratings of physician attire by domain**

Attire	Domain	Italy			Japan			Switzerland			United States		
		n	Mean	SD	n	Mean	SD	n	Mean	SD	n	Mean	SD
Casual	knowledgeable	137	5.2	2.5	285	5.3	2.4	118	5.6	2.4	752	5.4	2.7
	trustworthy	136	5.5	2.6	286	5.4	2.4	117	6.0	2.4	752	6.0	2.7
	caring	134	6.2	2.4	286	6.2	2.3	119	6.4	2.4	751	6.4	2.6
	approachable	137	6.7	2.3	286	6.5	2.3	119	7.1	2.2	752	6.7	2.6
	comfort	138	6.4	2.8	286	5.8	2.4	117	6.4	2.4	754	6.3	2.8
	mean score	133	6.0	2.3	285	5.8	2.2	115	6.3	2.2	748	6.2	2.5
Casual with white coat	knowledgeable	133	6.3	2.1	288	6.7	2.1	125	6.1	2.4	759	7.2	2.2
	trustworthy	133	6.5	2.1	288	6.8	2.1	124	6.5	2.4	757	7.4	2.2
	caring	133	7.1	2.0	288	7.3	1.9	122	6.6	2.5	759	7.5	2.1
	approachable	133	7.4	1.9	288	7.5	1.9	124	7.1	2.4	764	7.7	2.1
	comfort	133	7.2	2.0	288	7.1	2.1	123	6.5	2.5	759	7.5	2.2
	mean score	133	6.9	1.8	288	7.1	1.8	121	6.6	2.3	747	7.4	2.0
Scrubs	knowledgeable	136	6.2	2.4	283	6.3	2.1	114	6.8	2.0	747	7.0	2.3
	trustworthy	135	6.4	2.3	283	6.5	2.1	116	7.2	2.0	747	7.3	2.2
	caring	134	6.9	2.2	283	7.0	1.9	115	7.0	2.0	746	7.5	2.1
	approachable	136	7.2	2.1	283	7.2	1.8	115	7.4	1.7	749	7.7	2.1
	comfort	136	7.1	2.4	283	6.8	2.0	114	7.1	2.1	749	7.5	2.3
	mean score	134	6.8	2.1	283	6.8	1.8	113	7.1	1.7	742	7.4	2.0
Scrubs with white coat	knowledgeable	126	6.7	2.2	288	6.1	2.0	122	7.1	2.0	761	7.5	2.1
	trustworthy	128	6.9	2.3	290	6.2	2.0	122	7.5	2.1	759	7.6	2.1
	caring	126	7.1	2.3	290	6.8	2.0	121	7.4	2.0	757	7.6	2.1
	approachable	127	7.4	2.0	290	7.2	2.0	120	7.8	1.9	761	7.8	2.1
	comfort	128	7.3	2.2	290	6.6	2.1	121	7.5	1.9	760	7.7	2.2
	mean score	125	7.1	2.0	288	6.6	1.8	120	7.5	1.7	753	7.6	2.0
Formal	knowledgeable	137	5.6	2.4	286	5.5	2.3	121	5.6	2.4	759	7.4	2.1
	trustworthy	137	5.7	2.4	285	5.5	2.3	121	6.0	2.3	759	7.5	2.1

For peer review only

	caring	136	6.1	2.5	286	6.1	2.1	119	5.8	2.6	756	7.5	2.1
	approachable	137	6.5	2.3	286	6.3	2.2	121	6.0	2.6	763	7.7	2.1
	comfort	137	6.1	2.5	286	5.8	2.3	121	5.7	2.5	761	7.5	2.2
	mean score	136	6.0	2.2	285	5.9	2.1	119	5.8	2.3	754	7.5	2.0
Formal with white coat	knowledgeable	131	7.2	2.1	284	6.6	1.9	102	7.4	2.0	764	8.2	1.9
	trustworthy	130	7.4	2.0	284	6.7	1.9	101	7.4	2.0	761	8.2	1.9
	caring	131	7.6	1.9	284	7.4	1.7	101	7.1	2.1	759	8.0	1.9
	approachable	131	7.8	1.8	284	7.4	1.8	102	7.2	2.1	758	8.1	1.9
	comfort	130	7.7	1.8	284	7.0	1.8	101	7.0	2.3	758	8.1	2.0
	mean score	130	7.5	1.8	284	7.0	1.6	101	7.2	1.9	754	8.1	1.8
Business suit	knowledgeable	131	5.5	2.6	295	5.3	2.2	110	5.2	2.5	755	7.4	2.3
	trustworthy	129	5.7	2.5	295	5.4	2.2	109	5.4	2.5	755	7.3	2.3
	caring	130	5.6	2.5	296	5.8	2.2	110	5.0	2.4	754	7.1	2.4
	approachable	128	5.8	2.6	296	5.8	2.3	110	5.4	2.5	753	7.2	2.4
	comfort	131	5.5	2.8	295	5.4	2.3	109	5.2	2.5	755	7.0	2.5
	mean score	128	5.6	2.4	295	5.5	2.1	108	5.2	2.2	751	7.2	2.2

For peer review only

Appendix D. Comparisons of patient preferences for physician attire by type of attire between countries

	Casual				Casual + White Coat				Scrubs			
Location Comparison	Mean difference	Simultaneous 95% confidence limits		sig	Mean difference	Simultaneous 95% confidence limits		sig	Mean difference	Simultaneous 95% confidence limits		sig
Italy-Japan	0.2049	-0.4354	0.8452		-0.1829	-0.714	0.3481		0.0028	-0.5287	0.5343	
Italy-US	-0.1454	-0.7192	0.4284		-0.5303	-1.007	-0.0535	***	-0.6422	-1.118	-0.1665	***
Italy-Swiss	-0.3137	-1.0902	0.4627		0.3128	-0.3236	0.9492		-0.3083	-0.9557	0.3391	
Japan-US	-0.3503	-0.7748	0.0742		-0.3473	-0.6987	0.004		-0.645	-0.9992	-0.2909	***
Japan-Swiss	-0.5186	-1.1923	0.155		0.4957	-0.0531	1.0445		-0.3111	-0.8751	0.253	
US-Swiss	-0.1683	-0.7791	0.4424		0.843	0.3466	1.3394	***	0.3339	-0.1779	0.8458	

	Scrubs + White Coat				Formal				Formal + White Coat				Suit			
Location Comparison	Mean difference	Simultaneous 95% confidence limits		sig	Mean difference	Simultaneous 95% confidence limits		sig	Mean difference	Simultaneous 95% confidence limits		sig	Mean difference	Simultaneous 95% confidence limits		sig
Italy-Japan	0.46551	-0.0631	0.99412		0.1275	-0.4215	0.6765		0.53538	0.05998	1.01079	***	0.101	-0.4955	0.6975	
Italy-US	-0.58969	-1.06635	-0.11303	***	-1.553	-2.0438	-1.0622	***	-0.57297	-0.99932	-0.14662	***	-1.5514	-2.0903	-1.0125	***
Italy-Swiss	-0.43477	-1.06551	0.19598		0.1381	-0.5231	0.7994		0.3136	-0.28188	0.90908		0.4027	-0.3337	1.139	
Japan-US	-1.0552	-1.39714	-0.71326	***	-1.6805	-2.0468	-1.3142	***	-1.10836	-1.42093	-0.79579	***	-1.6523	-2.0396	-1.2651	***
Japan-Swiss	-0.90028	-1.43652	-0.36404	***	0.0106	-0.5643	0.5856		-0.22178	-0.7419	0.29834		0.3017	-0.3321	0.9355	
US-Swiss	0.15492	-0.33018	0.64003		1.6911	1.1715	2.2108	***	0.88657	0.41088	1.36227	***	1.954	1.3741	2.534	***

Sig, ***: Statistically significant

For peer review only

Appendix E. Comparisons of respondent opinions regarding importance, influence, and appropriateness of physician attire and white coats between countries

	Important				Influence				Casual weekend			
Location Comparison	Mean difference	Simultaneous 95% confidence limits		sig	Mean difference	Simultaneous 95% confidence limits		sig	Mean difference	Simultaneous 95% confidence limits		sig
Italy-Japan	0.03935	-0.06317	0.14187		-0.29709	-0.40417	-0.19002	***	0.57316	0.46985	0.67648	***
Italy-US	0.05486	-0.03705	0.14677		-0.24249	-0.33851	-0.14646	***	-0.12125	-0.21387	-0.02864	***
Italy-Swiss	0.49847	0.37459	0.62235	***	0.44933	0.32	0.57867	***	-0.22814	-0.35336	-0.10292	***
Japan-US	0.01551	-0.05221	0.08323		0.05461	-0.01601	0.12522		-0.69442	-0.76271	-0.62613	***
Japan-Swiss	0.45912	0.35195	0.56629	***	0.74643	0.63466	0.8582	***	-0.8013	-0.90977	-0.69283	***
US-Swiss	0.44361	0.34655	0.54067	***	0.69182	0.59059	0.79306	***	-0.10689	-0.20522	-0.00856	***

	White coat office				White coat ER				White coat hospital				White coat any setting			
Location Comparison	Mean difference	Simultaneous 95% confidence limits		sig	Mean difference	Simultaneous 95% confidence limits		sig	Mean difference	Simultaneous 95% confidence limits		sig	Mean difference	Simultaneous 95% confidence limits		sig
Italy-Japan	0.33013	0.23509	0.42516	***	1.01157	0.91526	1.10788	***	0.599	0.51144	0.68657	***	0.57579	0.47487	0.67671	***
Italy-US	0.38806	0.30286	0.47326	***	0.72743	0.64117	0.81369	***	0.53173	0.45328	0.61019	***	0.43908	0.34865	0.52952	***
Italy-Swiss	0.65738	0.5425	0.77226	***	0.04456	-0.072	0.16113		0.27093	0.16489	0.37697	***	0.73863	0.61654	0.86072	***
Japan-US	0.05793	-0.00488	0.12075		-0.28414	-0.34798	-0.22031	***	-0.06727	-0.12529	-0.00925	***	-0.1367	-0.20354	-0.06986	***
Japan-Swiss	0.32726	0.22784	0.42667	***	-0.96701	-1.06811	-0.8659	***	-0.32808	-0.42003	-0.23612	***	0.16284	0.05703	0.26865	***
US-Swiss	0.26933	0.17926	0.35939	***	-0.68287	-0.77444	-0.59129	***	-0.26081	-0.34414	-0.17748	***	0.29954	0.20369	0.3954	***

Sig, ***: Statistically significant

Important: How my doctor dresses is important to me.

Influence: How my doctor dresses influences how happy I am with the care I receive.

Casual weekend: It is appropriate for a doctor to dress casually when seeing patients over the weekend.

White coat office: Doctors should wear a white coat when seeing patients in their office.

White coat ER: Doctors should wear a white coat when seeing patients in the emergency room.

White coat hospital: Doctors should wear a white coat when seeing patients in the hospital.

White coat any setting: Doctors should always wear a white coat when seeing patients in any setting.

For peer review only

Appendix F. **Composite scores by respondent gender**

Attire	Italy			Japan			Switzerland			United States		
	Male	Female	P	Male	Female	P	Male	Female	P	Male	Female	P
Casual	6.0	6.1	0.77	6.0	5.6	0.13	6.5	6.0	0.21	6.3	6.0	0.10
Casual with white coat	7.0	6.9	0.85	7.2	7.0	0.40	6.5	6.6	0.90	7.3	7.5	0.16
Scrubs	6.5	6.9	0.34	6.8	6.8	0.93	7.2	6.9	0.38	7.4	7.5	0.71
Scrubs with white coat	7.3	6.9	0.26	6.5	6.6	0.60	7.5	7.5	0.96	7.6	7.7	0.41
Formal	5.6	6.3	0.09	6.0	5.7	0.28	6.2	5.4	0.04*	7.6	7.4	0.23
Formal with white coat	7.5	7.6	0.73	7.0	7.0	0.77	7.3	7.1	0.55	8.1	8.1	0.94
Business suit	5.5	5.8	0.52	5.6	5.4	0.41	5.1	5.2	0.74	7.1	7.3	0.38

* Statistically significant

For peer review only

Appendix G. **Composite scores by respondent age**

Attire	Italy						Japan					
	18-25	26-34	35-54	55-64	65+	P	18-25	26-34	35-54	55-64	65+	P
Casual	4.8	5.6	6.1	6.4	6.2	0.40	5.4	4.6	5.2	5.4	6.3	0.001*
Casual with white coat	8.1	6.5	6.4	7.1	7.0	0.06	8.0	7.0	6.6	6.7	7.4	0.003*
Scrubs	6.4	7.6	6.5	7.0	6.7	0.63	6.0	5.9	6.6	6.6	7.0	0.07
Scrubs with white coat	7.5	7.4	6.4	7.3	7.5	0.12	7.3	6.9	6.3	6.4	6.7	0.37
Formal	5.7	6.3	5.8	6.0	6.0	0.95	6.1	5.1	5.3	5.5	6.4	0.002*
Formal with white coat	7.9	7.3	7.6	7.3	7.7	0.76	7.5	6.6	6.6	6.8	7.3	0.01*
Business suit	4.7	7.1	5.7	5.3	5.4	0.12	5.0	4.8	5.0	5.2	6.1	<0.001*
	Switzerland						United States					
	18-25	26-34	35-54	55-64	65+	P	18-25	26-34	35-54	55-64	65+	P
Casual	7.7	6.6	6.3	6.4	5.9	0.72	5.9	6.3	5.8	6.1	6.5	0.09
Casual with white coat	7.2	7.5	6.6	6.1	6.2	0.27	8.0	7.6	7.2	7.3	7.6	0.03*
Scrubs	7.3	6.4	6.9	7.5	7.5	0.35	8.1	7.9	7.2	7.2	7.6	0.01*
Scrubs with white coat	8.5	7.8	7.1	7.5	8.0	0.10	7.9	7.7	7.7	7.5	7.7	0.73

For peer review only

Formal	5.9	5.2	5.8	6.3	5.8	0.52	8.3	7.5	7.2	7.5	7.8	0.003*
Formal with white coat	6.8	6.1	7.3	7.9	6.9	0.20	8.2	7.8	8.0	8.1	8.3	0.15
Business suit	5.8	4.4	5.2	5.4	5.4	0.69	7.1	7.2	7.0	7.1	7.4	0.28

* Statistically significant

For peer review only

STROBE Statement—checklist of items that should be included in reports of observational studies

	Item No	Recommendation	Page/Lines
**Title and abstract**	1	(*a*) Indicate the study's design with a commonly used term in the title or the abstract	1/1-2<br>4/5-7
		(*b*) Provide in the abstract an informative and balanced summary of what was done and what was found	4/2-23<br>5/1-5
**Introduction**			
Background/rationale	2	Explain the scientific background and rationale for the investigation being reported	6/11-20
Objectives	3	State specific objectives, including any prespecified hypotheses	6/21-23<br>7/1-5
**Methods**			
Study design	4	Present key elements of study design early in the paper	7/9-12
Setting	5	Describe the setting, locations, and relevant dates, including periods of recruitment, exposure, follow-up, and data collection	7/9-15<br>Table 1
Participants	6	(*a*) *Cohort study*—Give the eligibility criteria, and the sources and methods of selection of participants. Describe methods of follow-up<br>*Case-control study*—Give the eligibility criteria, and the sources and methods of case ascertainment and control selection. Give the rationale for the choice of cases and controls<br>***Cross-sectional study***—Give the eligibility criteria, and the sources and methods of selection of participants	7/16-23<br>10/20-21
		(*b*) *Cohort study*—For matched studies, give matching criteria and number of exposed and unexposed<br>*Case-control study*—For matched studies, give matching criteria and the number of controls per case	--
Variables	7	Clearly define all outcomes, exposures, predictors, potential confounders, and effect modifiers. Give diagnostic criteria, if applicable	8/10-23<br>9/1-9<br>10/1-5<br>Appendix A<br>Appendix B
Data sources/ measurement	8*	For each variable of interest, give sources of data and details of methods of assessment (measurement). Describe comparability of assessment methods if there is more than one group	9/12-23
Bias	9	Describe any efforts to address potential sources of bias	9/5-9
Study size	10	Explain how the study size was arrived at	7/9-10
Quantitative variables	11	Explain how quantitative variables were handled in the analyses. If applicable, describe which groupings were chosen and why	10/8-10
Statistical methods	12	(*a*) Describe all statistical methods, including those used to control for confounding	10/8-16
		(*b*) Describe any methods used to examine subgroups and interactions	10/11-15
		(*c*) Explain how missing data were addressed	9/22-23<br>10/8-9

For peer review only - http://bmjopen.bmj.com/site/about/guidelines.xhtml

(*d*) *Cohort study*—If applicable, explain how loss to follow-up was addressed<br>*Case-control study*—If applicable, explain how matching of cases and controls was addressed<br>***Cross-sectional study***—If applicable, describe analytical methods taking account of sampling strategy	N/A
(*e*) Describe any sensitivity analyses	N/A

Continued on next page

For peer review only

For peer review only - http://bmjopen.bmj.com/site/about/guidelines.xhtml

Results			Page/Line
Participants	13*	(a) Report numbers of individuals at each stage of study—eg numbers potentially eligible, examined for eligibility, confirmed eligible, included in the study, completing follow-up, and analysed	11/8-10
		(b) Give reasons for non-participation at each stage	N/A
		(c) Consider use of a flow diagram	N/A
Descriptive data	14*	(a) Give characteristics of study participants (eg demographic, clinical, social) and information on exposures and potential confounders	11/10-16<br>Table 2<br>Appendix A
		(b) Indicate number of participants with missing data for each variable of interest	Table 2<br>Table 3<br>Table 4<br>Appendix C
		(c) *Cohort study*—Summarise follow-up time (eg, average and total amount)	--
Outcome data	15*	*Cohort study*—Report numbers of outcome events or summary measures over time	--
		Case-control study—Report numbers in each exposure category, or summary measures of exposure	--
		Cross-sectional study—Report numbers of outcome events or summary measures	N/A
Main results	16	(*a*) Give unadjusted estimates and, if applicable, confounder-adjusted estimates and their precision (eg, 95% confidence interval). Make clear which confounders were adjusted for and why they were included	**Primary Outcome:**<br>11/19-23<br>12/1-8<br>Figure 1<br>Appendix C
		(*b*) Report category boundaries when continuous variables were categorized	11/10<br>15/21
		(*c*) If relevant, consider translating estimates of relative risk into absolute risk for a meaningful time period	N/A
Other analyses	17	Report other analyses done—eg analyses of subgroups and interactions, and sensitivity analyses	**Secondary Outcomes:**<br>12/11-22<br>13/1-22<br>14/1-23<br>15/1-23<br>16/1-6<br>Tables 3, 4<br>Appendices D-G
**Discussion**			
Key results	18	Summarise key results with reference to study objectives	16/9-16
Limitations	19	Discuss limitations of the study, taking into account sources of potential bias or imprecision. Discuss both direction and magnitude of any potential bias	19/3-23<br>20/1-5
Interpretation	20	Give a cautious overall interpretation of results considering	16/17-23

For peer review only

For peer review only - http://bmjopen.bmj.com/site/about/guidelines.xhtml

		objectives, limitations, multiplicity of analyses, results from similar studies, and other relevant evidence	17/1-21
Generalisability	21	Discuss the generalisability (external validity) of the study results	19/1-2<br>20/6-14
**Other information**			
Funding	22	Give the source of funding and the role of the funders for the present study and, if applicable, for the original study on which the present article is based	11/1-3

*Give information separately for cases and controls in case-control studies and, if applicable, for exposed and unexposed groups in cohort and cross-sectional studies.

Note: An Explanation and Elaboration article discusses each checklist item and gives methodological background and published examples of transparent reporting. The STROBE checklist is best used in conjunction with this article (freely available on the Web sites of PLoS Medicine at http://www.plosmedicine.org/, Annals of Internal Medicine at http://www.annals.org/, and Epidemiology at http://www.epidem.com/). Information on the STROBE Initiative is available at www.strobe-statement.org.

For peer review only
